# Natural Killer Cells Improve Hematopoietic Stem Cell Engraftment by Increasing Stem Cell Clonogenicity *In Vitro* and in a Humanized Mouse Model

**DOI:** 10.1371/journal.pone.0138623

**Published:** 2015-10-14

**Authors:** Michelle Escobedo-Cousin, Nicola Jackson, Raquel Laza-Briviesca, Linda Ariza-McNaughton, Martha Luevano, Sophie Derniame, Sergio Querol, Michael Blundell, Adrian Thrasher, Bernat Soria, Nichola Cooper, Dominique Bonnet, Alejandro Madrigal, Aurore Saudemont

**Affiliations:** 1 University College London, Cancer Institute, London, United Kingdom; 2 Anthony Nolan Research Institute, Royal Free Campus, London, United Kingdom; 3 Cancer Research UK, London Research Institute, London, United Kingdom; 4 Programa Concordia Banc de Sang i Teixits, Barcelona, Spain; 5 University College London, Institute of Child Health, London, United Kingdom; 6 Andalusian Center for Molecular Biology and Regenerative Medicine (CABIMER), Fundación Progreso y Salud, Seville, Spain; 7 Biomedical Research Network on Diabetes and Related Metabolic Diseases (CIBERDEM), Instituto de Salud Carlos III, Madrid, Spain; 8 Haematology Department, Hammersmith Hospital, London, United Kingdom; Rutgers - New Jersey Medical School, UNITED STATES

## Abstract

Cord blood (CB) is increasingly used as a source of hematopoietic stem cells (HSC) for transplantation. Low incidence and severity of graft-versus-host disease (GvHD) and a robust graft-versus-leukemia (GvL) effect are observed following CB transplantation (CBT). However, its main disadvantages are a limited number of HSC per unit, delayed immune reconstitution and a higher incidence of infection. Unmanipulated grafts contain accessory cells that may facilitate HSC engraftment. Therefore, the effects of accessory cells, particularly natural killer (NK) cells, on human CB HSC (CBSC) functions were assessed *in vitro* and *in vivo*. CBSC cultured with autologous CB NK cells showed higher levels of CXCR4 expression, a higher migration index and a higher number of colony forming units (CFU) after short-term and long-term cultures. We found that CBSC secreted CXCL9 following interaction with CB NK cells. In addition, recombinant CXCL9 increased CBSC clonogenicity, recapitulating the effect observed of CB NK cells on CBSC. Moreover, the co-infusion of CBSC with CB NK cells led to a higher level of CBSC engraftment in NSG mouse model. The results presented in this work offer the basis for an alternative approach to enhance HSC engraftment that could improve the outcome of CBT.

## Introduction

Cord blood transplantation (CBT) offers many advantages including rapid accessibility of the graft, less stringent HLA matching, a lower incidence of Graft versus Host Disease (GvHD) and a preserved Graft versus Leukemia (GvL) effect as compared to transplantation performed with bone marrow (BM) or mobilized peripheral blood (mPB) hematopoietic stem cells (HSC) [1, 2]. However, some of the main limitations of CBT are a lower number of nucleated cells per cord blood (CB) unit, delayed engraftment and immune reconstitution and a higher incidence of infection [3]. It has been described that the low HSC dose, the leukocyte content of the graft and ineffective CB HSC (CBSC) homing to the BM are factors associated with graft failure post-CBT [3–5]. Recipients that require low HSC doses, such as pediatric patients, are the best candidates to receive CB grafts, however, adult patients who require a higher HSC dose represent a greater challenge for CBT.

Double CBT, using two unmanipulated CB units, have been performed to increase the cell dose for adult patients^6^. This approach results in better engraftment, however long-term engraftment is generally derived from one unit only [6–9]. It has been described that the unit dominance is determined by CD34^-^ accessory cells from the CB graft [10], suggesting that accessory cells participate in the engraftment process. Whilst several studies have shown the importance of accessory cells present in the graft for optimal HSC engraftment [11–13], a specific cell type that can be used to improve engraftment has yet to be identified. It was reported that the co-transplantation of umbilical cord-derived mesenchymal stem cells with the corresponding CB unit results in better engraftment as compared to patients transplanted with the CB graft only [14]. Other groups have reported a positive correlation between T cell dose and engraftment in adult patients post-CBT [15, 16]. In humanized mice, T cell depleted CB grafts showed inferior levels of engraftment, which could be enhanced when T cells stimulated *ex vivo* were co-infused [17]. Similarly, a report using BM grafts showed that CD8^+^ T cells lacking cytotoxicity support initial HSC engraftment whereas CD8^+^ T cells with intact cytotoxic functions are needed to support long-term engraftment [18]. Moreover, a higher graft content of cytotoxic cells, CD8^+^ T cells and NK cells, correlated with early engraftment and better outcome after transplantation with mPB HSC [19]. Lastly, improved levels of engraftment were reported in mice that received donor NK cells and IL-15 in a mouse model of non-myeloablative allogeneic BM transplantation [20] and in patients following transplantation using a CD3/CD19 depleted graft [21].

HSC must migrate to the BM in order to engraft and facilitate long-term immune reconstitution. It is known that the CXCR4/SDF-1 axis, LFA-1 (CD11a), VLA-4 (CD29/CD49d) and VLA-5 (CD29/CD49e) all play key roles in HSC homing and maintenance within the BM niche [22–24]. In addition, it has been shown that CXCR7 may also be involved in this process through interaction with CXCR4 [25, 26]. Thus, efforts have been made to enhance HSC engraftment by improving HSC homing. Recently, it was shown that inhibition of CD26, the dipeptidylpeptidase IV (DPPIV) enzyme that cleaves and inactivates SDF-1α, results in enhanced migration of HSC *in vitro* and improved homing and engraftment of CBSC into lethally irradiated humanized mice [27, 28]. Likewise, HSC fucosylation improved CBSC homing and engraftment *in vivo* [5, 29]. Nevertheless, the effect of accessory cells on CBSC homing and engraftment requires further investigation.

Here, we analyzed the effect of accessory cells on CBSC engraftment in NSG mice and identified CB NK cells as a key population that influences CBSC engraftment *in vivo*. Co-culture of CB NK cells with CBSC increased CXCR4 expression on CBSC, which translated into enhanced chemotaxis towards SDF-1α *in vitro*. CB NK cells also enhanced CBSC clonogenic capacity as assessed by short-term and long-term *in vitro* cultures, potentially by inducing CXCL9 secretion by CBSC. The effect on clonogenic capacity was contact dependent as blocking of key integrins expressed by CBSC prevented the effect of CB NK cells. These data demonstrate a novel effect of CB NK cells on CBSC that could be utilized to improve the outcome of CBT.

## Materials and Methods

### Cord Blood Samples and Cell Purification

All CB samples were obtained with prior written consent and ethical committee approval from the Anthony Nolan Cord Blood bank (Research Ethics Committee reference 10/H0405/27). The study had full ethical approval from the Anthony Nolan and Royal Free Hospital Research Ethics Committee. CB mononuclear cells (CBMCs) were isolated by density gradient centrifugation using Ficoll-Paque PLUS (GE Healthcare). CBSC were isolated using the CD34 microbead kit (Miltenyi Biotec) [30] to a purity of 98.4% ± 0.75. CBSC purity was analyzed as CD133^+^CD34^+^CD45^low^ and following the International Society of Hematotherapy and Graft Engineering (ISHAGE) gating guidelines. CB NK cells were isolated using the NK cell isolation kit (Miltenyi Biotec), to a purity of 90.39% ± 3.35. When indicated, NK cells were activated for 4 h using 20 ng/mL IL-15 and CD69 expression was assessed on NK cells as a measure of activation. T cells were labeled with PE-conjugated CD4 or CD8 antibodies respectively and isolated from CB using anti-PE MultiSort MicroBeads (Miltenyi Biotec) with purities of 90.16% ± 0.76 and 81.66% ± 11.06 respectively. The function of CD4 and CD8 T cells was not analyzed post-isolation.

### Flow Cytometry

Cells were stained with fluorophore-conjugated antibodies at 4°C for 10 min (or for 45 min for anti-CXCR4 and anti-CXCR7 antibodies), washed and re-suspended in 1X PBS containing 10% FBS. A FACSCalibur flow cytometer (Becton Dickinson) or a LSRFortessa flow cytometer (Becton Dickinson) were used to acquire data and FlowJo software (TreeStar) was used for data analysis. The following monoclonal antibodies were purchased from BD Biosciences: CD3 (SK7), CD4 (SK3), CD8 (SK1), CD11a (HI111), CD29 (TS2/16), CD34 (581), CD44 (Bu52), CD45 (HI30), CD49d (9F10), CD49e (IIA1), CD49f (GoH3), CD56 (B159), CD69 (L78), CD133 (293C3), CD162 (KPL-1), CXCR4 (12G5), CXCR7 (358426), NKp44 (P44-8) and β7 integrin (12G5). Cell viability was assessed using Annexin V and 7AAD (BD Biosciences). For cell cycle analysis, cells were fixed with 70% Ethanol/30% PBS for at least 1 h at 4°C. The fixed cell pellet was then incubated for 10 min at RT with RNAse at 0.17 mg/mL. To stain the DNA, the cells were incubated for 1 h at 37°C with propidium iodide at 36 ug/mL and then analyzed by flow cytometry.

### Cell Culture

MS-5 cells were a kind gift of Dr Bonnet (CRUK, London). Cells were cultured in alpha-MEM with 10% FBS, 2 mM L-glutamine and 2 mM sodium pyruvate. CB NK cells were activated with 20 ng/mL IL-15 (Peprotech) in RPMI-1640 containing 10% FBS and 50 μM β-ME for 4 h. CBSC were cultured either alone or with resting NK (rNK) cells or activated NK (aNK) cells for 4 h at a ratio of 1 to 5 CBSC to CB NK cells.

### Colony Forming Unit Assays

CBSC were cultured with rNK cells or aNK cells or with CXCL9 (Peprotech) at different concentrations for 4 hrs. A minimum of 200 CBSC were plated in MethoCult GF H-84434 (Stemcell Technologies) and cultured for 14 days at 37°C, 5% CO_2_. Colony formation was enumerated using an inverted microscope at the end of culture. For integrin blocking experiments, CBSC were incubated with blocking antibodies (eBioscences) against β7 integrin (FIB504), CD11a (HI111), CD49d (9F10) and CD49e (IIA1) at 10 μg/mL for 1 h and then washed before co-culture with NK cells and CFU assays.

### Long Term Culture-Initiating Cell (LTC-IC) Assays

CBSC (500 per well) were seeded on a feeder layer of irradiated MS-5 cells (30 Gray) in alpha-MEM, 20% FBS, 10^−5^ M hydrocortisone, 50 μM β-ME, at ten replicates per condition, for either CBSC alone or co-cultured with rNK or aNK cells for 4 h. Wells were scored for cobblestone areas after 4 weeks and adherent cells were used to perform CFU assays as described.

### Migration Assays

Transwell migration assays were performed using 5 μm polycarbonate membrane HTS 96-well transwell plates (Corning) coated with fibronectin (20 μg/mL). CBSC were plated in the upper chamber of the transwell plate either alone or in the presence of rNK or aNK cells. Migration to the lower chamber was assessed by CFU assay after 4 h using a standard SDF-1α concentration of 125 ng/mL [31] or after 3 h using a sub-optimal concentration of 50 ng/mL [32]. The chemotactic index was calculated as the ratio of the number of cells that migrated towards SDF-1α to the number of cells that migrated towards medium only.

### Microarray Analysis

CBSC and rNK or aNK cells were co-cultured for 4 h and then stained with anti-CD34-PE (BD Biosciences) for re-isolation of CBSC (as CD34^+^ cells) by sorting using a FACSAria cell sorter (BD Biosciences). RNA was isolated from sorted CBSC, rNK or aNK cells using the RNeasy Micro kit (Qiagen). Samples were processed using GeneChip Whole Transcript Sense Target Labeling assays using the Ambion WT Expression kit and Affymetrix GeneChip WT Terminal Labeling and Controls kit (Affymetrix). The resulting ssDNAs were hybridized to the GeneChip human Gene 2.0 ST Array (Affymetrix) and microarray analysis was performed by the UCL Genomics Facility, Institute of Child Health (London, UK). Image reads were processed using Affymetrix software and background was corrected and normalized using the RMA algorithm with GeneSpring software (Agilent Technologies). Differentially expressed genes were analyzed using GeneSpring software. Data are available from the EMBL-EBI/ArrayExpress repository under accession E-MTAB-2531 for the CBSC analysis and E-MTAB-2847 for the NK cell analysis.

### ELISA

Human CXCL9 was quantified in culture supernatants from co-cultures between CBSC and NK cells using the Human MIG Instant ELISA (eBioscience). No CXCL9 secretion was detected from cultures of NK cells alone. Human IFN-γ and TNF-β secretion following NK cell activation were measured using the corresponding Human Instant ELISA (eBioscience).

### CBSC Engraftment in NSG Mice

NOD/SCID IL-2Rγ^null^ (NSG) mice (males and females, 8 to 10 weeks old) were irradiated with 3.75 Gray. Irradiated NSG mice were injected intravenously 24 h later with 20,000 CD34^+^ CBSC alone or with accessory cells from the same CB unit at a ratio of 5 to 1 accessory cells to CBSC. After 10 weeks, the level of CBSC engraftment in the BM was assessed for each mouse by analysis of human CD45 expression using flow cytometry [33]. Animal experiments were performed according to the recommendations of the UK Home Office Regulations, protocols were approved by the UK Home Office (project license 80/2456). For each experiment, 4–6 mice were used per group. Mice were randomly assigned to each group. All efforts were made to minimize suffering. Mice were humanely culled by cervical dislocation.

### Statistical Analysis

Statistical analysis was performed using Prism 5 (GraphPad Software Inc., USA). Data are presented as means and SD. Paired or unpaired *t* tests were used to analyze results obtained from *in vitro* and *in vivo* experiments respectively. P values less than 0.05 were considered statistically significant.

## Results

### CB NK Cells Enhance CBSC Engraftment in NSG Mice

**Fig 1:**
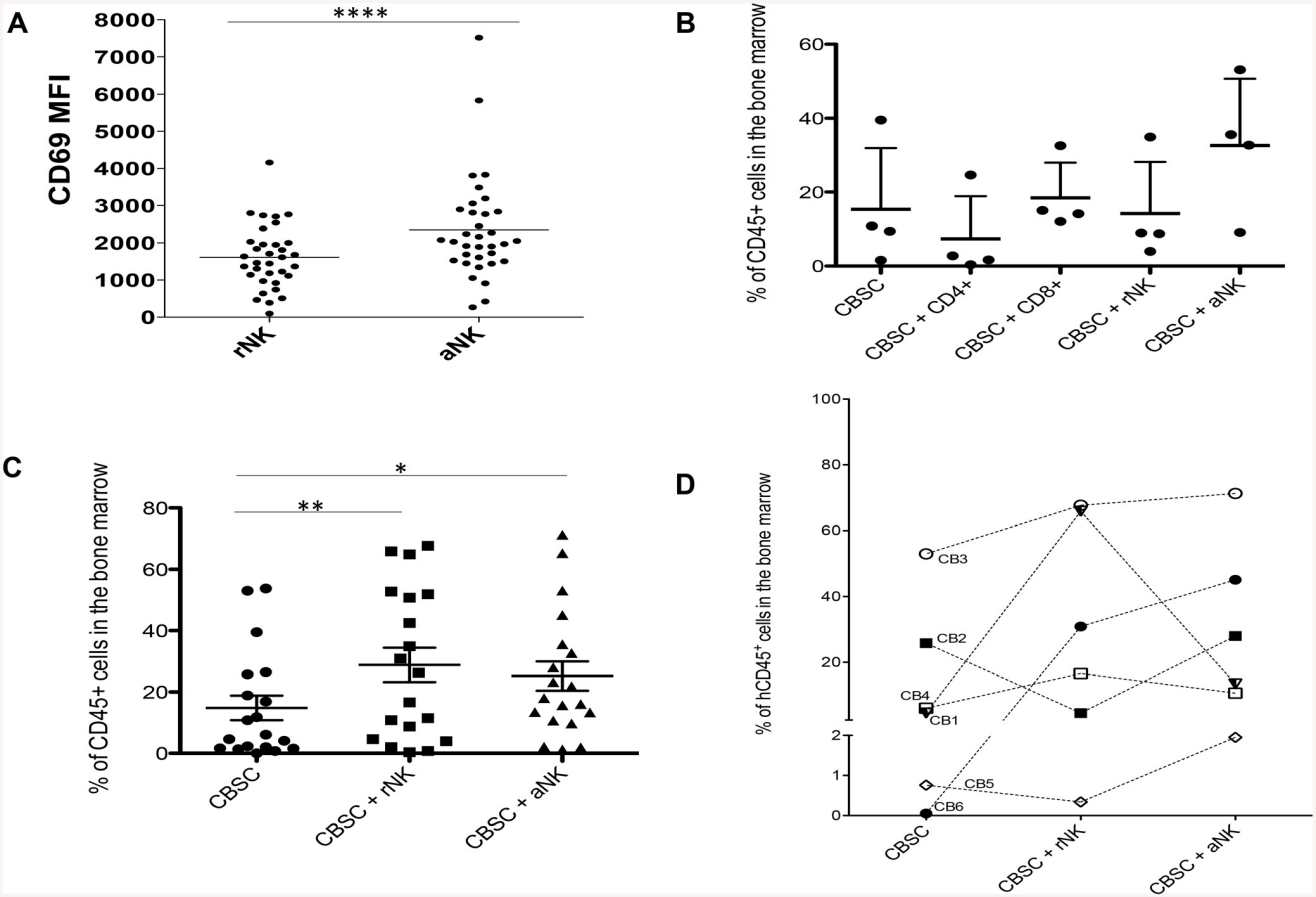
Level of hCD45+ Cell Engraftment in the BM and Fold Increase in Engraftment Observed in NSG Mice Transplanted with CBSC and CB NK Cells. (**A**) CD69 expression measured as mean fluorescence intensity (MFI) on CB NK cells before and after incubation with 20 ng/mL IL-15 for 4 h (N = 18). (**B**) Percentage of hCD45^+^ cells detected by flow cytometry in the BM of NSG mice ten weeks post-transplant with CBSC alone or in combination with CD4+ T cells, CD8+ T cells, rNK cells or aNK cells (N = 4). (**C**) Percentage of hCD45^+^ cells detected by flow cytometry in the BM of NSG mice ten weeks post-transplant with CBSC alone or in combination with rNK cells or aNK cells (N = 14). (**D**) Percentage of hCD45^+^ cells detected by flow cytometry in the BM of NSG mice transplanted with CBSC alone or in combination with rNK cells or aNK cells from 6 different CB units (N = 18). * P < 0.05, ** P < 0.01, *** P < 0.001.

We first analyzed the effects of NK cells, CD4^+^ T cells and CD8^+^ T cells isolated from the same CB unit on CBSC engraftment in NSG mice. For this study, resting cells were used except for where we compared resting (rNK) or activated (aNK) NK cells, as it has been previously shown that the infusion of NK cells together with IL-15 improved HSC engraftment in a mouse model of transplantation [34]. Moreover, we have previously identified that higher concentration of IL-2 was required to activate CB NK cells as compared to PB NK cells [35] and that IL-15 led to a better activation of CB NK cells as compared to IL-2 [36]. We chose to treat CB NK cells with IL-15 (20 ng/mL) for 4 h at the lowest concentration we previously tested [36] as this led to significant NK cell activation as shown by upregulation of the activation marker CD69 (p < 0.0001) (Fig 1A), without altering their cytolytic function or expression of activating or inhibitory receptors (data not shown). We found that mice transplanted with CBSC and aNK cells showed higher levels of hCD45+ cell engraftment in the BM compared to mice that received any of the other cell combinations (Fig 1B), identifying CB NK cells as a key population that influences CBSC engraftment *in vivo*.

We therefore chose to focus on the effects of CB NK cells on CBSC thereafter. However, upon further analysis of the effects of NK cells on CBSC *in vivo*, we found that CBSC constantly engrafted better in the presence of CB NK cells, either rNK cells (p < 0.05) or aNK cells (p < 0.05), when compared to NSG mice infused with CBSC only (Fig 1C). In order to assess the effect of CB NK cells on CBSC, taking into account the variability between research grade CB units, as well as the natural variability in NK cell activation state, the same experiment was performed using six different CB units and co-culturing CBSC with rNK cells or aNK cells for 4 h prior to infusion into NSG mice. Variable levels of engraftment were obtained from each CB unit; however, higher levels of engraftment were systematically observed in mice that received CBSC plus either rNK cells or aNK cells with all the CB units used (Fig 1D). Notably, we didn’t observe any correlation between levels of engraftment and levels of CD69 on rNK or aNK cells (data not shown).

### CB NK Cells Modify the Homing Receptor Repertoire of CBSC

**Fig 2:**
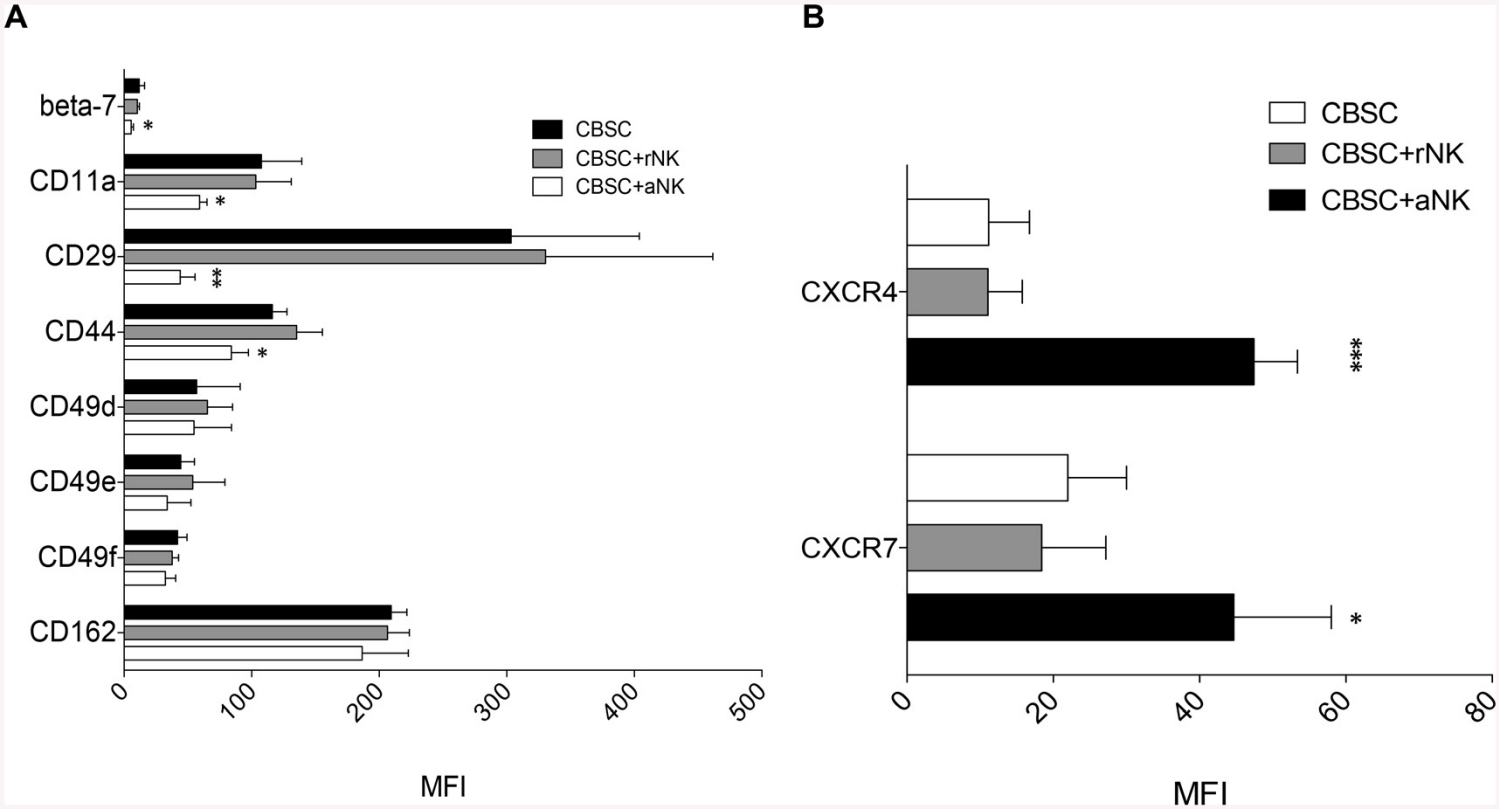
Expression of Homing Receptors by CBSC Following 4 h Co-culture with Resting and Activated NK Cells. Results are expressed as MFI ± SD of positive cells for alpha integrins CD49d, CD49e, CD49f and CD11a; beta integrins CD29 and beta-7; adhesion molecules CD162 and CD44 (**A**), and chemokine receptors CXCR4 and CXCR7 (**B**) (N = 4). Fluorescence minus one samples were used as a negative control, * P < 0.05, ** P < 0.01, *** P < 0.001.

We next assessed whether CB NK cells affect the homing properties of CBSC by analyzing the expression of key adhesion molecules and chemokine receptors for homing to the BM. Receptor expression by CBSC was assessed by flow cytometry following 4 h co-culture with rNK or aNK cells. Importantly, co-culture had no effect on CBSC or NK cell viability (data not shown). We found that CBSC expressed significantly lower levels of CD11a (p < 0.05), CD29 (p < 0.01), CD44 (p < 0.05) and integrin beta-7 (p < 0.05) after co-culture with aNK cells but not with rNK cells (Fig 2A). Interestingly, we observed significantly higher expression levels of the chemokine receptors CXCR4 (p < 0.001) and CXCR7 (p < 0.05) on CBSC co-cultured with aNK cells compared to CBSC alone or co-cultured with rNK cells (Fig 2B).

### Activated NK Cells Enhance CBSC Migration towards SDF-1α *In Vitro*

**Fig 3:**
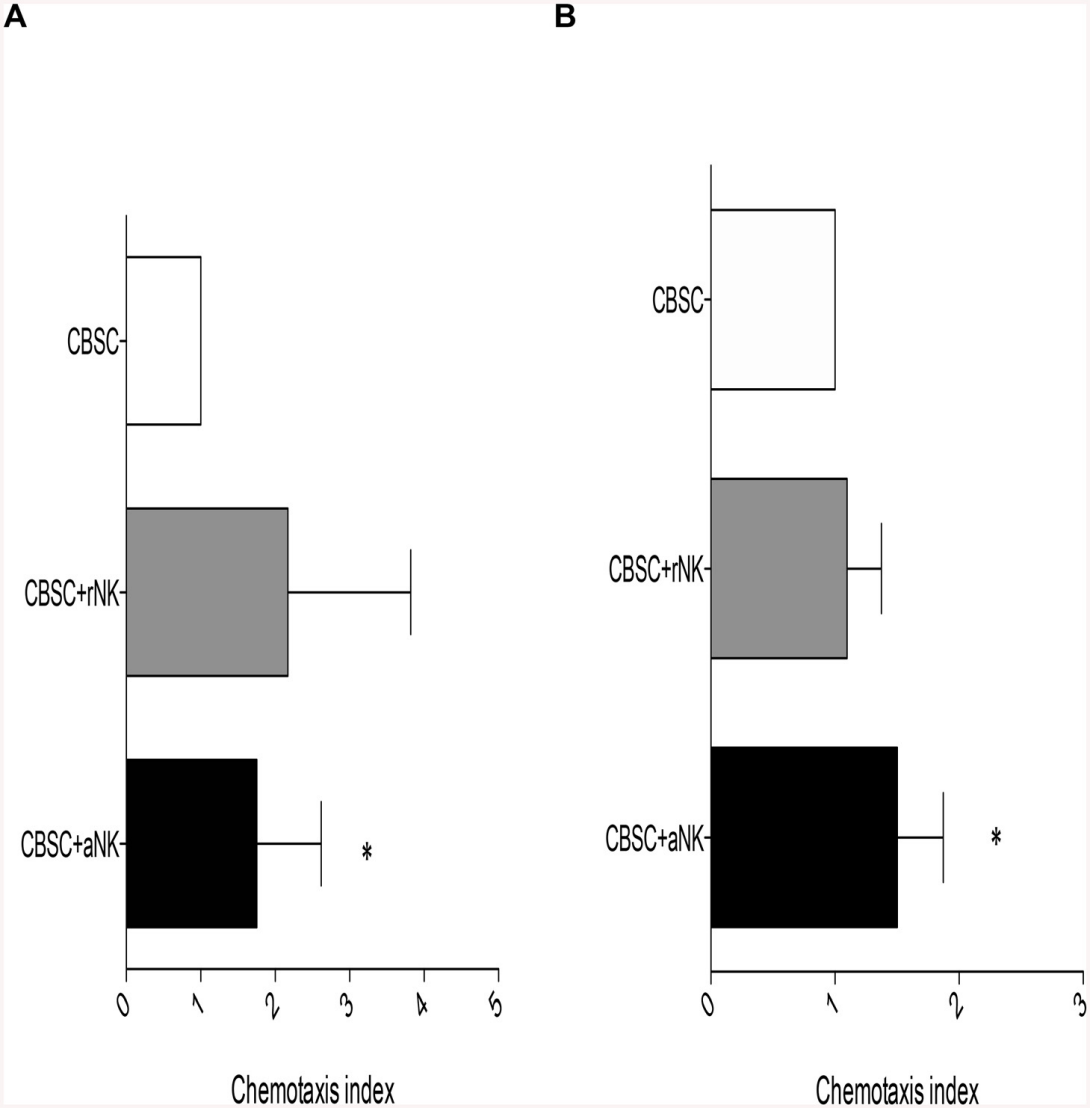
Activated CB NK Cells Enhance CBSC Migration Capacity *in vitro*. CBSC migration capacity was analyzed following 4 h transwell migration assays (N = 7). (A) Standard transwell migration assay using 125 ng/mL SDF-1α and 4 h incubation. (B) Suboptimal transwell migration assay using 50 ng/mL SDF-1α and 3 h incubation. * P < 0.05.

As an increased expression of CXCR4 and CXCR7 by CBSC was observed after co-culture with aNK cells, we studied whether this led to an increased response of CBSC to SDF-1α *in vitro*. Migration of CBSC *in vitro* towards SDF-1α was significantly enhanced in the presence of aNK cells when compared to CBSC alone (P < 0.05) (Fig 3A). Though an increased chemotactic index was observed in the presence of rNK cells, it was not significant when compared to CBSC alone. We then performed transwell migration assays using a sub-optimal dose of SDF-1α and a shorter incubation time. In line with the results obtained using a standard concentration of SDF-1α, aNK cells and not rNK cells specifically enhanced the migration capacity of CBSC towards a sub-optimal dose of SDF-1α (P < 0.05) (Fig 3B).

### Activated NK Cells Enhance the Short-Term and Long-Term Clonogenic Capacity of CBSC

**Fig 4:**
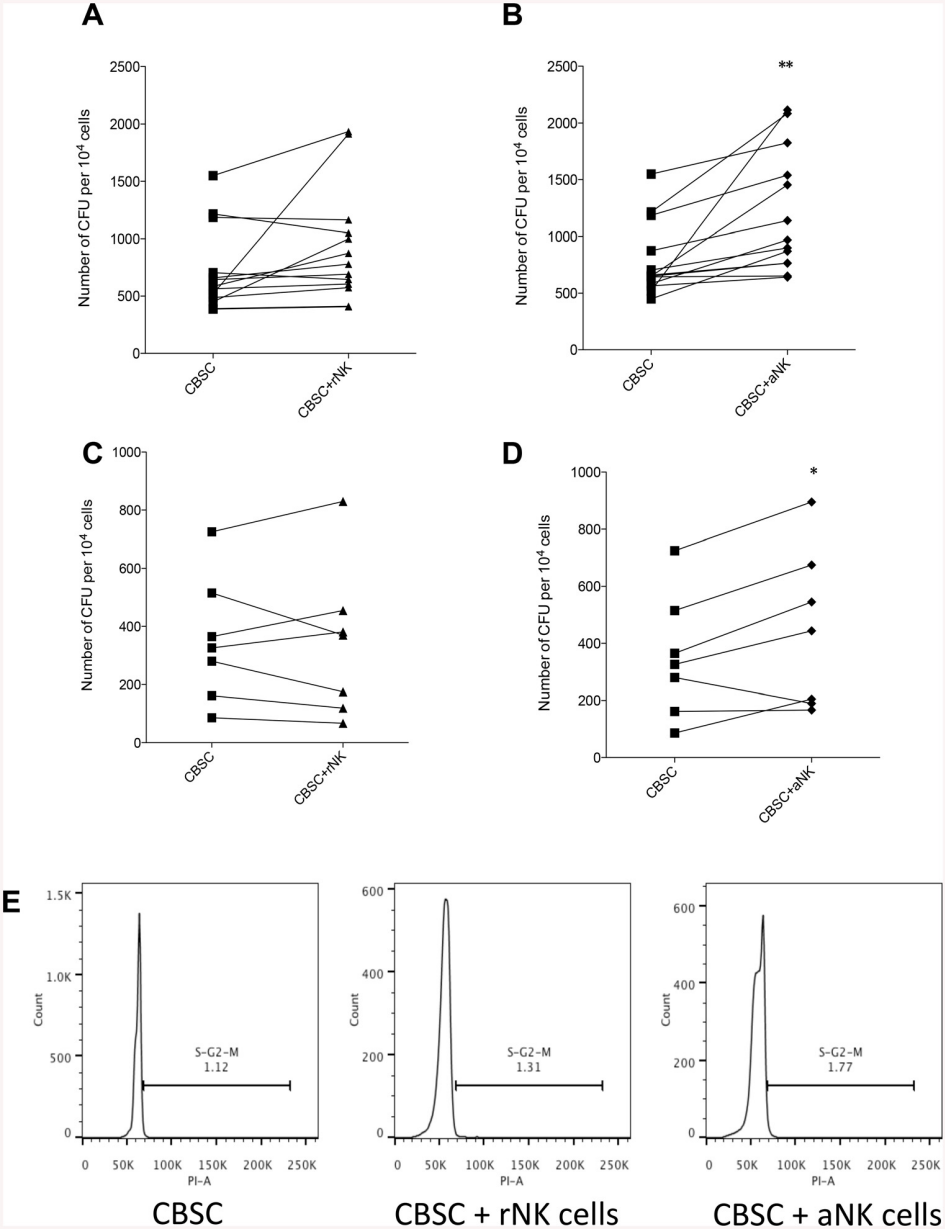
Activated NK Cells Enhance the Short-term and Long-term Clonogenic Capacity of CBSC. (A and B) Short-term CFU assays showing number of colonies formed after 4 h co-cultures of CBSC alone or with rNK cells (**A**) or aNK cells (**B**) (N = 13). (C and D) Long-term 4 week cobblestone cultures followed by CFU assays (LTC-IC) showing number of colonies formed after 4 h co-culture of CBSC alone or with rNK cells (**C**) or aNK cells (**D**) (N = 8). (**E**) Cell cycle analysis by flow cytometry of CBSC alone or after co-culture with rNK cells or aNK cells. * P < 0.05, ** P < 0.01.

The effect of CB NK cells on the short-term and long-term clonogenic capacity of CBSC was assessed using CFU assays and cobblestone cultures followed by long-term culture (LTC-IC) respectively. CBSC were cultured for 4 h in the presence of rNK cells or aNK cells prior to assessing their clonogenic capacity. A higher number of CFUs was obtained when CBSC were co-cultured with aNK cells (P < 0.01) (Fig 4B). Although the number of CFUs obtained from CBSC cultured with rNK cells was higher than the CBSC alone control this difference did not reach significance (Fig 4A). Co-culture of CBSC with aNK cells, but not with rNK cells, also increased the number of CFUs obtained from long-term LTC-IC cultures (P < 0.05) (Fig 4C and 4D respectively), suggesting that aNK cells enhance both the short and long-term clonogenic capacity of CBSC. Finally, we assessed whether NK cells could impact on the cell cycle of CBSC. However, we found that rNK or aNK cells had no effect on CBSC cell cycle (Fig 4E).

### Direct Contact between CBSC and Activated NK Cells Is Required to Enhance CBSC Clonogenic Capacity

**Fig 5:**
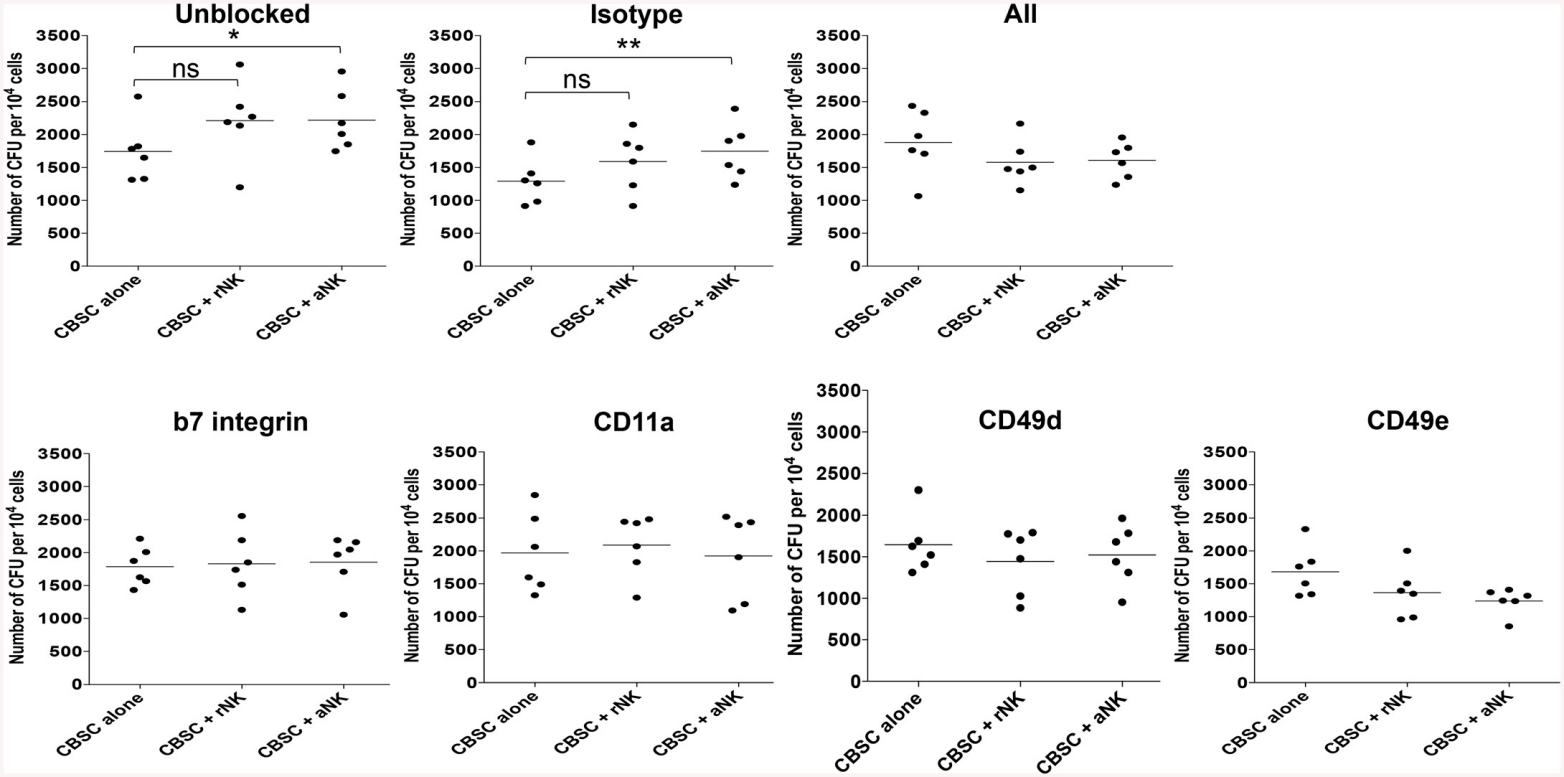
Blocking Integrin Receptors on CBSC Prevents Activated NK Cells from Enhancing CBSC Clonogenic Capacity. CFU assays showing number of colonies formed after 4 h co-cultures of CBSC alone or with rNK cells or aNK cells. CBSC were blocked for 1 h prior to co-culture with antibodies against beta-7 integrin, CD11a, CD49d, CD49e alone or in combination at 10 μg/mL, in comparison to isotype control (N = 6), * P < 0.05, ** P < 0.01.

The integrins beta-7, CD11a, CD49d and CD49e play key roles in HSC homing and maintenance within the BM niche [22–24]. Whilst it is unknown whether these integrins are important for the interaction between CBSC and NK cells, we observed regulation of the expression of these molecules by CBSC after co-culture with aNK cells. Therefore, we assessed whether blocking these integrins using antibodies would influence the effect of aNK cells on CBSC clonogenic capacity. Blocking beta-7 integrin, CD11a, CD49d or CD49e alone or in combination significantly reduced the ability of CB aNK cells to enhance CBSC clonogenic capacity (Fig 5), suggesting that direct contact between CBSC and NK cells via these specific integrins is required.

### Activated NK Cells Induce CXCL-9 Secretion by CBSC and Increase Their Clonogenicity

**Fig 6:**
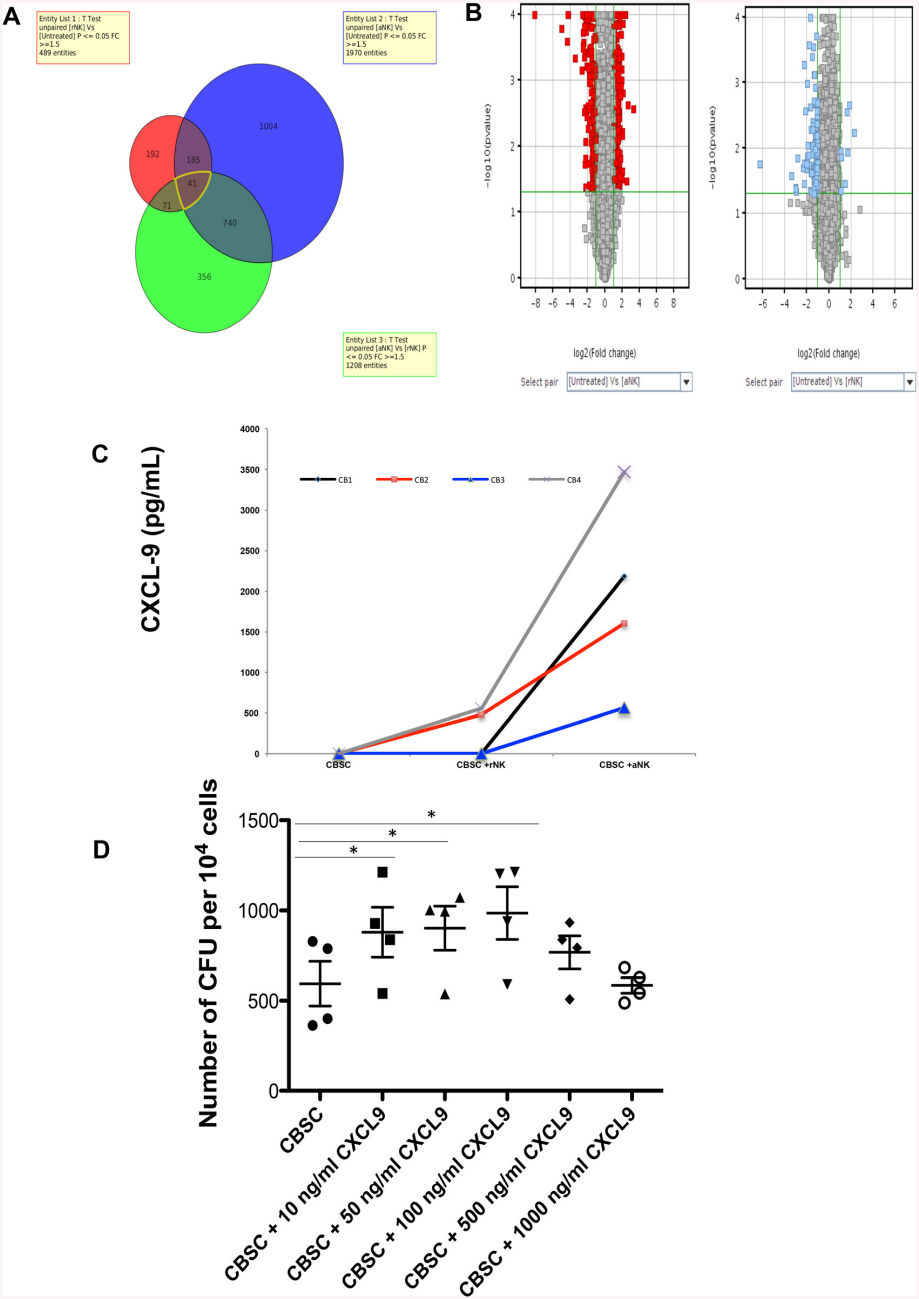
Changes in Gene and Protein Expression Induced in CBSC after Culture with Resting or Activated CB NK Cells. (**A and B**) CBSC were cultured for 4 h either alone or with resting or activated CB NK cells and then CBSC were re-isolated by cell sorting based on CD34 expression (N = 3). Gene expression profiles were compared by microarray analysis using GeneChip human Gene 2.0 ST Arrays and normalized. Data is presented as a Venn diagram and Volcano plots comparing the number of CBSC genes for which expression was changed (either up or down-regulated) by greater than 1.5 fold (p ≤ 0.05) after culture with resting or activated CB NK cells in comparison to CBSC alone (untreated). Refer to S1 Table for specific changes in gene expression. (**C**) Validation of microarray data showing increased CXCL-9 expression by CBSC after culture with activated CB NK cells. CXCL-9 protein was measured in culture supernatants using ELISA after co-culture of CBSC alone or with resting or activated CB NK cells (N = 4). (**D**) CFU assays showing number of colonies formed after 4 h culture of CBSC with different concentrations of CXCL9 as indicated (N = 4), * P < 0.05.

In order to identify molecular pathways that are modified in CBSC after culture with rNK cells or aNK cells, we performed gene expression analysis by microarray, comparing CBSC co-cultured with rNK cells or aNK cells. Clear transcriptional changes were observed when CBSC were cultured with NK cells (S1 Table). In particular, the expression of 489 genes was significantly changed at least 1.5 fold (p < 0.05) in CBSC after co-culture with rNK cells while the expression of 1970 genes was significantly modified when CBSC were cultured with aNK cells (Fig 6A and 6B). Notably, we found that one gene, CXCL-9, was particularly upregulated by CBSC after culture with aNK cells (274 fold-change (FC)). We confirmed that CBSC secreted high levels of CXCL-9 after co-culture with aNK cells but not with rNK cells (Fig 6C), suggesting that this chemokine might play a key role in regulating CBSC functions. We then assessed the effect of CXCL9 on CBSC functions by treating CBSC with different doses of recombinant CXCL9 followed by CFU assays. Treating CBSC with 10, 50 or 100 ng/ml of CXCL9 significantly increased CBSC clonogenicity (p < 0.05) while higher CXCL9 concentrations had no effect on CBSC function (Fig 6D).

### Activated NK Cells Secrete High Levels of IFN-γ and TNF-β

**Fig 7:**
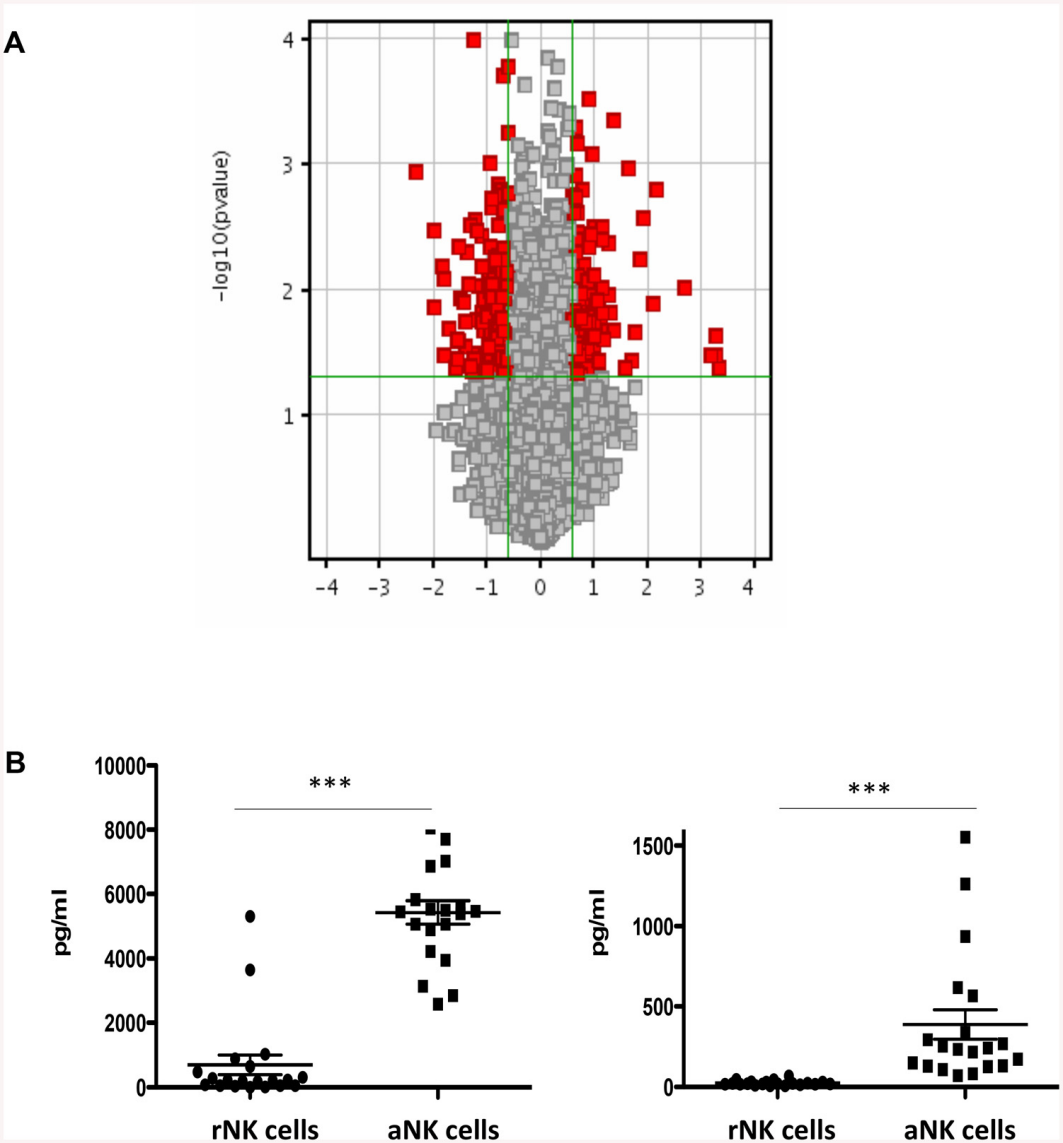
Changes in Gene and Protein Expression in NK Cells after Culture with IL-15. (**A**) Gene expression profiles were compared by microarray analysis using GeneChip human Gene 2.0 ST Arrays and normalized. Data is presented as a Volcano plot comparing the number of genes for which expression was changed (either up or down-regulated) by greater than 1.5 fold (p ≤ 0.05) between rNK cells and aNK cells. Refer to S2 Table for specific changes in gene expression. (**B**) IFN-γ and TNF-β secretion by NK cells was measured by ELISA following activation with IL-15 (N = 24) to confirm microarray data, *** P < 0.001.

To get a better understanding of the effects of rNK cells or aNK cells on CBSC, we performed a microarray analysis, comparing rNK cells to aNK cells. The expression of 148 genes was significantly upregulated at least 1.5 fold (p < 0.05), including IFN-γ and TNF-β, and 230 were significantly downregulated at least 1.5 fold (p < 0.05), including CXCR3 and CXCR4, in aNK cells compared to rNK cells (Fig 7A and S2 Table). Notably, we confirmed that following stimulation with IL-15 aNK cells secrete significantly more IFN-γ and TNF-β than rNK cells (Fig 7B).

## Discussion

Here we show that CB NK cells impact on CBSC function by modifying their homing receptor repertoire, improving their migration and clonogenic capacities *in vitro*, and by enhancing engraftment *in vivo*. We propose a model whereby events between co-transplanted cells influence the biological efficiency and engraftment of CBSC in the BM of humanized mice. However, we also show the considerable variability that can be observed between CB units and thus the relevance of the quality of the CB units used for CBT.

We explored the effect of accessory cells on CBSC engraftment *in vivo* using NSG mice. Notably, our results are in accordance with previous reports where it was shown that the addition of CD8+ T cells, but not CD4+ T cells, increased the migration capacity of CBSC *in vitro* and *in vivo* [13, 15, 16]. Higher levels of engraftment were observed in NSG mice transplanted with CBSC and rNK cells or aNK cells compared to CBSC alone. Variable levels of engraftment were observed between CB units; however, higher levels of engraftment were consistently observed in mice that received CBSC and rNK or aNK cells. The stimulus used to activate NK cells, IL-15, was selected based on the knowledge that this protein is produced in the BM and on current usage of IL-15 in clinical trials (http://clinicaltrials.gov). Nevertheless, the level of NK cell activation with IL-15 is variable and further research on the activation status of CB NK cells, notably prior IL-15 stimulation, is needed in order to better understand whether the extent of activation could potentially explain the variability observed in the levels of engraftment *in vivo*. It is possible that a longer incubation time with IL-15 could lead to a more consistent activation of CB NK cells. Although we observed variability amongst CB units in the results obtained *in vitro*, greater variability was observed for the *in vivo* experiments. The variability obtained in all *in vivo* experiments could potentially be greater than the variability between CB units in a clinical setting, as the CB units used in this work were research grade, thus with a lower quality standard when compared to clinical grade CB units. It will be important in the future to confirm that clinical grade CB units represent a more consistent quality of product.

The importance of the chemokine receptor CXCR4 in HSC homing to the BM is well documented [22–24, 31, 37, 38]. Recent reports correlate CBSC migration *in vitro* with engraftment in humans, providing further support for the model that HSC migration is a critical step in the successful establishment of hematopoiesis [39]. We showed that the expression of homing receptors by CBSC was modified in the presence of aNK but not rNK cells. Of particular interest was the higher expression of CXCR4 observed by CBSC in the presence of aNK cells. Moreover, the migration capacity of CBSC assessed by *in vitro* transwell migration assay was also improved in the presence of aNK cells. For these assays we used a standard SDF-1α concentration and a suboptimal concentration to assess whether aNK cells had a synergic effect on CBSC migration. A higher migration index was observed when using CBSC co-cultured with aNK cells. It is possible that the positive effect observed on CBSC migration in the presence of aNK cells is secondary to the higher levels of CXCR4 expression.

A higher number of CFU was observed for CBSC in the presence of aNK cells from either short-term or long-term cultures suggesting that aNK cells not only modify CBSC trafficking capacity but also their clonogenicity. Notably, for all the *in vitro* assay data presented here a 4 h co-culture between CBSC and CB NK cells was used, however 24 h co-cultures were also performed yielding the same results. We recognize that the CBSC:NK cells ratio (1:5) used for all *in vitro* experiments may or may not reflect the physiologic context. However, we chose this ratio to ensure that it would not overwhelm the *in vitro* models used.

We attempted to assess whether the effects of aNK cells on CBSC was mediated via soluble factors or was cell contact dependent by performing CFU assays using transwells, but we were unable to pursue this approach as the transwell plates that had to be used to address this question impacted negatively on the interaction of NK cells with CBSC (data not shown). However, the assays performed using blocking antibodies against beta-7 integrin, CD11a, CD49d and CD49e suggest that cell-cell interaction between CBSC and NK cells via these molecules is required for the latter to have an effect on the clonogenic capacity of CBSC.

Finally, we showed by gene expression profiling analysis and ELISA that CBSC secreted high levels of CXCL9 after co-culture with aNK cells. Moreover, the microarray analysis also revealed that CBSC expressed higher messenger levels of genes that are regulated by IFN-γ and that aNK cells secreted significantly more IFN-γ and TNF-β than rNK cells. It could be that the CB NK cells secrete IFN-γ, which in turn induced CXCL9 expression by CBSC, acting on their clonogenicity as we showed that recombinant CXCL9 increased CBSC clonogenicity. The effects of CXCL9 on CBSC phenotype and functions need further investigation as little is known of the impact of this chemokine on HSC.

The data presented here demonstrate that CB NK cells can impact on the homing and clonogenicity of CBSC. We therefore propose that CB NK cells are a facilitator of CBSC engraftment *in vivo*. These results should lead to further research in order to gain a better understanding of the interactions between CBSC and accessory cells and their use in the clinic. In conclusion, these results give the basis for a novel approach in the area of CBT whereby NK cell therapy or the manipulation of the composition of the cellular content of a CB unit by using cytokines such as IL-15, could be used to improve CBSC engraftment and CBT outcomes.

## Supporting Information

S1 Table List of Genes Differentially Regulated in CBSC after Culture with rNK or aNK cells. (DOCX)

S2 Table List of Genes Differentially Regulated in NK Cells after Activation with IL-15. (DOCX)
