# Supplementary material for: Natural Killer Cells Improve Hematopoietic Stem Cell Engraftment by Increasing Stem Cell Clonogenicity In Vitro and in a Humanized Mouse Model
Source: PLoS One. 2015 Oct 14;10(10):e0138623. doi: 10.1371/journal.pone.0138623 (PMC4605799; doi:10.1371/journal.pone.0138623)
Supplement: S1 Table — (DOCX) [file pone.0138623.s001.docx]

**aNK versus untreated FC1.5 p<0.05**

| **Transcript Cluster Id** | **p value** | **FC (abs)** | **Regulation** | **Gene Description** |
| --- | --- | --- | --- | --- |
| 16977045 | 2.23E-08 | 274.25494 | up | chemokine(C-X-Cmotif)ligand9 |
| 17068317 | 1.68E-04 | 31.957722 | up |  |
| 16885116 | 2.59E-04 | 20.883348 | up |  |
| 16977052 | 1.06E-05 | 19.201567 | up | chemokine(C-X-Cmotif)ligand10 |
| 16689332 | 3.43E-05 | 18.997446 | up | guanylatebindingprotein1,interferon-inducible |
| 17016692 | 4.74E-04 | 10.943398 | up | ubiquitinD |
| 17068296 | 7.17E-04 | 6.4127164 | up | indoleamine2,3-dioxygenase1 |
| 16931766 | 5.49E-05 | 6.0355225 | up | kelchdomaincontaining7B |
| 16906534 | 1.33E-05 | 5.9405017 | up | signaltransducerandactivatoroftranscription1,91kDa |
| 16796694 | 1.01E-04 | 5.935695 | up | tryptophanyl-tRNAsynthetase |
| 17117752 | 3.30E-04 | 5.8042336 | up |  |
| 16813029 | 1.91E-04 | 5.6961718 | up | hyaluronanandproteoglycanlinkprotein3 |
| 17051159 | 2.93E-04 | 5.6111093 | up | hypoxiainduciblelipiddroplet-associated |
| 16830461 | 2.72E-04 | 5.3494186 | up | fibroblastgrowthfactor11 |
| 16999776 | 6.53E-05 | 5.2697916 | up | interferonregulatoryfactor1 |
| 16726880 | 4.58E-04 | 5.211663 | up |  |
| 16931763 | 1.56E-04 | 5.2106085 | up |  |
| 16936624 | 1.05E-04 | 5.1973877 | up | outerdensefiberofspermtails3B |
| 16977058 | 5.94E-06 | 5.1076703 | up | chemokine(C-X-Cmotif)ligand11 |
| 16666959 | 4.61E-05 | 5.0960884 | up | guanylatebindingprotein1,interferon-induciblepseudogene1 |
| 16836624 | 0.002987491 | 5.0934663 | up | microRNA21 |
| 16990203 | 0.0190199 | 4.907965 | up | vaultRNA1-3 |
| 16675301 | 3.86E-04 | 4.879109 | up | regulatorofG-proteinsignaling1 |
| 17093536 | 0.015245923 | 4.811929 | up | chemokine(C-Cmotif)ligand19 |
| 16925589 | 0.008541574 | 4.8058743 | up |  |
| 16666485 | 0.002633728 | 4.7981186 | up | interferon-inducedprotein44-like |
| 16707184 | 1.26E-04 | 4.78231 | up | interferon-inducedproteinwithtetratricopeptiderepeats3 |
| 16788420 | 1.43E-05 | 4.7256017 | up |  |
| 16767247 | 0.042793803 | 4.5920606 | up | interferon,gamma |
| 16788417 | 2.43E-05 | 4.5708175 | up |  |
| 16766130 | 1.50E-04 | 4.46864 | up | RNA,U7smallnuclear40pseudogene |
| 17039977 | 2.05E-05 | 4.418084 | up | transporter1,ATP-bindingcassette,sub-familyB(MDR/TAP) |
| 16719644 | 1.82E-04 | 4.4135575 | up | BCL2/adenovirusE1B19kDainteractingprotein3\|BCL2/adenovirusE1B19kDainteractingprotein3pseudogene1 |
| 17017979 | 1.99E-05 | 4.410439 | up | transporter1,ATP-bindingcassette,sub-familyB(MDR/TAP) |
| 17034791 | 1.84E-05 | 4.4012194 | up | transporter1,ATP-bindingcassette,sub-familyB(MDR/TAP) |
| 17027144 | 1.66E-05 | 4.394308 | up | transporter1,ATP-bindingcassette,sub-familyB(MDR/TAP) |
| 17032476 | 1.82E-05 | 4.394074 | up | transporter1,ATP-bindingcassette,sub-familyB(MDR/TAP) |
| 17037271 | 1.63E-05 | 4.3891554 | up | transporter1,ATP-bindingcassette,sub-familyB(MDR/TAP) |
| 17029788 | 1.71E-05 | 4.3844285 | up | transporter1,ATP-bindingcassette,sub-familyB(MDR/TAP) |
| 16818973 | 0.002862233 | 4.267809 | up |  |
| 16689352 | 6.11E-04 | 4.266642 | up | guanylatebindingprotein2,interferon-inducible |
| 17063977 | 0.003640008 | 4.1858077 | up | familywithsequencesimilarity115,memberCpseudogene |
| 17042487 | 8.63E-06 | 4.172201 | up | transporter1,ATP-bindingcassette,sub-familyB(MDR/TAP) |
| 16689354 | 3.97E-04 | 4.025793 | up | guanylatebindingprotein2,interferon-inducible |
| 16761201 | 0.005428075 | 3.952028 | up |  |
| 16720085 | 0.002431346 | 3.938992 | up | interferoninducedtransmembraneprotein1 |
| 16698782 | 1.50E-05 | 3.8294086 | up | microRNA29c |
| 16842659 | 1.17E-04 | 3.6971056 | up | aldolaseC,fructose-bisphosphate |
| 16965313 | 6.52E-04 | 3.6634407 | up | leucineaminopeptidase3 |
| 16981516 | 0.002734229 | 3.5664334 | up |  |
| 17016360 | 0.039078537 | 3.5662174 | up | histonecluster1,H4b\|histonecluster2,H4b\|histonecluster4,H4\|histonecluster2,H4a\|histonecluster1,H4l\|histonecluster1,H4e\|histonecluster1,H4h\|histonecluster1,H4c\|histonecluster1,H4j\|histonecluster1,H4k\|histonecluster1,H4f\|histonecluster1,H4d\|histonecluster1,H4a\|histonecluster1,H4i |
| 16921827 | 7.89E-08 | 3.5621486 | up | microRNA155\|MIR155hostgene(non-proteincoding) |
| 16793674 | 3.50E-04 | 3.5538116 | up |  |
| 16778559 | 7.99E-04 | 3.5245748 | up | epithelialstromalinteraction1(breast) |
| 16889829 | 0.018381204 | 3.5128438 | up | inducibleT-cellco-stimulator |
| 16884918 | 0.001062189 | 3.4925601 | up | insulininducedgene2 |
| 17067771 | 0.004256584 | 3.4369464 | up | smallnucleolarRNA,C/Dbox13 |
| 16689400 | 8.04E-04 | 3.3960176 | up | guanylatebindingprotein5 |
| 16859763 | 8.65E-04 | 3.3137658 | up | interferon,gamma-inducibleprotein30 |
| 16758072 | 0.004025209 | 3.3097718 | up |  |
| 16922606 | 3.31E-04 | 3.2818346 | up |  |
| 16905123 | 0.002199171 | 3.2439787 | up |  |
| 16666940 | 1.52E-04 | 3.2413573 | up |  |
| 16758052 | 0.001117058 | 3.2358997 | up | purinergicreceptorP2X,ligand-gatedionchannel,7 |
| 17062945 | 0.00107367 | 3.2013192 | up |  |
| 16757324 | 0.0024308 | 3.165976 | up | 2'-5'-oligoadenylatesynthetase1,40/46kDa |
| 16843511 | 8.31E-05 | 3.1341887 | up | chemokine(C-Cmotif)ligand5 |
| 16944695 | 3.18E-04 | 3.0389993 | up | poly(ADP-ribose)polymerasefamily,member14 |
| 16912360 | 0.042490903 | 3.012896 | up |  |
| 16691730 | 0.002616389 | 3.005175 | up | FcfragmentofIgG,highaffinityIb,receptor(CD64)\|FcfragmentofIgG,highaffinityIc,receptor(CD64),pseudogene |
| 16958124 | 0.001533764 | 3.0012364 | up | poly(ADP-ribose)polymerasefamily,member9 |
| 17016516 | 0.008827283 | 2.9729826 | up | RNA,U7smallnuclear26pseudogene |
| 16727626 | 0.006456422 | 2.9631135 | up | RNA,U7smallnuclear23pseudogene |
| 16715361 | 1.60E-04 | 2.942266 | up | prolyl4-hydroxylase,alphapolypeptideI |
| 16821621 | 0.015939564 | 2.9415596 | up | interferonregulatoryfactor8 |
| 17058719 | 0.022826042 | 2.9362273 | up | neutrophilcytosolicfactor1Cpseudogene |
| 16923448 | 0.008025887 | 2.931836 | up | 1-acylglycerol-3-phosphateO-acyltransferase3 |
| 16850216 | 8.86E-05 | 2.901268 | up | secretedandtransmembrane1 |
| 16929615 | 6.05E-04 | 2.8821652 | up | apolipoproteinL,6 |
| 17081401 | 3.63E-04 | 2.8732307 | up | N-mycdownstreamregulated1 |
| 16692834 | 4.57E-04 | 2.8729415 | up | cathepsinS |
| 16661646 | 0.028557455 | 2.7863672 | up | RNA,U11smallnuclear |
| 16724861 | 2.37E-05 | 2.7816813 | up | serpinpeptidaseinhibitor,cladeG(C1inhibitor),member1 |
| 17052864 | 0.013612726 | 2.774506 | up | familywithsequencesimilarity115,memberC\|familywithsequencesimilarity115,memberCpseudogene\|familywithsequencesimilarity115,memberD(pseudogene) |
| 16794955 | 8.27E-04 | 2.7721782 | up |  |
| 16782687 | 0.003170827 | 2.758262 | up | interferonregulatoryfactor9 |
| 17033637 | 0.038124695 | 2.719476 | up |  |
| 17063973 | 0.042854957 | 2.7095842 | up |  |
| 16967368 | 0.010947381 | 2.6982388 | up | UDPglucuronosyltransferase2family,polypeptideB28 |
| 16662584 | 0.002071385 | 2.6604655 | up | SH3domaincontaining21\|uncharacterizedLOC100127947 |
| 16823866 | 0.00734935 | 2.659572 | up | suppressorofcytokinesignaling1 |
| 16936640 | 0.02011627 | 2.6559541 | up |  |
| 16702226 | 0.029125819 | 2.648518 | up |  |
| 16666509 | 0.014243015 | 2.6402023 | up | interferon-inducedprotein44 |
| 17117682 | 0.006586002 | 2.6379693 | up | uncharacterizedLOC100507419\|uncharacterizedLOC100653076 |
| 16856803 | 0.008092733 | 2.6334386 | up | growtharrestandDNA-damage-inducible,beta |
| 16860709 | 5.68E-04 | 2.6311226 | up | glucose-6-phosphateisomerase |
| 16760868 | 0.001321097 | 2.6122606 | up | solutecarrierfamily2(facilitatedglucosetransporter),member3 |
| 17059776 | 0.0039971 | 2.6025198 | up | sterilealphamotifdomaincontaining9-like |
| 16659545 | 6.57E-04 | 2.5884137 | up | forkhead-associated(FHA)phosphopeptidebindingdomain1 |
| 16901197 | 0.015125778 | 2.5566635 | up | smallnucleolarRNA,C/Dbox89 |
| 16794145 | 0.02590113 | 2.5436444 | up |  |
| 16707180 | 0.006149504 | 2.5416183 | up | interferon-inducedproteinwithtetratricopeptiderepeats2 |
| 16830202 | 0.008854865 | 2.516613 | up | XIAPassociatedfactor1 |
| 16726886 | 0.004695288 | 2.5146275 | up |  |
| 17046911 | 0.00169276 | 2.5106628 | up | neutrophilcytosolicfactor1Bpseudogene |
| 16800680 | 0.015466291 | 2.5091543 | up | sulfidequinonereductase-like(yeast) |
| 16721280 | 0.002851319 | 2.5085838 | up | tripartitemotifcontaining22 |
| 17083357 | 0.010434439 | 2.497783 | up |  |
| 16757373 | 0.039689574 | 2.4965684 | up | 2'-5'-oligoadenylatesynthetase2,69/71kDa |
| 16702215 | 0.002223934 | 2.4962516 | up |  |
| 16936643 | 0.003190514 | 2.489774 | up | synaptonemalcomplexcentralelementprotein3 |
| 16969839 | 1.12E-04 | 2.478956 | up | alpha-kinase1 |
| 17005655 | 0.001665682 | 2.4753492 | up | butyrophilin,subfamily3,memberA1 |
| 16943336 | 0.021773675 | 2.4700618 | up | transmembraneprotein45A |
| 17102129 | 2.46E-04 | 2.4640422 | up | spermidine/spermineN1-acetyltransferase1 |
| 16956448 | 0.005557745 | 2.4639153 | up | glucan(1,4-alpha-),branchingenzyme1 |
| 17031373 | 4.35E-05 | 2.4631417 | up | ubiquitinD\|gamma-aminobutyricacid(GABA)Breceptor,1 |
| 16770471 | 0.006956694 | 2.4627764 | up |  |
| 16887736 | 8.41E-04 | 2.4484637 | up | pyruvatedehydrogenasekinase,isozyme1 |
| 16681989 | 0.003377956 | 2.4448292 | up |  |
| 16885189 | 0.01779044 | 2.4388075 | up | RNA,U4atacsmallnuclear(U12-dependentsplicing) |
| 16739606 | 0.00295773 | 2.4376874 | up |  |
| 16944654 | 0.004099008 | 2.4333394 | up | familywithsequencesimilarity162,memberA |
| 16796749 | 0.001471771 | 2.4074109 | up |  |
| 16902250 | 0.005511231 | 2.4050457 | up |  |
| 16766093 | 0.012277221 | 2.3986895 | up | signaltransducerandactivatoroftranscription2,113kDa |
| 16974686 | 0.025160259 | 2.3708165 | up |  |
| 16684142 | 0.011186434 | 2.369323 | up | RNA,U7smallnuclear29pseudogene\|RNA,U7smallnuclear45pseudogene |
| 16873329 | 0.00146611 | 2.3657312 | up | FBJmurineosteosarcomaviraloncogenehomologB |
| 16998044 | 0.00824606 | 2.35932 | up |  |
| 16726081 | 8.65E-04 | 2.3529174 | up | retinoicacidreceptorresponder(tazaroteneinduced)3 |
| 16999773 | 0.010716829 | 2.3314288 | up |  |
| 17063975 | 0.007634111 | 2.3165426 | up | CTAGEfamily,member6,pseudogene\|CTAGEfamily,member15,pseudogene |
| 16784278 | 0.033370163 | 2.302418 | up |  |
| 17018615 | 2.18E-04 | 2.2916043 | up | etsvariant7 |
| 17117999 | 0.022136917 | 2.268856 | up |  |
| 16889455 | 3.04E-05 | 2.2653086 | up | caspase10,apoptosis-relatedcysteinepeptidase |
| 16804490 | 6.24E-04 | 2.2605104 | up | interferonstimulatedexonucleasegene20kDa |
| 16836311 | 9.01E-04 | 2.2553835 | up | noggin |
| 16842237 | 0.03870222 | 2.2520664 | up | smallnucleolarRNA,C/Dbox3C |
| 16953121 | 0.022598883 | 2.2499344 | up | smallnucleolarRNA,C/Dbox13pseudogene3 |
| 16714545 | 0.002558076 | 2.2375994 | up |  |
| 16998848 | 0.012181325 | 2.226035 | up |  |
| 16993653 | 0.04211037 | 2.2230968 | up |  |
| 16891254 | 0.006827432 | 2.2172863 | up |  |
| 16733985 | 0.00735663 | 2.2082818 | up | MIR210hostgene(non-proteincoding) |
| 16762565 | 0.03427891 | 2.1983476 | up |  |
| 17100810 | 0.003023719 | 2.1862159 | up |  |
| 16835313 | 1.62E-04 | 2.1788812 | up | T-box21 |
| 16889450 | 0.00999987 | 2.177679 | up | RNA,U7smallnuclear45pseudogene\|RNA,U7smallnuclear29pseudogene |
| 17011939 | 0.002664064 | 2.173398 | up | familywithsequencesimilarity26,memberF |
| 16924518 | 0.008086497 | 2.1605437 | up |  |
| 16944665 | 3.32E-05 | 2.1599238 | up | deltex3-like(Drosophila) |
| 16970971 | 0.003915228 | 2.1528075 | up | interleukin15 |
| 16905115 | 0.007221974 | 2.1490667 | up |  |
| 16846915 | 0.005058774 | 2.1480756 | up | tripartitemotifcontaining25\|microRNA3614 |
| 16959925 | 0.004166811 | 2.1477864 | up | 5'-3'exoribonuclease1 |
| 17115039 | 6.72E-04 | 2.1476934 | up | CSAGfamily,member2\|CSAGfamily,member3 |
| 17107896 | 6.76E-04 | 2.1438868 | up | CSAGfamily,member2\|CSAGfamily,member3 |
| 16776339 | 0.016168963 | 2.142725 | up | tumornecrosisfactor(ligand)superfamily,member13b |
| 17118158 | 0.007037897 | 2.1408737 | up |  |
| 16801835 | 0.017181138 | 2.1324108 | up | lactamase,beta |
| 16906063 | 0.007545549 | 2.1304529 | up |  |
| 17091901 | 0.03984966 | 2.1304126 | up |  |
| 16679897 | 0.04102919 | 2.1302617 | up |  |
| 16866392 | 0.04108202 | 2.1301198 | up |  |
| 16979334 | 0.040110756 | 2.1296165 | up |  |
| 16950181 | 0.039900336 | 2.1294565 | up |  |
| 16888910 | 0.04157303 | 2.1259043 | up |  |
| 16733806 | 0.040773544 | 2.123429 | up |  |
| 16822254 | 0.04045606 | 2.1233113 | up |  |
| 16704088 | 0.040600047 | 2.122216 | up |  |
| 16699572 | 0.04044106 | 2.1195362 | up |  |
| 16872223 | 0.001461691 | 2.1121874 | up |  |
| 16801600 | 0.001524381 | 2.0981686 | up |  |
| 17035371 | 0.005079502 | 2.0959265 | up | HLAcomplexP5(non-proteincoding) |
| 17056787 | 0.03791564 | 2.0884044 | up |  |
| 16936607 | 2.34E-04 | 2.0854468 | up | SCOcytochromeoxidasedeficienthomolog2(yeast)\|thymidinephosphorylase |
| 16819355 | 0.001142284 | 2.0806446 | up | NLRfamily,CARDdomaincontaining5 |
| 16934476 | 0.001919483 | 2.0765681 | up | apolipoproteinL,2 |
| 16793176 | 0.025456434 | 2.075487 | up | GTPcyclohydrolase1 |
| 16757225 | 9.82E-04 | 2.0715616 | up | TRAF-typezincfingerdomaincontaining1 |
| 16823275 | 0.004661059 | 2.0676696 | up | uncharacterizedLOC100653076\|uncharacterizedLOC100507419 |
| 16994693 | 0.01787581 | 2.0644798 | up |  |
| 16847199 | 0.036019597 | 2.0574 | up |  |
| 16989736 | 0.00630932 | 2.048518 | up | earlygrowthresponse1 |
| 17118246 | 0.007914957 | 2.0478835 | up | majorhistocompatibilitycomplex,classI,E\|uncharacterizedLOC100653072 |
| 16764220 | 0.00282646 | 2.0478704 | up | Rashomologenrichedinbrainlike1 |
| 17118232 | 0.007876478 | 2.0474405 | up | majorhistocompatibilitycomplex,classI,E\|uncharacterizedLOC100653072 |
| 17082106 | 0.034648925 | 2.0472395 | up |  |
| 17118242 | 0.007877774 | 2.0463078 | up | majorhistocompatibilitycomplex,classI,E\|uncharacterizedLOC100653072 |
| 17118228 | 0.007847281 | 2.046151 | up | majorhistocompatibilitycomplex,classI,E\|uncharacterizedLOC100653072 |
| 16953424 | 0.013090409 | 2.0426521 | up | 6-phosphofructo-2-kinase/fructose-2,6-biphosphatase4 |
| 16818431 | 2.18E-04 | 2.0299642 | up | zincfingerprotein267 |
| 17031434 | 0.015661864 | 2.0276618 | up | interferoninducedtransmembraneprotein4pseudogene |
| 17000520 | 0.020089308 | 2.02657 | up | smallnucleolarRNA,C/Dbox63 |
| 16876764 | 0.004578861 | 2.016428 | up | radicalS-adenosylmethioninedomaincontaining2 |
| 16913075 | 0.047574244 | 2.0043669 | up | RNA,5Sribosomal483 |
| 17117736 | 0.014720788 | 2.001233 | up |  |
| 16715133 | 0.04686403 | 1.99731 | up | pyrophosphatase(inorganic)1 |
| 16721307 | 0.02275134 | 1.9846647 | up | olfactoryreceptor,family52,subfamilyN,member4 |
| 17005685 | 0.01492703 | 1.9845048 | up | butyrophilin,subfamily3,memberA3 |
| 16707202 | 0.001412109 | 1.9803247 | up | interferon-inducedproteinwithtetratricopeptiderepeats5 |
| 16708878 | 0.023319935 | 1.9766114 | up |  |
| 16882285 | 5.36E-05 | 1.9749633 | up | vesicle-associatedmembraneprotein5(myobrevin) |
| 17007962 | 0.03357079 | 1.9742059 | up |  |
| 16838581 | 0.002724689 | 1.9741096 | up | ringfingerprotein213 |
| 16676086 | 0.001271725 | 1.9720541 | up | proteintyrosinephosphatase,receptortype,fpolypeptide(PTPRF),interactingprotein(liprin),alpha4 |
| 16711484 | 0.008458186 | 1.9679893 | up | interleukin15receptor,alpha |
| 17030288 | 0.009171058 | 1.9676143 | up | majorhistocompatibilitycomplex,classI,E |
| 17017935 | 0.005798061 | 1.9654309 | up | majorhistocompatibilitycomplex,classII,DObeta\|transporter2,ATP-bindingcassette,sub-familyB(MDR/TAP) |
| 17037774 | 0.00975962 | 1.9647454 | up | majorhistocompatibilitycomplex,classI,E |
| 17100651 | 0.002957018 | 1.9646212 | up |  |
| 17027443 | 0.008979376 | 1.9644501 | up | majorhistocompatibilitycomplex,classI,E |
| 17032993 | 0.013495074 | 1.9637084 | up | majorhistocompatibilitycomplex,classI,E |
| 17040488 | 0.009077726 | 1.9614229 | up | majorhistocompatibilitycomplex,classI,E |
| 17035115 | 0.013372673 | 1.9605604 | up | majorhistocompatibilitycomplex,classI,E |
| 17006261 | 0.013333735 | 1.95946 | up | majorhistocompatibilitycomplex,classI,E |
| 16832031 | 0.016646821 | 1.9573425 | up | smallnucleolarRNA,C/Dbox3A\|smallnucleolarRNA,C/Dbox3B-1\|smallnucleolarRNA,C/Dbox3B-2\|smallnucleolarRNA,C/Dbox3D\|smallnucleolarRNA,C/Dbox3C |
| 17105047 | 6.70E-04 | 1.9549012 | up | phosphoglyceratekinase1\|uncharacterizedLOC100653302\|uncharacterizedLOC100652805 |
| 17033890 | 0.013817636 | 1.9547575 | up | interferoninducedtransmembraneprotein4pseudogene |
| 17038932 | 0.014022186 | 1.9526347 | up | interferoninducedtransmembraneprotein4pseudogene |
| 16832005 | 0.016083654 | 1.9498855 | up | smallnucleolarRNA,C/Dbox3B-1\|smallnucleolarRNA,C/Dbox3B-2\|smallnucleolarRNA,C/Dbox3A\|smallnucleolarRNA,C/Dbox3D\|smallnucleolarRNA,C/Dbox3C |
| 16842215 | 0.01604317 | 1.9485326 | up |  |
| 16674927 | 0.003833028 | 1.9464189 | up | ralguaninenucleotidedissociationstimulator-like1 |
| 17007307 | 2.90E-05 | 1.9447289 | up | proteasome(prosome,macropain)subunit,betatype,9(largemultifunctionalpeptidase2) |
| 16665674 | 5.10E-05 | 1.9411649 | up | adenylatekinase4\|adenylatekinase4pseudogene3\|adenylatekinaseisoenzyme4,mitochondrial-like |
| 17117443 | 0.01005802 | 1.9360584 | up |  |
| 16834091 | 0.023704475 | 1.9304457 | up | insulin-likegrowthfactorbindingprotein4 |
| 16747570 | 0.013978054 | 1.9304192 | up | enolase2(gamma,neuronal) |
| 16761212 | 0.02188406 | 1.9259993 | up | C-typelectindomainfamily2,memberB |
| 16827483 | 7.80E-04 | 1.9234589 | up | proteasome(prosome,macropain)subunit,betatype,10 |
| 16942038 | 0.016337624 | 1.923435 | up |  |
| 17022949 | 0.036715854 | 1.9190613 | up |  |
| 16965268 | 1.81E-04 | 1.9166883 | up |  |
| 17028360 | 3.09E-05 | 1.9162859 | up | proteasome(prosome,macropain)subunit,betatype,9(largemultifunctionalpeptidase2) |
| 16861997 | 0.0106109 | 1.9158312 | up | zincfingerprotein36,C3Htype,homolog(mouse) |
| 17051950 | 0.008527724 | 1.9129242 | up |  |
| 17038644 | 2.14E-05 | 1.9056033 | up | proteasome(prosome,macropain)subunit,betatype,9(largemultifunctionalpeptidase2) |
| 17033661 | 1.96E-05 | 1.9050723 | up | proteasome(prosome,macropain)subunit,betatype,9(largemultifunctionalpeptidase2) |
| 16687875 | 0.038266685 | 1.9049711 | up | junproto-oncogene |
| 16891804 | 0.002076552 | 1.9038681 | up |  |
| 16691721 | 0.03298045 | 1.9030527 | up | uncharacterizedLOC100132966\|uncharacterizedLOC100505630\|SLIT-ROBORhoGTPaseactivatingprotein2B(pseudogene) |
| 16777996 | 0.01924134 | 1.8955616 | up |  |
| 17042403 | 0.041154366 | 1.8901902 | up |  |
| 17118287 | 0.034536306 | 1.8856664 | up |  |
| 17098626 | 0.003654679 | 1.884098 | up | adenylatekinase1 |
| 16849576 | 0.01134709 | 1.8831719 | up | lectin,galactoside-binding,soluble,3bindingprotein |
| 16832319 | 0.020235166 | 1.8821208 | up | WDrepeatandSOCSboxcontaining1 |
| 16909413 | 0.006505519 | 1.8819644 | up |  |
| 16734762 | 6.33E-04 | 1.8767719 | up | tripartitemotifcontaining21 |
| 16849400 | 0.006336255 | 1.8763351 | up | suppressorofcytokinesignaling3 |
| 16998039 | 0.018430693 | 1.8739308 | up |  |
| 17042443 | 0.00983161 | 1.871388 | up | transporter2,ATP-bindingcassette,sub-familyB(MDR/TAP) |
| 16908251 | 0.003550067 | 1.8702419 | up | tensin1 |
| 16815852 | 0.026982369 | 1.8659409 | up |  |
| 16850607 | 0.033267852 | 1.8634641 | up | RNA,U7smallnuclear25pseudogene\|RNA,U7smallnuclear11pseudogene |
| 16966162 | 0.033631273 | 1.8599513 | up | RNA,U7smallnuclear25pseudogene\|RNA,U7smallnuclear11pseudogene |
| 16793803 | 0.035662126 | 1.8560601 | up |  |
| 17054325 | 0.0044495 | 1.8525611 | up |  |
| 16834545 | 8.18E-04 | 1.8499205 | up | interferon-inducedprotein35 |
| 17005797 | 0.008296558 | 1.8497994 | up | microRNA3143 |
| 16791444 | 0.035521317 | 1.849396 | up | granzymeB(granzyme2,cytotoxicT-lymphocyte-associatedserineesterase1) |
| 16716341 | 0.013145722 | 1.8475543 | up | ankyrinrepeatdomain22 |
| 16743890 | 0.018498672 | 1.8452607 | up | caspase1,apoptosis-relatedcysteinepeptidase |
| 16708489 | 0.024117962 | 1.8447999 | up | deletedinprimaryciliarydyskinesiahomolog(mouse) |
| 16845445 | 0.037462898 | 1.844406 | up | dualspecificityphosphatase3 |
| 17032433 | 0.011767197 | 1.8439801 | up | transporter2,ATP-bindingcassette,sub-familyB(MDR/TAP) |
| 16672478 | 0.00686232 | 1.8425312 | up | SLAMfamilymember8 |
| 17030575 | 0.008030904 | 1.840856 | up | HLAcomplexgroup26(non-proteincoding) |
| 17070634 | 0.01132466 | 1.8387626 | up | receptor-interactingserine-threoninekinase2 |
| 17027749 | 0.008261748 | 1.836645 | up | HLAcomplexgroup26(non-proteincoding) |
| 17020748 | 0.013278823 | 1.8356224 | up | KHhomologydomaincontaining1-like |
| 17035373 | 0.008457693 | 1.834635 | up | HLAcomplexgroup26(non-proteincoding) |
| 16882428 | 0.021655157 | 1.833501 | up | lysine(K)-specificdemethylase3A |
| 17027101 | 0.016543942 | 1.8303599 | up | transporter2,ATP-bindingcassette,sub-familyB(MDR/TAP) |
| 16785952 | 0.002585392 | 1.8290222 | up | pecanexhomolog(Drosophila) |
| 16670367 | 0.00770663 | 1.8286618 | up | FcfragmentofIgG,highaffinityIa,receptor(CD64)\|FcfragmentofIgG,highaffinityIb,receptor(CD64)\|FcfragmentofIgG,highaffinityIc,receptor(CD64),pseudogene |
| 16701398 | 0.022685898 | 1.8280474 | up | SMYD3intronictranscript1(non-proteincoding) |
| 17067170 | 0.00109824 | 1.8252649 | up | BCL2/adenovirusE1B19kDainteractingprotein3-like |
| 16938335 | 0.01384865 | 1.8251511 | up |  |
| 16792194 | 0.003783021 | 1.8206784 | up |  |
| 17004167 | 0.043729737 | 1.8196248 | up | interferonregulatoryfactor4 |
| 16669566 | 0.009816587 | 1.8166444 | up |  |
| 16670197 | 0.010138445 | 1.8162283 | up |  |
| 16981219 | 0.029407203 | 1.8159038 | up | DEAD(Asp-Glu-Ala-Asp)boxpolypeptide60 |
| 17009132 | 0.003403108 | 1.8131028 | up | transmembraneprotein63B |
| 17037228 | 0.016510833 | 1.8121666 | up | transporter2,ATP-bindingcassette,sub-familyB(MDR/TAP) |
| 16968077 | 0.025600404 | 1.807745 | up | cyclinG2 |
| 16787814 | 0.003108635 | 1.806297 | up | interferon,alpha-inducibleprotein27 |
| 16743854 | 0.002665289 | 1.8045108 | up | caspase4,apoptosis-relatedcysteinepeptidase |
| 16987673 | 0.00428563 | 1.7999816 | up | peptidylglycinealpha-amidatingmonooxygenase |
| 16989293 | 0.011520957 | 1.7968922 | up | SEC24family,memberA(S.cerevisiae) |
| 16851747 | 0.01700029 | 1.7954657 | up |  |
| 16786587 | 0.001009525 | 1.795147 | up | FBJmurineosteosarcomaviraloncogenehomolog |
| 16961616 | 0.02731138 | 1.7856798 | up | tumornecrosisfactor(ligand)superfamily,member10 |
| 16738544 | 2.41E-04 | 1.7734232 | up | ubiquitin-conjugatingenzymeE2L6 |
| 16891840 | 0.03987145 | 1.7701434 | up |  |
| 17088164 | 0.005095157 | 1.7694229 | up | solutecarrierfamily31(coppertransporters),member2 |
| 17025230 | 0.002252879 | 1.7694224 | up | T-cellactivationRhoGTPaseactivatingprotein |
| 16700911 | 3.91E-04 | 1.7646624 | up | ERO1-likebeta(S.cerevisiae) |
| 16859681 | 0.006682192 | 1.7641728 | up | potassiumintermediate/smallconductancecalcium-activatedchannel,subfamilyN,member1 |
| 16859509 | 0.003440582 | 1.7628466 | up | familywithsequencesimilarity125,memberA\|uncharacterizedLOC100507535 |
| 17064542 | 1.62E-04 | 1.7553194 | up | microRNA3907 |
| 17029745 | 0.008951441 | 1.7543718 | up | transporter2,ATP-bindingcassette,sub-familyB(MDR/TAP) |
| 16819244 | 0.00526196 | 1.7534616 | up | metallothionein1C,pseudogene |
| 17005197 | 0.006693695 | 1.7525208 | up | ringfingerprotein144B |
| 17103951 | 0.001498176 | 1.7518101 | up | TSPY-like2 |
| 16871527 | 0.020103885 | 1.7507176 | up |  |
| 16810884 | 0.02883129 | 1.7478212 | up |  |
| 16852683 | 0.00372187 | 1.7471864 | up | phorbol-12-myristate-13-acetate-inducedprotein1 |
| 16882332 | 0.020069093 | 1.7459402 | up | granulysin |
| 17076493 | 0.006034839 | 1.7459116 | up |  |
| 16951542 | 0.015666347 | 1.7453576 | up |  |
| 17036173 | 0.024247816 | 1.7435807 | up | interferoninducedtransmembraneprotein4pseudogene |
| 17016760 | 0.023416862 | 1.7379379 | up | interferoninducedtransmembraneprotein4pseudogene |
| 17108767 | 0.005002775 | 1.7379277 | up |  |
| 16702967 | 0.007292372 | 1.7368037 | up | ADP-ribosylationfactor-like5B |
| 17075829 | 0.003345722 | 1.7348447 | up | zincfingerprotein395\|F-boxprotein16 |
| 17027130 | 0.00953259 | 1.734532 | up | proteasome(prosome,macropain)subunit,betatype,8(largemultifunctionalpeptidase7) |
| 16786906 | 0.013846696 | 1.7324896 | up | microRNA1260a |
| 16972155 | 0.043792035 | 1.7319384 | up | methylsterolmonooxygenase1 |
| 16793805 | 0.019342775 | 1.73058 | up | zincfingerandBTBdomaincontaining25 |
| 17017965 | 0.009427498 | 1.7303771 | up | proteasome(prosome,macropain)subunit,betatype,8(largemultifunctionalpeptidase7) |
| 16904365 | 0.024059659 | 1.7299353 | up | interferoninducedwithhelicaseCdomain1 |
| 17042473 | 0.009205081 | 1.7289497 | up | proteasome(prosome,macropain)subunit,betatype,8(largemultifunctionalpeptidase7) |
| 16881687 | 0.0080364 | 1.7228283 | up | WDrepeatdomain54 |
| 17032462 | 0.009583208 | 1.7225679 | up | proteasome(prosome,macropain)subunit,betatype,8(largemultifunctionalpeptidase7) |
| 16855634 | 0.019551506 | 1.7219734 | up |  |
| 17034777 | 0.00941746 | 1.7211868 | up | proteasome(prosome,macropain)subunit,betatype,8(largemultifunctionalpeptidase7) |
| 17029774 | 0.009569243 | 1.7207148 | up | proteasome(prosome,macropain)subunit,betatype,8(largemultifunctionalpeptidase7) |
| 17037257 | 0.009547737 | 1.7193832 | up | proteasome(prosome,macropain)subunit,betatype,8(largemultifunctionalpeptidase7) |
| 16909914 | 0.047280427 | 1.7187251 | up |  |
| 16771417 | 5.79E-04 | 1.7180271 | up | 2'-5'-oligoadenylatesynthetase-like |
| 17039963 | 0.009680684 | 1.7178975 | up | proteasome(prosome,macropain)subunit,betatype,8(largemultifunctionalpeptidase7) |
| 16724849 | 6.17E-04 | 1.7150013 | up | smoothelin-like1 |
| 17073368 | 0.019328438 | 1.7118615 | up |  |
| 16791436 | 0.010132762 | 1.7118522 | up | granzymeH(cathepsinG-like2,proteinh-CCPX) |
| 16742184 | 0.038262237 | 1.7110088 | up |  |
| 16777963 | 0.009905708 | 1.7091758 | up |  |
| 17034759 | 0.03947839 | 1.7068661 | up | transporter2,ATP-bindingcassette,sub-familyB(MDR/TAP) |
| 16793024 | 0.01818009 | 1.7064114 | up | ERO1-like(S.cerevisiae) |
| 16683290 | 0.012272324 | 1.7025384 | up | zincfingerprotein436 |
| 17039945 | 0.040392026 | 1.7025298 | up | transporter2,ATP-bindingcassette,sub-familyB(MDR/TAP) |
| 17028653 | 0.019302886 | 1.7003119 | up | interferoninducedtransmembraneprotein4pseudogene |
| 16669796 | 0.02341851 | 1.6996899 | up | thioredoxininteractingprotein |
| 17067185 | 0.001189532 | 1.692565 | up |  |
| 16988978 | 0.04340675 | 1.691119 | up |  |
| 17050328 | 0.02747971 | 1.6908667 | up | DnaJ(Hsp40)homolog,subfamilyB,member9 |
| 16863287 | 0.009821799 | 1.6873189 | up | FBJmurineosteosarcomaviraloncogenehomologB |
| 16773621 | 0.009242313 | 1.6857417 | up |  |
| 16705638 | 0.048059933 | 1.6857201 | up |  |
| 17000463 | 0.03549481 | 1.6823573 | up |  |
| 16678166 | 0.023838885 | 1.6809537 | up | ITPKBantisenseRNA1(non-proteincoding) |
| 16790362 | 0.016607301 | 1.6798471 | up | smallnucleolarRNA,C/Dbox8 |
| 16849238 | 0.019089496 | 1.6765019 | up | rhomboid5homolog2(Drosophila) |
| 16756224 | 0.004745993 | 1.6763595 | up | microRNA3922 |
| 16681986 | 0.013026824 | 1.6738688 | up |  |
| 17040665 | 0.017599385 | 1.6727294 | up | HLAcomplexP5(non-proteincoding) |
| 16885118 | 4.51E-04 | 1.6720173 | up | v-ralsimianleukemiaviraloncogenehomologB(rasrelated |
| 16833327 | 0.007356709 | 1.6716303 | up | schlafenfamilymember5 |
| 16974115 | 0.001576741 | 1.6710008 | up |  |
| 17005611 | 0.037318822 | 1.6672606 | up | butyrophilin,subfamily3,memberA2 |
| 16877048 | 0.002963572 | 1.6666036 | up | hippocalcin-like1 |
| 16733995 | 0.001810403 | 1.6658242 | up | interferonregulatoryfactor7 |
| 16960114 | 0.03048457 | 1.6612761 | up | procollagen-lysine,2-oxoglutarate5-dioxygenase2 |
| 16788488 | 0.007406677 | 1.6605675 | up | maternallyexpressed3(non-proteincoding) |
| 16948589 | 0.045447715 | 1.6542058 | up | YEATSdomaincontaining2 |
| 16686060 | 7.67E-04 | 1.6508431 | up | solutecarrierfamily2(facilitatedglucosetransporter),member1 |
| 16720093 | 6.42E-04 | 1.648494 | up |  |
| 16879335 | 2.21E-04 | 1.6476187 | up | transmembraneprotein178A |
| 16979256 | 0.003666833 | 1.647358 | up | SEC24family,memberD(S.cerevisiae) |
| 17031185 | 7.07E-04 | 1.64724 | up |  |
| 16786650 | 0.03014008 | 1.6443781 | up | RNA,5Sribosomal387 |
| 16870200 | 0.03851215 | 1.6426247 | up | bonemarrowstromalcellantigen2 |
| 16669698 | 0.011000222 | 1.6426072 | up |  |
| 16972961 | 0.010772483 | 1.6410422 | up | chromosome4openreadingframe47 |
| 16747402 | 0.01162475 | 1.6396188 | up | lymphocyte-activationgene3 |
| 17106803 | 0.04790022 | 1.6395031 | up |  |
| 16743364 | 0.024328629 | 1.6394385 | up |  |
| 16711562 | 0.038091287 | 1.6387612 | up | Scm-likewithfourmbtdomains2 |
| 17017947 | 0.03845254 | 1.6374854 | up | transporter2,ATP-bindingcassette,sub-familyB(MDR/TAP) |
| 16931331 | 1.31E-04 | 1.6353241 | up | microRNA3619 |
| 17092788 | 0.025140459 | 1.6329865 | up | microRNA4474 |
| 16733790 | 0.024373986 | 1.6304122 | up |  |
| 16974521 | 0.002061238 | 1.6303767 | up |  |
| 16704347 | 0.018802194 | 1.630013 | up |  |
| 16928098 | 0.005570167 | 1.6297187 | up | macrophagemigrationinhibitoryfactor(glycosylation-inhibitingfactor) |
| 16881838 | 0.0411689 | 1.6294484 | up | hexokinase2 |
| 16926200 | 0.001834652 | 1.6266518 | up | salt-induciblekinase1 |
| 16998332 | 0.043234162 | 1.6262351 | up |  |
| 17040387 | 0.014619471 | 1.6239971 | up | majorhistocompatibilitycomplex,classI,A |
| 16875145 | 0.020982292 | 1.6220129 | up | zincfingerprotein160 |
| 16995890 | 0.028198393 | 1.6216041 | up | 3-hydroxy-3-methylglutaryl-CoAsynthase1(soluble) |
| 16712601 | 0.021090638 | 1.6214014 | up |  |
| 16782684 | 0.007309607 | 1.6204281 | up | interferonregulatoryfactor9 |
| 16962022 | 0.048325684 | 1.6184283 | up | lysosomal-associatedmembraneprotein3 |
| 16709108 | 0.03459132 | 1.6178006 | up | MAXinteractor1 |
| 17026419 | 0.001367464 | 1.6125119 | up |  |
| 16999712 | 0.005348609 | 1.6122988 | up | prolyl4-hydroxylase,alphapolypeptideII |
| 16913204 | 0.04740926 | 1.6114218 | up |  |
| 17035925 | 0.001327575 | 1.6107514 | up |  |
| 16805675 | 0.033807073 | 1.6104314 | up |  |
| 16681981 | 0.003476198 | 1.6094218 | up | chromosome1openreadingframe195 |
| 17041505 | 0.012512572 | 1.6077509 | up | HLA-FantisenseRNA1(non-proteincoding) |
| 16861012 | 0.026549557 | 1.6063814 | up | freefattyacidreceptor3 |
| 17053773 | 0.04135157 | 1.6026919 | up |  |
| 16999886 | 0.00357353 | 1.6016723 | up | RNA,U7smallnuclear14pseudogene\|RNA,U7smallnuclear15pseudogene |
| 17041267 | 0.001379376 | 1.6015638 | up |  |
| 16827474 | 0.040421847 | 1.6012962 | up | chymotrypsin-like |
| 16958529 | 0.007843779 | 1.6000677 | up |  |
| 16928293 | 0.008458983 | 1.598204 | up | gamma-glutamyltransferase2\|gamma-glutamyltransferase1\|smallnuclearribonucleoproteinD3polypeptide18kDa |
| 16813922 | 0.001364869 | 1.5976341 | up | proproteinconvertasesubtilisin/kexintype6\|uncharacterizedLOC100507472 |
| 16923417 | 0.016705133 | 1.5972598 | up | 1-acylglycerol-3-phosphateO-acyltransferase3 |
| 16797389 | 0.009864979 | 1.5946246 | up | immunoglobulinheavyconstantgamma3(G3mmarker)\|immunoglobulinheavyconstantmu |
| 17053654 | 0.013501743 | 1.5938574 | up | negativeregulatorofubiquitin-likeproteins1 |
| 17083567 | 0.030374693 | 1.5922825 | up |  |
| 16949261 | 0.04687874 | 1.5912336 | up |  |
| 17080334 | 0.02015436 | 1.5889549 | up |  |
| 16967957 | 0.006588294 | 1.5885444 | up |  |
| 16763195 | 0.016212592 | 1.5839921 | up | solutecarrierfamily2(facilitatedglucosetransporter),member13 |
| 16670309 | 0.026197065 | 1.5820354 | up | familywithsequencesimilarity91,memberA2\|uncharacterizedLOC388692 |
| 17029100 | 0.04124577 | 1.5806329 | up |  |
| 17086048 | 0.023913449 | 1.5803744 | up | glucosaminyl(N-acetyl)transferase1,core2 |
| 16920468 | 0.004340058 | 1.5798484 | up | RNA,U7smallnuclear14pseudogene\|RNA,U7smallnuclear15pseudogene |
| 17075757 | 0.048455995 | 1.5796922 | up | microRNA3622b |
| 16802939 | 0.00859639 | 1.5786623 | up |  |
| 16769804 | 0.00645956 | 1.5784452 | up |  |
| 17041881 | 0.041828185 | 1.5781952 | up |  |
| 17032957 | 0.013589184 | 1.5748384 | up | majorhistocompatibilitycomplex,classI,L(pseudogene) |
| 16890723 | 0.041732118 | 1.5738258 | up | RUNandFYVEdomaincontaining4 |
| 17117911 | 0.029724982 | 1.5731213 | up | IQmotifandSec7domain3 |
| 16908998 | 0.009772455 | 1.5729662 | up | WDrepeatandFYVEdomaincontaining1 |
| 16690036 | 0.005151532 | 1.5721965 | up | ferric-chelatereductase1 |
| 17063681 | 0.01714527 | 1.5721285 | up |  |
| 16735636 | 0.0415834 | 1.5711066 | up |  |
| 16701841 | 0.010984749 | 1.5707276 | up | phosphofructokinase,platelet |
| 16708570 | 0.0272369 | 1.5704708 | up |  |
| 16770780 | 0.036081787 | 1.5694928 | up |  |
| 16935223 | 0.035580814 | 1.5674406 | up |  |
| 16927827 | 0.018860424 | 1.5657712 | up |  |
| 17085452 | 0.044524107 | 1.5651791 | up |  |
| 16862491 | 0.010714017 | 1.5651777 | up | transmembraneprotein91 |
| 17079910 | 0.02793313 | 1.5649899 | up | Kruppel-likefactor10 |
| 16947343 | 0.00541538 | 1.5628159 | up | cyclinL1 |
| 16715631 | 0.039135907 | 1.5621349 | up |  |
| 16684812 | 0.015088475 | 1.5620196 | up | ringfingerprotein19B |
| 16662993 | 0.04513247 | 1.5576937 | up | rearrangedL-mycfusion |
| 16932120 | 0.035042603 | 1.5567764 | up |  |
| 17075868 | 0.03854864 | 1.5566405 | up | microRNA4288 |
| 17007299 | 0.017933872 | 1.5561116 | up | uncharacterizedLOC100507463\|proteasome(prosome,macropain)subunit,betatype,9(largemultifunctionalpeptidase2) |
| 17033653 | 0.018432694 | 1.5558901 | up |  |
| 16734420 | 0.017274758 | 1.5556864 | up | achaete-scutecomplexhomolog2(Drosophila) |
| 16931636 | 0.012797685 | 1.5535204 | up | pannexin2 |
| 17057381 | 8.75E-04 | 1.5526037 | up | myosinIG |
| 17038636 | 0.0186068 | 1.5525073 | up |  |
| 16743554 | 0.00311361 | 1.5522312 | up | RNA,5Sribosomal346 |
| 16842403 | 0.001695641 | 1.5522202 | up | lectin,galactoside-binding,soluble,9B\|lectin,galactoside-binding,soluble,9C |
| 17095564 | 0.029326927 | 1.5508895 | up | microRNA4289 |
| 16702210 | 0.02313418 | 1.5500097 | up | microRNA3155a |
| 16888905 | 0.037031535 | 1.5496253 | up |  |
| 17057424 | 0.002056418 | 1.5494704 | up | NACalphadomaincontaining |
| 16661071 | 0.003154849 | 1.5491989 | up | connectorenhancerofkinasesuppressorofRas1 |
| 16858970 | 0.040491328 | 1.5487516 | up |  |
| 16875014 | 0.0361907 | 1.5487101 | up | zincfingerprotein841 |
| 17028352 | 0.018981472 | 1.5479544 | up |  |
| 16866849 | 0.001517147 | 1.5473226 | up | MAPkinaseinteractingserine/threoninekinase2 |
| 17011279 | 0.012558245 | 1.5449362 | up | PRdomaincontaining1,withZNFdomain |
| 17074490 | 0.010650759 | 1.5415488 | up | proteinphosphatase1,regulatorysubunit3B |
| 16903913 | 0.018785033 | 1.5409846 | up |  |
| 17110670 | 0.002748891 | 1.5403647 | up | pim-2oncogene |
| 16830883 | 0.046711344 | 1.5384982 | up | arachidonate15-lipoxygenase,typeB |
| 16772766 | 0.005597022 | 1.5384082 | up |  |
| 16719624 | 0.034097683 | 1.5382968 | up |  |
| 16857842 | 0.03956019 | 1.5363015 | up | chemokine(C-Cmotif)ligand25 |
| 16843635 | 0.028971383 | 1.534323 | up | TBC1domainfamily,member3H\|TBC1domainfamily,member3F\|TBC1domainfamily,member3G\|TBC1domainfamily,member3B\|TBC1domainfamily,member3C\|TBC1domainfamilymember3H-like\|DEAH(Asp-Glu-Ala-His)boxpolypeptide40pseudogene |
| 16703977 | 0.036532063 | 1.5342809 | up |  |
| 16858710 | 0.026940353 | 1.5338959 | up | junBproto-oncogene |
| 16815898 | 0.001244431 | 1.5329446 | up |  |
| 16941049 | 0.012793223 | 1.5327054 | up |  |
| 16857211 | 0.035388764 | 1.532127 | up | microRNA4746 |
| 17025177 | 4.78E-06 | 1.5319026 | up | dynein,lightchain,Tctex-type1 |
| 17114017 | 0.03657357 | 1.5317069 | up | zincfinger,DHHC-typecontaining9 |
| 16774303 | 0.007979617 | 1.5316339 | up | regulatorofcellcycle |
| 16699932 | 0.008439192 | 1.531218 | up | transmembraneprotein63A |
| 16839019 | 0.004791246 | 1.531044 | up | solutecarrierfamily16,member3(monocarboxylicacidtransporter4) |
| 16997858 | 0.003513326 | 1.5308114 | up |  |
| 16787151 | 0.005706998 | 1.5302254 | up |  |
| 16816171 | 0.034685247 | 1.529883 | up |  |
| 17035022 | 0.016871084 | 1.529515 | up | majorhistocompatibilitycomplex,classI,F |
| 16819197 | 0.037987486 | 1.5286978 | up | metallothionein4 |
| 17007810 | 0.003109744 | 1.5284452 | up | peroxisomeproliferator-activatedreceptordelta |
| 16661497 | 0.020191401 | 1.5250027 | up | chromosome1openreadingframe38 |
| 16885874 | 0.005851142 | 1.5243498 | up | mannosyl(alpha-1,6-)-glycoproteinbeta-1,6-N-acetyl-glucosaminyltransferase |
| 16903111 | 0.017061312 | 1.5230539 | up | aspartyl-tRNAsynthetase |
| 16838386 | 0.033476185 | 1.5227733 | up |  |
| 16862504 | 0.005554357 | 1.522086 | up | branchedchainketoaciddehydrogenaseE1,alphapolypeptide |
| 17052392 | 0.030112434 | 1.520936 | up | uncharacterizedLOC285962 |
| 16784365 | 0.008618872 | 1.5207863 | up |  |
| 16824123 | 0.03481059 | 1.520215 | up | microRNA3180-2\|microRNA3180-1\|microRNA3180-3 |
| 16698543 | 0.023703486 | 1.5199893 | up | RAB7,memberRASoncogenefamily-like1 |
| 16788725 | 0.025729353 | 1.5194896 | up | microRNA329-1 |
| 16735596 | 0.027708974 | 1.5186883 | up | DENN/MADDdomaincontaining5A |
| 17042844 | 0.013254669 | 1.5182668 | up | Gprotein-coupledreceptor146\|uncharacterizedLOC100505568\|uncharacterizedLOC100505551 |
| 17075650 | 0.003158873 | 1.517382 | up | paraneoplasticMaantigen2 |
| 16719903 | 0.040612787 | 1.5166795 | up | microRNA3944 |
| 17036486 | 0.001054449 | 1.5165609 | up | majorhistocompatibilitycomplex,classI,C |
| 16913196 | 0.048736885 | 1.5153762 | up |  |
| 16774623 | 0.032579012 | 1.5136744 | up | leucine-richrepeatsandcalponinhomology(CH)domaincontaining1 |
| 16995534 | 0.02009705 | 1.512632 | up | microRNA3650 |
| 16671784 | 0.008713704 | 1.5120887 | up | POUclass5homeobox1pseudogene4 |
| 16820034 | 0.012444178 | 1.511997 | up | nucleolarprotein3(apoptosisrepressorwithCARDdomain) |
| 17010789 | 0.013835948 | 1.5106622 | up | zincfingerprotein292 |
| 16691929 | 0.024640355 | 1.5101491 | up | uncharacterizedLOC728855\|uncharacterizedLOC728875 |
| 16805804 | 0.013773348 | 1.5092034 | up | familywithsequencesimilarity138,memberE |
| 16835213 | 0.040055294 | 1.5086358 | up | aminopeptidasepuromycinsensitive\|uncharacterizedLOC100653042 |
| 16673341 | 0.03651878 | 1.5082189 | up | RNA,5Sribosomal65 |
| 16891261 | 0.005125831 | 1.508071 | up | desmin |
| 16872551 | 0.012800721 | 1.5077859 | up | transforminggrowthfactor,beta1 |
| 16849939 | 0.012701754 | 1.507292 | up | prolyl4-hydroxylase,betapolypeptide |
| 16916863 | 0.02524429 | 1.5067332 | up | ringfingerprotein24 |
| 16823860 | 0.017435549 | 1.5067209 | up | Dexihomolog(mouse) |
| 16772544 | 0.012753773 | 1.50668 | up | RNA,5Sribosomal378 |
| 16820448 | 0.04748745 | 1.5051384 | up |  |
| 16835299 | 0.03176861 | 1.5047857 | up | TBK1bindingprotein1 |
| 16658064 | 0.012060983 | 1.5043825 | up | tumornecrosisfactorreceptorsuperfamily,member14 |
| 16844775 | 0.002230288 | 1.503246 | up | keratin19 |
| 16858884 | 0.016767668 | 1.5022162 | up | microRNA181d |
| 17112586 | 0.036004942 | 1.5018003 | up |  |
| 16943425 | 0.015218938 | 1.5017982 | up |  |

| **Transcript Cluster Id** | **p value** | **FC (abs)** | **Regulation** | **Gene Description** |
| --- | --- | --- | --- | --- |
| 16894706 | 0.002721402 | 9.964618 | down |  |
| 17016403 | 0.002384855 | 6.2334046 | down | histonecluster1,H3g\|histonecluster1,H3f\|histonecluster1,H3b\|histonecluster1,H3h\|histonecluster1,H3j\|histonecluster1,H3i\|histonecluster1,H3e\|histonecluster1,H3c\|histonecluster1,H3d\|histonecluster1,H3a |
| 17056815 | 0.033798326 | 5.7455177 | down | Tcellreceptorgammaconstant2\|TcellreceptorgammajoiningP2 |
| 16672435 | 1.34E-05 | 5.1474104 | down | FcfragmentofIgE,highaffinityI,receptorfor |
| 16781610 | 8.53E-06 | 4.9314423 | down | ribonuclease,RNaseAfamily,2(liver,eosinophil-derivedneurotoxin) |
| 16662427 | 0.001588449 | 4.347428 | down |  |
| 16725041 | 1.40E-04 | 4.2726827 | down | familywithsequencesimilarity111,memberB |
| 16834056 | 8.84E-05 | 4.0697036 | down | celldivisioncycle6homolog(S.cerevisiae) |
| 16843309 | 0.024506994 | 4.013354 | down | chemokine(C-Cmotif)ligand1 |
| 16793190 | 0.005979249 | 3.9879947 | down | WDrepeatandHMG-boxDNAbindingprotein1 |
| 16983795 | 0.0069886 | 3.9753299 | down |  |
| 16677201 | 4.37E-04 | 3.829931 | down | denticlelessE3ubiquitinproteinligasehomolog(Drosophila) |
| 16872248 | 0.032157786 | 3.8282864 | down | Charcot-Leydencrystalprotein |
| 16946744 | 9.90E-04 | 3.8005474 | down | carboxypeptidaseA3(mastcell) |
| 16828886 | 6.78E-04 | 3.7707672 | down | GINScomplexsubunit2(Psf2homolog) |
| 16903090 | 9.82E-05 | 3.758514 | down | minichromosomemaintenancecomplexcomponent6 |
| 17080595 | 2.04E-04 | 3.6955516 | down | defectiveinsisterchromatidcohesion1homolog(S.cerevisiae) |
| 16978036 | 0.001242856 | 3.6232743 | down | hematopoieticprostaglandinDsynthase |
| 16916958 | 0.001749226 | 3.5580866 | down | proliferatingcellnuclearantigen |
| 17072159 | 0.015165786 | 3.4435205 | down |  |
| 16767364 | 0.005446726 | 3.4006238 | down |  |
| 17096433 | 0.029728768 | 3.3987536 | down |  |
| 16982024 | 0.012883634 | 3.3793232 | down | MLF1interactingprotein |
| 16755900 | 0.035298213 | 3.3723536 | down |  |
| 16696531 | 0.04996342 | 3.3680694 | down | smallnucleolarRNA,C/Dbox78 |
| 16847432 | 6.06E-04 | 3.2488828 | down | BRCA1interactingproteinC-terminalhelicase1 |
| 16879883 | 0.003071192 | 3.2098534 | down | mutShomolog2,coloncancer,nonpolyposistype1(E.coli) |
| 16911212 | 4.65E-04 | 3.2020466 | down | minichromosomemaintenancecomplexcomponent8 |
| 16738845 | 0.048939552 | 3.19725 | down | membrane-spanning4-domains,subfamilyA,member4E |
| 16828705 | 0.01735815 | 3.1961944 | down | M-phasephosphoprotein6 |
| 16996722 | 0.003222459 | 3.1896536 | down | centromereproteinK |
| 16860297 | 0.01864178 | 3.1751943 | down |  |
| 17016499 | 0.006593861 | 3.1744509 | down | histonecluster1,H1b |
| 16937505 | 1.14E-04 | 3.1693594 | down | Fanconianemia,complementationgroupD2 |
| 16933562 | 0.03703205 | 3.16317 | down |  |
| 16707695 | 4.92E-04 | 3.155317 | down | helicase,lymphoid-specific |
| 16852871 | 1.48E-04 | 3.1375294 | down | serpinpeptidaseinhibitor,cladeB(ovalbumin),member2\|serpinpeptidaseinhibitor,cladeB(ovalbumin),member10 |
| 16844312 | 0.001351088 | 3.1367152 | down | topoisomerase(DNA)IIalpha170kDa |
| 16851397 | 0.003684485 | 3.13359 | down | retinoblastomabindingprotein8 |
| 17072669 | 3.84E-04 | 3.0991225 | down | v-mycmyelocytomatosisviraloncogenehomolog(avian) |
| 16980051 | 0.007560009 | 3.0810924 | down | calmegin |
| 16800355 | 5.43E-04 | 3.069783 | down | WDrepeatdomain76 |
| 17020019 | 9.70E-04 | 3.0663853 | down | minichromosomemaintenancecomplexcomponent3 |
| 16810976 | 0.026592866 | 3.0630834 | down | smallnucleolarRNA,C/Dbox18A\|smallnucleolarRNA,C/Dbox18C\|smallnucleolarRNA,C/Dbox18B\|smallnucleolarRNA,C/Dbox16\|ribosomalproteinL4 |
| 17070381 | 7.76E-04 | 3.0499516 | down | leucinerichrepeatandcoiled-coilcentrosomalprotein1 |
| 16802106 | 0.001579761 | 3.0266666 | down | proteintyrosinephosphatase-likeAdomaincontaining1 |
| 16911149 | 0.002269808 | 3.0240736 | down | PCNAantisenseRNA1(non-proteincoding) |
| 17078626 | 0.003780127 | 3.0138469 | down | solutecarrierfamily10(sodium/bileacidcotransporterfamily),member5 |
| 17016496 | 0.028253522 | 2.987887 | down | histonecluster1,H2ak\|histonecluster1,H2ah\|histonecluster1,H2ag\|histonecluster1,H2am\|histonecluster1,H2al\|histonecluster1,H2ai |
| 16720799 | 0.012501409 | 2.9727688 | down |  |
| 17010273 | 0.003108576 | 2.9580717 | down | KCNQ5intronictranscript1(non-proteincoding) |
| 16685165 | 0.004672052 | 2.9341333 | down | claspin |
| 17070013 | 0.004271695 | 2.925627 | down | retinoldehydrogenase10(all-trans) |
| 16756642 | 1.38E-04 | 2.9156373 | down |  |
| 16799315 | 0.001153023 | 2.8952634 | down | thrombospondin1 |
| 16756627 | 6.20E-04 | 2.88588 | down | uracil-DNAglycosylase |
| 16725172 | 0.043047875 | 2.870191 | down | membrane-spanning4-domains,subfamilyA,member2 |
| 17076867 | 6.39E-04 | 2.8530915 | down | proteinkinase,DNA-activated,catalyticpolypeptide |
| 17068782 | 0.008342227 | 2.8475492 | down | minichromosomemaintenancecomplexcomponent4 |
| 17016512 | 0.002849554 | 2.8357615 | down | histonecluster1,H2am\|histonecluster1,H2ah\|histonecluster1,H2ag\|histonecluster1,H2al\|histonecluster1,H2ak\|histonecluster1,H2ai |
| 17060412 | 0.005876114 | 2.8340862 | down | minichromosomemaintenancecomplexcomponent7 |
| 16799598 | 3.41E-04 | 2.8335557 | down | cancersusceptibilitycandidate5 |
| 16780808 | 0.002357346 | 2.8307955 | down | KDEL(Lys-Asp-Glu-Leu)containing1 |
| 16667037 | 7.56E-05 | 2.824364 | down | celldivisioncycle7homolog(S.cerevisiae) |
| 16721835 | 7.55E-04 | 2.8173335 | down |  |
| 16702571 | 7.58E-04 | 2.8104033 | down | minichromosomemaintenancecomplexcomponent10 |
| 16679411 | 0.00200951 | 2.8099241 | down | exonuclease1 |
| 16804559 | 0.004520591 | 2.8077438 | down | Fanconianemia,complementationgroupI |
| 16753923 | 0.001115233 | 2.7999907 | down | YEATSdomaincontaining4 |
| 16690427 | 6.56E-04 | 2.7981484 | down | HEN1methyltransferasehomolog1(Arabidopsis) |
| 16850477 | 0.003667849 | 2.7948353 | down | thymidylatesynthetase |
| 16773946 | 0.004980056 | 2.792237 | down | replicationfactorC(activator1)3,38kDa |
| 17076963 | 0.012733181 | 2.7793903 | down |  |
| 16792519 | 0.001596999 | 2.7677114 | down | polymerase(DNAdirected),epsilon2,accessorysubunit |
| 16952704 | 0.03351561 | 2.757244 | down |  |
| 16793750 | 0.00177864 | 2.7549107 | down | sphingosine-1-phosphatephosphatase1 |
| 16701106 | 9.25E-04 | 2.7521145 | down | choroideremia-like(Rabescortprotein2) |
| 16901974 | 0.045301545 | 2.7482066 | down | interleukin1,alpha |
| 16681611 | 0.003010579 | 2.7481487 | down | spermidinesynthase |
| 16983740 | 0.023124183 | 2.7153575 | down |  |
| 16855673 | 0.024521949 | 2.711688 | down | B-cellCLL/lymphoma2 |
| 16957170 | 0.003917281 | 2.7074301 | down |  |
| 16841768 | 0.001563686 | 2.7021399 | down | centromereproteinV |
| 17020733 | 0.011582753 | 2.7019992 | down | KCNQ5antisenseRNA2(non-proteincoding) |
| 16833295 | 0.037044678 | 2.6938987 | down |  |
| 16712292 | 0.002774481 | 2.6692457 | down | proteintyrosinephosphatase-like(prolineinsteadofcatalyticarginine),memberA |
| 16748160 | 0.035444718 | 2.665261 | down |  |
| 16887840 | 3.19E-04 | 2.6587863 | down | celldivisioncycleassociated7 |
| 17056825 | 0.005103451 | 2.6411264 | down | TCRgammaalternatereadingframeprotein\|TcellreceptorgammajoiningP1 |
| 16758336 | 2.37E-04 | 2.6401029 | down | kinetochoreassociated1 |
| 17105076 | 0.018225415 | 2.6233723 | down | purinergicreceptorP2Y,G-proteincoupled,10 |
| 16912379 | 0.001238498 | 2.622946 | down |  |
| 16773076 | 0.005376961 | 2.6198218 | down | longintergenicnon-proteincodingRNA539 |
| 16976827 | 0.029709967 | 2.616913 | down | chemokine(C-X-Cmotif)ligand5 |
| 16919044 | 5.83E-05 | 2.601394 | down | retinoblastoma-like1(p107) |
| 16883690 | 0.00177482 | 2.6001523 | down | interleukin1receptor-like1 |
| 16784519 | 5.30E-04 | 2.5938292 | down |  |
| 16802204 | 8.92E-04 | 2.5853 | down | Zwilch,kinetochoreassociated,homolog(Drosophila) |
| 16989332 | 0.016969489 | 2.5850334 | down | DEAD(Asp-Glu-Ala-Asp)boxpolypeptide46 |
| 16693406 | 0.018641833 | 2.5753226 | down |  |
| 17019683 | 0.002301163 | 2.574091 | down |  |
| 16979389 | 8.15E-04 | 2.5705934 | down | MAD2mitoticarrestdeficient-like1(yeast) |
| 17064679 | 0.012759283 | 2.5675952 | down |  |
| 16970563 | 0.010597083 | 2.5634656 | down | polo-likekinase4 |
| 16762657 | 0.001110251 | 2.5581584 | down |  |
| 16738258 | 0.00973412 | 2.553066 | down |  |
| 16722299 | 0.001260926 | 2.5530138 | down | phosphodiesterase3B,cGMP-inhibited |
| 16851933 | 2.07E-04 | 2.546982 | down | UDP-N-acetyl-alpha-D-galactosamine:polypeptideN-acetylgalactosaminyltransferase1(GalNAc-T1) |
| 17016400 | 0.00797014 | 2.5447721 | down | histonecluster1,H3f\|histonecluster1,H3b\|histonecluster1,H3h\|histonecluster1,H3j\|histonecluster1,H3g\|histonecluster1,H3i\|histonecluster1,H3e\|histonecluster1,H3c\|histonecluster1,H3d\|histonecluster1,H3a |
| 16906419 | 0.003800128 | 2.533314 | down | solutecarrierfamily40(iron-regulatedtransporter),member1 |
| 16967831 | 0.003042526 | 2.520818 | down | epithelialmitogenhomolog(mouse) |
| 17072723 | 0.0316228 | 2.5142913 | down |  |
| 16661192 | 0.001659421 | 2.5129836 | down | highmobilitygroupnucleosomalbindingdomain2 |
| 16712357 | 0.003325841 | 2.5067108 | down |  |
| 16927052 | 0.008928977 | 2.505485 | down | celldivisioncycle45homolog(S.cerevisiae) |
| 16857192 | 0.001622942 | 2.5005329 | down | chromatinassemblyfactor1,subunitA(p150) |
| 16807268 | 0.001673801 | 2.4985595 | down |  |
| 16951485 | 0.00513654 | 2.4974718 | down | shugoshin-like1(S.pombe) |
| 16821239 | 0.002200944 | 2.4972095 | down | centromereproteinN |
| 16908948 | 0.019914972 | 2.4951966 | down | phenylalanyl-tRNAsynthetase,betasubunit |
| 16996030 | 0.015375624 | 2.4937644 | down | embigin |
| 16667760 | 0.003266732 | 2.4936838 | down | sphingosine-1-phosphatereceptor1 |
| 16773840 | 0.005973387 | 2.4929795 | down | breastcancer2,earlyonset |
| 16784299 | 0.018026356 | 2.4862275 | down | cyclin-dependentkinaseinhibitor3 |
| 16979515 | 3.39E-04 | 2.475555 | down | cyclinA2 |
| 16987298 | 0.003243905 | 2.4732692 | down | ankyrinrepeatdomain32 |
| 16858488 | 0.046156578 | 2.4691167 | down |  |
| 16722799 | 5.03E-04 | 2.4566975 | down | proteinargininemethyltransferase3 |
| 16938296 | 3.36E-05 | 2.4534564 | down | SGOL1antisenseRNA1(non-proteincoding) |
| 16733104 | 0.002822502 | 2.4511483 | down | checkpointkinase1 |
| 16985614 | 0.003819635 | 2.4497914 | down | centromereproteinH |
| 16810933 | 0.004707443 | 2.444894 | down | TIMELESSinteractingprotein |
| 16707221 | 0.011624013 | 2.4442043 | down | kinesinfamilymember20B |
| 16747014 | 0.003067169 | 2.4437332 | down | RAD51associatedprotein1 |
| 16976853 | 0.008867635 | 2.4375188 | down |  |
| 16687188 | 3.21E-04 | 2.427589 | down | originrecognitioncomplex,subunit1 |
| 16675278 | 0.028186336 | 2.4202838 | down | regulatorofG-proteinsignaling18 |
| 16935551 | 0.012608445 | 2.4199948 | down | smallnucleolarRNA,C/Dbox13pseudogene1 |
| 16894402 | 0.006172148 | 2.413892 | down | ornithinedecarboxylase1 |
| 17043843 | 0.011031108 | 2.410725 | down | tetraspanin13 |
| 16766318 | 0.001171241 | 2.4098802 | down | primase,DNA,polypeptide1(49kDa) |
| 16808793 | 4.06E-05 | 2.4083648 | down | fibrillin1 |
| 17087716 | 0.0019263 | 2.4079971 | down | structuralmaintenanceofchromosomes2 |
| 16806820 | 0.036184546 | 2.4066584 | down |  |
| 16752217 | 0.013408705 | 2.406169 | down | growthdifferentiationfactor11 |
| 16850394 | 0.002525262 | 2.4033267 | down |  |
| 16755750 | 0.002783323 | 2.3975103 | down |  |
| 17102230 | 0.00247539 | 2.3950248 | down | polymerase(DNAdirected),alpha1,catalyticsubunit |
| 16741584 | 4.74E-04 | 2.388088 | down |  |
| 16673557 | 0.003833052 | 2.3868573 | down | chromosome1openreadingframe112 |
| 16965346 | 0.002803962 | 2.3868093 | down | non-SMCcondensinIcomplex,subunitG |
| 17047459 | 0.012861537 | 2.3836834 | down | smallnucleolarRNA,H/ACAbox14A |
| 17086167 | 0.045903005 | 2.3772516 | down | centrosomalprotein78kDa |
| 17086193 | 0.024754405 | 2.3749602 | down | phosphoserineaminotransferase1 |
| 16850412 | 0.007656468 | 2.3675747 | down |  |
| 16860418 | 8.30E-04 | 2.3656373 | down | cyclinE1 |
| 16721126 | 0.001643341 | 2.3652532 | down | ribonucleotidereductaseM1 |
| 16870984 | 0.021356316 | 2.3540773 | down | zincfingerprotein681 |
| 16799793 | 4.05E-04 | 2.3538513 | down | nucleolarandspindleassociatedprotein1 |
| 17067632 | 0.013405879 | 2.3487785 | down | Wernersyndrome,RecQhelicase-like |
| 17021845 | 1.46E-04 | 2.3426764 | down | MMS22-like,DNArepairprotein |
| 16688665 | 0.008672666 | 2.3414643 | down | phosphatidylinositolglycananchorbiosynthesis,classK |
| 17117788 | 0.017914869 | 2.3395267 | down |  |
| 16877473 | 0.016861802 | 2.3383136 | down | Genendonucleasehomolog1(Drosophila) |
| 17064598 | 0.007338134 | 2.3370724 | down |  |
| 16698023 | 9.01E-04 | 2.3370528 | down | ubiquitin-conjugatingenzymeE2T(putative) |
| 16852312 | 0.00314056 | 2.3364966 | down | spindleandkinetochoreassociatedcomplexsubunit1 |
| 16827170 | 0.02338712 | 2.3363118 | down | NEDD8activatingenzymeE1subunit1 |
| 16742636 | 0.026804417 | 2.3353488 | down |  |
| 16672838 | 0.04141586 | 2.334444 | down | FcfragmentofIgE,highaffinityI,receptorfor |
| 17013251 | 0.019364905 | 2.3248096 | down |  |
| 16684136 | 0.01652605 | 2.3174875 | down | EYA3intronictranscript1(non-proteincoding) |
| 16929573 | 0.01435456 | 2.3148692 | down | minichromosomemaintenancecomplexcomponent5 |
| 16787540 | 0.005768852 | 2.3115294 | down | cleavageandpolyadenylationspecificfactor2,100kDa |
| 16714998 | 0.006855454 | 2.3094625 | down | DNAreplicationhelicase2homolog(yeast) |
| 16829580 | 0.004362319 | 2.3078978 | down | replicationproteinA1,70kDa |
| 16744125 | 0.001405224 | 2.3052237 | down | KDEL(Lys-Asp-Glu-Leu)containing2 |
| 16925450 | 0.007981242 | 2.2946618 | down |  |
| 17004747 | 0.005791932 | 2.291661 | down | PAK1interactingprotein1 |
| 16984032 | 5.45E-04 | 2.2912092 | down | S-phasekinase-associatedprotein2,E3ubiquitinproteinligase |
| 16678561 | 0.004706548 | 2.2910378 | down |  |
| 16971573 | 0.004021254 | 2.2909439 | down | meioticnucleardivisions1homolog(S.cerevisiae) |
| 16804902 | 0.004182928 | 2.2897453 | down | Bloomsyndrome,RecQhelicase-like |
| 17016457 | 0.004972385 | 2.2887104 | down | histonecluster1,H2bk |
| 16850517 | 0.008182787 | 2.2834823 | down |  |
| 17058460 | 0.005727578 | 2.2807918 | down | bromodomainadjacenttozincfingerdomain,1B |
| 16828327 | 0.00188214 | 2.2769072 | down | ringfingerandWDrepeatdomain3 |
| 16701077 | 0.006998364 | 2.2764747 | down | fumaratehydratase |
| 16736422 | 7.10E-04 | 2.2747319 | down | serumamyloidA-like1 |
| 16719285 | 0.044855773 | 2.2732363 | down |  |
| 16961016 | 0.01997147 | 2.2686045 | down | karyopherinalpha4(importinalpha3) |
| 16663014 | 0.017905068 | 2.2683244 | down | zincmetallopeptidaseSTE24homolog(S.cerevisiae) |
| 16962493 | 0.007226062 | 2.2663932 | down | replicationfactorC(activator1)4,37kDa |
| 16847209 | 0.017606555 | 2.2657735 | down | tripartitemotifcontaining37 |
| 17010552 | 0.002841344 | 2.263362 | down |  |
| 16707468 | 0.002473425 | 2.2610567 | down | kinesinfamilymember11 |
| 16733129 | 0.003652312 | 2.2566783 | down |  |
| 16813342 | 4.22E-04 | 2.2535563 | down | proteinregulatorofcytokinesis1 |
| 16985079 | 0.008683039 | 2.2512069 | down | NADHdehydrogenase(ubiquinone)complexI,assemblyfactor2 |
| 16967853 | 0.04706361 | 2.2487452 | down | amphiregulin |
| 16878583 | 0.031647135 | 2.2477698 | down | smallnucleolarRNA,C/Dbox92 |
| 16800191 | 0.029261502 | 2.245167 | down | adenosinedeaminase-like |
| 17084904 | 3.66E-04 | 2.2446377 | down | maternalembryonicleucinezipperkinase |
| 17087025 | 0.009050748 | 2.2446182 | down |  |
| 17013468 | 0.011594151 | 2.2329533 | down | syntaxinbindingprotein5(tomosyn) |
| 16777896 | 0.019701023 | 2.2306962 | down | heatshock105kDa/110kDaprotein1 |
| 17055459 | 0.03154021 | 2.2261777 | down | isoprenoidsynthasedomaincontaining |
| 16795508 | 4.87E-04 | 2.2246435 | down | galactosylceramidase |
| 17087034 | 0.004042058 | 2.2194748 | down | hippocampusabundanttranscript-like1 |
| 16868576 | 0.009649146 | 2.2194548 | down | DNA(cytosine-5-)-methyltransferase1 |
| 17069728 | 0.019191697 | 2.216775 | down | phosphatidylinositol-3,4,5-trisphosphate-dependentRacexchangefactor2 |
| 16833292 | 0.044706944 | 2.2123697 | down |  |
| 16705159 | 0.002523817 | 2.2107253 | down | cyclin-dependentkinase1 |
| 17111219 | 0.007612026 | 2.2085154 | down | structuralmaintenanceofchromosomes1A |
| 16857913 | 0.003820824 | 2.2065864 | down | heterogeneousnuclearribonucleoproteinM |
| 16824690 | 0.00739131 | 2.2013633 | down | ERI1exoribonucleasefamilymember2 |
| 16715048 | 0.032997854 | 2.2012017 | down |  |
| 16759202 | 0.001260897 | 2.2001562 | down |  |
| 17062906 | 0.009333215 | 2.1992426 | down | coatomerproteincomplex,subunitgamma2 |
| 16787208 | 0.001078056 | 2.1964273 | down | Gprotein-coupledreceptor65 |
| 16879116 | 0.006556647 | 2.1953976 | down |  |
| 16839104 | 0.04351486 | 2.1922379 | down |  |
| 16779085 | 0.013551238 | 2.1915889 | down | regulatorofchromosomecondensation(RCC1)andBTB(POZ)domaincontainingprotein1 |
| 17072144 | 0.007467334 | 2.1884143 | down | DEPdomaincontainingMTOR-interactingprotein |
| 17023308 | 0.004978441 | 2.188029 | down | HDdomaincontaining2 |
| 16970231 | 7.81E-05 | 2.185665 | down | exosomecomponent9 |
| 16777278 | 0.007075843 | 2.184011 | down | spindleandkinetochoreassociatedcomplexsubunit3 |
| 16753914 | 0.024374148 | 2.1827815 | down | lysozyme |
| 16993268 | 0.0016073 | 2.1801476 | down | heterogeneousnuclearribonucleoproteinA/B |
| 16825104 | 0.007580433 | 2.1801414 | down | partnerandlocalizerofBRCA2 |
| 16914671 | 2.51E-04 | 2.1790037 | down | CSE1chromosomesegregation1-like(yeast) |
| 16708552 | 0.00617908 | 2.177956 | down | nucleolarandcoiled-bodyphosphoprotein1 |
| 16851604 | 0.00245606 | 2.175405 | down | Impacthomolog(mouse) |
| 16755498 | 7.79E-04 | 2.1747508 | down | thymopoietin |
| 16958303 | 0.001345513 | 2.1742275 | down | coiled-coildomaincontaining14 |
| 17009862 | 0.004092765 | 2.1718218 | down | primase,DNA,polypeptide2(58kDa)\|uncharacterizedLOC100653079 |
| 17051236 | 0.012036863 | 2.1716282 | down | calumenin |
| 17057718 | 0.0029719 | 2.171404 | down | fidgetin-like1 |
| 16811073 | 2.87E-04 | 2.1709566 | down | ceroid-lipofuscinosis,neuronal6,lateinfantile,variant |
| 16939960 | 0.006610859 | 2.167178 | down | kinesinfamilymember15 |
| 16696533 | 0.02655376 | 2.1640341 | down | smallnucleolarRNA,C/Dbox75 |
| 17007851 | 0.028138703 | 2.1638403 | down |  |
| 16824566 | 0.012599992 | 2.1593812 | down | chromosome16openreadingframe88 |
| 16957565 | 0.04937752 | 2.1508095 | down |  |
| 16983765 | 0.001872101 | 2.1459968 | down | natriureticpeptidereceptorC/guanylatecyclaseC(atrionatriureticpeptidereceptorC) |
| 17092020 | 0.009580787 | 2.14137 | down |  |
| 16988801 | 0.011716338 | 2.1399379 | down | solutecarrierfamily12(sodium/potassium/chloridetransporters),member2 |
| 16836788 | 0.013854503 | 2.1378279 | down | methyltransferaselike2A |
| 17109211 | 0.004481065 | 2.133993 | down | Fanconianemia,complementationgroupB |
| 17011683 | 0.014188803 | 2.1339605 | down | generaltranscriptionfactorIIIC,polypeptide6,alpha35kDa |
| 16785483 | 1.85E-04 | 2.1334927 | down | fucosyltransferase8(alpha(1,6)fucosyltransferase) |
| 17107045 | 0.029750293 | 2.1323197 | down | hypoxanthinephosphoribosyltransferase1 |
| 17056358 | 8.85E-04 | 2.1240346 | down | gamma-glutamylcyclotransferase |
| 16849379 | 0.003404581 | 2.1227274 | down | thymidinekinase1,soluble |
| 16984347 | 0.028929703 | 2.1208189 | down | F-boxprotein4 |
| 16743111 | 0.009445898 | 2.11944 | down | cathepsinC |
| 16802162 | 0.006982395 | 2.1188204 | down | DIS3mitoticcontrolhomolog(S.cerevisiae)-like |
| 16711998 | 0.024909634 | 2.1185372 | down |  |
| 16832768 | 6.94E-04 | 2.1166885 | down | carboxypeptidaseD |
| 16731169 | 7.90E-04 | 2.115758 | down | dihydrolipoamideS-acetyltransferase |
| 16884162 | 0.006466331 | 2.1143417 | down | coiled-coildomaincontaining138 |
| 16816102 | 1.03E-04 | 2.1132264 | down | NODALmodulator1\|NODALmodulator2 |
| 17080749 | 4.46E-04 | 2.112662 | down | ATPasefamily,AAAdomaincontaining2 |
| 17118315 | 0.03214265 | 2.1126218 | down |  |
| 16958812 | 0.002944515 | 2.1121905 | down | RuvB-like1(E.coli) |
| 16894740 | 0.001536788 | 2.11187 | down | structuralmaintenanceofchromosomes6 |
| 17010991 | 0.003909502 | 2.1111257 | down | caspase8associatedprotein2 |
| 17005396 | 7.00E-04 | 2.1100218 | down | geminin,DNAreplicationinhibitor |
| 16761923 | 0.04546773 | 2.1094887 | down |  |
| 16772234 | 7.42E-04 | 2.1075242 | down |  |
| 16682425 | 0.001769609 | 2.1030903 | down | regulatorofchromosomecondensation2 |
| 16774855 | 0.024319347 | 2.103066 | down |  |
| 16707466 | 0.023469325 | 2.0994954 | down |  |
| 17016363 | 0.012972382 | 2.0993538 | down | histonecluster1,H3b\|histonecluster1,H3f\|histonecluster1,H3h\|histonecluster1,H3j\|histonecluster1,H3g\|histonecluster1,H3i\|histonecluster1,H3e\|histonecluster1,H3c\|histonecluster1,H3d\|histonecluster1,H3a |
| 16832405 | 0.003894708 | 2.0988252 | down | nemo-likekinase |
| 16684406 | 0.003479305 | 2.098763 | down | smallnuclearribonucleoprotein40kDa(U5) |
| 16861047 | 0.003443239 | 2.0964613 | down | transmembraneprotein147 |
| 16875034 | 0.04544183 | 2.0929148 | down | zincfingerprotein836 |
| 16985033 | 0.005882106 | 2.0907621 | down | GRB2-bindingadaptorprotein,transmembrane |
| 16725160 | 0.023418576 | 2.0887868 | down | membrane-spanning4-domains,subfamilyA,member3(hematopoieticcell-specific) |
| 17011079 | 0.016177742 | 2.087165 | down | mannosidase,endo-alpha |
| 17114567 | 0.005010841 | 2.0842392 | down | smallnucleolarRNA,C/Dbox61\|RNAbindingmotifprotein,X-linked |
| 16766137 | 0.026650371 | 2.0826633 | down | timelesshomolog(Drosophila) |
| 16742610 | 0.038329914 | 2.0825176 | down | asparagine-linkedglycosylation8,alpha-1,3-glucosyltransferasehomolog(S.cerevisiae) |
| 16798919 | 0.018760577 | 2.0810754 | down | RhoGTPaseactivatingprotein11A |
| 17089898 | 0.007664937 | 2.0808141 | down | nucleoporin188kDa |
| 16897560 | 0.02510344 | 2.0803816 | down | proteasome(prosome,macropain)activatorsubunit4 |
| 17079293 | 0.029125484 | 2.0790763 | down | cyclinE2 |
| 16890490 | 0.009142871 | 2.078342 | down | 5-aminoimidazole-4-carboxamideribonucleotideformyltransferase/IMPcyclohydrolase |
| 16697463 | 0.020129623 | 2.0777216 | down | glutaredoxin2 |
| 16948278 | 2.44E-04 | 2.076662 | down | actin-like6A |
| 16696425 | 0.00190399 | 2.076056 | down | tumornecrosisfactor(ligand)superfamily,member4 |
| 16819181 | 0.012050883 | 2.0744345 | down | 2-oxoglutarateandiron-dependentoxygenasedomaincontaining1 |
| 16904780 | 0.005561572 | 2.0718539 | down |  |
| 16959187 | 0.025866123 | 2.0661666 | down | mitochondrialribosomalproteinL3 |
| 17071208 | 0.002528607 | 2.0628986 | down | lysosomalproteintransmembrane4beta |
| 16671139 | 0.033505436 | 2.062367 | down | S100calciumbindingproteinA9 |
| 16820584 | 0.011167905 | 2.0591505 | down | nuclearimport7homolog(S.cerevisiae) |
| 16738869 | 2.61E-04 | 2.059041 | down | PRP19/PSO4pre-mRNAprocessingfactor19homolog(S.cerevisiae) |
| 16852445 | 0.00880336 | 2.058254 | down | chromosome18openreadingframe54 |
| 16971139 | 0.02000919 | 2.0569975 | down | ATP-bindingcassette,sub-familyE(OABP),member1 |
| 17087588 | 0.012700331 | 2.0552375 | down | transmembraneproteinwithEGF-likeandtwofollistatin-likedomains1\|MSANTD3-TMEFF1readthrough\|Myb/SANT-likeDNA-bindingdomaincontaining3 |
| 16667175 | 0.016391449 | 2.054636 | down | smallnucleolarRNA,H/ACAbox66 |
| 16970409 | 0.002712406 | 2.0543494 | down | spermatogenesisassociated5 |
| 16860508 | 0.001634576 | 2.0535562 | down | dpy-19-like3(C.elegans) |
| 16826767 | 0.026191724 | 2.052122 | down | cytokineinducedapoptosisinhibitor1 |
| 16843627 | 0.01451504 | 2.0516186 | down | chemokine(C-Cmotif)ligand3-like3\|chemokine(C-Cmotif)ligand3-like1 |
| 16957568 | 0.01456575 | 2.0515773 | down | N(alpha)-acetyltransferase50,NatEcatalyticsubunit |
| 16792810 | 0.00122329 | 2.0441685 | down | ninein(GSK3Binteractingprotein) |
| 17019425 | 0.002504741 | 2.04407 | down | exportin5 |
| 16701287 | 0.002388465 | 2.0427852 | down | adenylosuccinatesynthase |
| 16904741 | 0.016567506 | 2.0423985 | down | serinethreoninekinase39 |
| 16709317 | 0.041525 | 2.0403442 | down | microRNA4295 |
| 16770040 | 0.005124732 | 2.0402017 | down | anaphasepromotingcomplexsubunit7 |
| 16882975 | 0.011196089 | 2.039709 | down | non-SMCcondensinIcomplex,subunitH |
| 16801993 | 0.011891091 | 2.0388472 | down |  |
| 16674767 | 0.012511536 | 2.037873 | down | DEAH(Asp-Glu-Ala-His)boxpolypeptide9 |
| 16947235 | 0.025623787 | 2.0352867 | down | guaninemonphosphatesynthetase |
| 16851344 | 4.48E-04 | 2.0344908 | down | smallnuclearribonucleoproteinD1polypeptide16kDa |
| 16794604 | 0.04014934 | 2.0342686 | down |  |
| 16962523 | 1.02E-05 | 2.0336022 | down | ribosomalproteinL39-like |
| 16901957 | 0.00779359 | 2.0324707 | down | cytoskeletonassociatedprotein2-like |
| 16701689 | 0.002769205 | 2.0311415 | down |  |
| 16838103 | 0.001161909 | 2.030324 | down | smallnucleolarRNA,C/Dbox1A |
| 17048382 | 5.53E-04 | 2.0301802 | down |  |
| 16833545 | 0.02250641 | 2.0274444 | down | transcriptionaladaptor2A |
| 16919906 | 6.48E-04 | 2.0270817 | down | TP53regulatingkinase |
| 16810543 | 0.024583204 | 2.0250137 | down |  |
| 16911147 | 0.008515629 | 2.024054 | down |  |
| 16983172 | 1.61E-04 | 2.0236568 | down | chaperonincontainingTCP1,subunit5(epsilon) |
| 16934302 | 0.037290458 | 2.0234628 | down |  |
| 16834486 | 0.011612067 | 2.0216963 | down | proteasome(prosome,macropain)activatorsubunit3(PA28gamma |
| 16883715 | 0.0127027 | 2.0216544 | down | interleukin18receptor1 |
| 17101248 | 0.027855584 | 2.0147345 | down | ARSDantisenseRNA1(non-proteincoding) |
| 16824082 | 0.045272514 | 2.013238 | down | poly(A)-specificribonuclease |
| 16739636 | 0.006453318 | 2.0123253 | down | smallnucleolarRNAhostgene1(non-proteincoding) |
| 16878137 | 0.00139665 | 2.0118258 | down | carbamoyl-phosphatesynthetase2,aspartatetranscarbamylase,anddihydroorotase |
| 16831429 | 0.004912291 | 2.011132 | down |  |
| 16787669 | 0.002220792 | 2.0101898 | down | chromosome14openreadingframe109 |
| 16816604 | 0.047543593 | 2.009511 | down | acyl-CoAsynthetasemedium-chainfamilymember3 |
| 16868443 | 0.02958411 | 2.0092585 | down | zincfingerprotein562 |
| 17012379 | 0.018427975 | 2.0084507 | down | centromereproteinW |
| 17106031 | 0.005903781 | 2.0066295 | down | phosphoribosylpyrophosphatesynthetase1 |
| 16684175 | 0.004375166 | 2.004808 | down | smallnucleolarRNAhostgene3(non-proteincoding)\|smallnucleolarRNA,H/ACAbox73A |
| 16869588 | 0.001125971 | 2.0045118 | down | ASF1anti-silencingfunction1homologB(S.cerevisiae) |
| 16709713 | 0.011588328 | 2.0026255 | down | solutecarrierfamily18(vesicularmonoamine),member2 |
| 16845742 | 0.016309863 | 2.0014927 | down | elongationfactorTuGTPbindingdomaincontaining2 |
| 16801473 | 0.048108965 | 1.9993985 | down | aquaporin9 |
| 16705474 | 0.001886424 | 1.998862 | down | DEAD(Asp-Glu-Ala-Asp)boxhelicase21 |
| 16702503 | 0.03064909 | 1.9974359 | down | celldivisioncycle123homolog(S.cerevisiae) |
| 17012859 | 0.004079247 | 1.9955454 | down | phosphodiesterase7B |
| 16981405 | 0.02460096 | 1.9949158 | down | chromosome4openreadingframe27 |
| 16836457 | 0.004524804 | 1.9946433 | down | RAD51homologC(S.cerevisiae) |
| 16843602 | 0.018245768 | 1.9902468 | down | chemokine(C-Cmotif)ligand3-like3\|chemokine(C-Cmotif)ligand3-like1 |
| 16775014 | 0.001200468 | 1.990173 | down | cytoskeletonassociatedprotein2 |
| 16808563 | 0.030130163 | 1.9883183 | down |  |
| 17020044 | 0.04566823 | 1.9882332 | down | translocationassociatedmembraneprotein2 |
| 16957951 | 0.006266971 | 1.9873588 | down | polymerase(DNAdirected),theta |
| 16966855 | 0.006933229 | 1.9854954 | down | v-kitHardy-Zuckerman4felinesarcomaviraloncogenehomolog |
| 16924966 | 0.003604138 | 1.9845818 | down | MIS18kinetochoreproteinhomologA(S.pombe) |
| 16806536 | 0.0333759 | 1.9819893 | down |  |
| 16787679 | 0.026087454 | 1.9792541 | down | ubiquitinproteinligaseE3componentn-recognin7(putative) |
| 16843829 | 0.041365165 | 1.9786167 | down | DEAD(Asp-Glu-Ala-Asp)boxpolypeptide52 |
| 17096904 | 3.02E-04 | 1.9784358 | down | catenin(cadherin-associatedprotein),alpha-like1 |
| 16973879 | 0.016591404 | 1.9781913 | down | Ly1antibodyreactivehomolog(mouse) |
| 16798763 | 0.03390902 | 1.9775456 | down |  |
| 16981502 | 0.03296987 | 1.9767469 | down |  |
| 16736726 | 0.008814124 | 1.976014 | down | smallVCP/p97-interactingprotein |
| 16871613 | 0.003915404 | 1.9703704 | down | polymerase(RNA)II(DNAdirected)polypeptideI,14.5kDa |
| 17012844 | 0.00280359 | 1.9701978 | down |  |
| 16784272 | 0.001597842 | 1.9694935 | down |  |
| 16780069 | 0.003680397 | 1.9676204 | down | sproutyhomolog2(Drosophila) |
| 17010941 | 0.029055785 | 1.9669813 | down | peptidaseM20domaincontaining2 |
| 16692636 | 0.007713875 | 1.9667594 | down | histonecluster2,H2ab |
| 16952451 | 2.40E-04 | 1.9665378 | down |  |
| 16870808 | 0.042966247 | 1.9657701 | down |  |
| 16697544 | 2.45E-04 | 1.9649352 | down | asp(abnormalspindle)homolog,microcephalyassociated(Drosophila) |
| 16850426 | 0.003911812 | 1.9619725 | down |  |
| 16826160 | 6.10E-05 | 1.9608618 | down | SHCSH2-domainbindingprotein1 |
| 16702685 | 0.001914533 | 1.9545475 | down | suppressorofvariegation3-9homolog2(Drosophila) |
| 16721807 | 0.005084644 | 1.9538381 | down | smallnucleolarRNA,H/ACAbox23 |
| 16711020 | 0.04885891 | 1.952642 | down |  |
| 16765878 | 0.003027892 | 1.9525965 | down |  |
| 16979825 | 0.001681943 | 1.9509419 | down | poly(A)bindingprotein,cytoplasmic4-like |
| 17007634 | 0.012896612 | 1.9500893 | down |  |
| 16712576 | 0.007279797 | 1.9495028 | down | phosphoribosyltransferasedomaincontaining1 |
| 16969093 | 0.005537805 | 1.9493624 | down | RAP1,GTP-GDPdissociationstimulator1 |
| 16716870 | 0.001593295 | 1.9481964 | down | tectonicfamilymember3 |
| 17102041 | 0.003844801 | 1.9481816 | down | sperminesynthase |
| 16984080 | 0.019408219 | 1.9478835 | down |  |
| 16686324 | 9.21E-04 | 1.9469011 | down | eukaryotictranslationinitiationfactor2B,subunit3gamma,58kDa |
| 16883426 | 6.60E-04 | 1.9458104 | down | eukaryotictranslationinitiationfactor5B |
| 17074259 | 0.027684504 | 1.9442763 | down | angiopoietin2 |
| 17046254 | 7.80E-04 | 1.9441634 | down | chaperonincontainingTCP1,subunit6A(zeta1) |
| 17078558 | 0.037749957 | 1.9436396 | down | phosphoproteinassociatedwithglycosphingolipidmicrodomains1 |
| 17061106 | 0.035334367 | 1.9434686 | down | RASp21proteinactivator4\|RASp21proteinactivator4B\|RASp21proteinactivator4C,pseudogene\|uroplakin3B-like |
| 16902357 | 0.001688775 | 1.9427085 | down | MKI67(FHAdomain)interactingnucleolarphosphoprotein |
| 16879923 | 4.58E-04 | 1.9404116 | down | mutShomolog6(E.coli) |
| 16838787 | 2.02E-04 | 1.9384658 | down | chromosome17openreadingframe89 |
| 17023551 | 0.013225982 | 1.9384313 | down | erythrocytemembraneproteinband4.1-like2 |
| 16995409 | 1.91E-04 | 1.9372007 | down | nucleoporin155kDa |
| 16991669 | 0.004885989 | 1.9370893 | down | ubiquitin-likedomaincontainingCTDphosphatase1 |
| 16734163 | 1.91E-04 | 1.9349263 | down | chitinasedomaincontaining1 |
| 16760028 | 0.004897341 | 1.9346412 | down |  |
| 16891774 | 0.003619359 | 1.9336592 | down | chemokine(C-Cmotif)ligand20 |
| 16663155 | 0.012178161 | 1.9320676 | down | CTPsynthase1 |
| 16898533 | 0.015924845 | 1.9319646 | down | cannabinoidreceptorinteractingprotein1 |
| 17099083 | 0.002094242 | 1.9304721 | down | torsinfamily1,memberA(torsinA) |
| 17112835 | 0.00401188 | 1.9301695 | down | zincfinger,matrin-type1 |
| 17077525 | 0.002789897 | 1.9299971 | down | carbonicanhydraseVIII |
| 17108500 | 0.009409712 | 1.9296968 | down | dyskeratosiscongenita1,dyskerin\|smallnucleolarRNA,H/ACAbox56 |
| 16894848 | 0.010067547 | 1.929351 | down | WDrepeatdomain35 |
| 16907548 | 0.018096212 | 1.9271593 | down | NADHdehydrogenase(ubiquinone)Fe-Sprotein1,75kDa(NADH-coenzymeQreductase) |
| 16882942 | 0.02161167 | 1.9265891 | down |  |
| 17005603 | 0.02839489 | 1.9261018 | down | histonecluster1,H2bc\|histonecluster1,H2bi\|histonecluster1,H2be\|histonecluster1,H2bf\|histonecluster1,H2bg |
| 17016473 | 0.028527508 | 1.9258875 | down | zincfingerprotein184 |
| 16736807 | 0.015053627 | 1.9255825 | down | coiled-coildomaincontaining34 |
| 16698286 | 0.027310293 | 1.9238813 | down | renin |
| 16669288 | 0.003060258 | 1.9232099 | down | WDrepeatdomain3 |
| 16975954 | 0.009362876 | 1.922629 | down | sec1familydomaincontaining2 |
| 16838249 | 0.013147316 | 1.9220263 | down |  |
| 16665447 | 0.019220643 | 1.9204208 | down | ubiquitinspecificpeptidase1 |
| 17117581 | 0.006262927 | 1.9181494 | down | ACAH3104 |
| 17111008 | 0.025755407 | 1.9178603 | down | nudix(nucleosidediphosphatelinkedmoietyX)-typemotif11 |
| 16713572 | 0.007418475 | 1.9163681 | down | chromosome10openreadingframe25 |
| 16739317 | 6.29E-04 | 1.9159203 | down | metastasisassociated1family,member2 |
| 17059756 | 0.00162122 | 1.9158523 | down | cyclin-dependentkinase6 |
| 16958506 | 0.032377668 | 1.9138925 | down | oxysterolbindingprotein-like11 |
| 16685839 | 0.0494079 | 1.9128873 | down |  |
| 16819269 | 0.00720925 | 1.9127815 | down | nucleoporin93kDa |
| 16829764 | 0.032561 | 1.9127718 | down | germcellassociated2(haspin) |
| 17109808 | 0.002119474 | 1.9117151 | down | apolipoproteinO |
| 16729557 | 0.00379037 | 1.9111419 | down | chromosome11openreadingframe82 |
| 16668960 | 0.013374357 | 1.9096122 | down | DNAcross-linkrepair1B |
| 16970937 | 2.35E-04 | 1.9092891 | down | ELMO/CED-12domaincontaining2 |
| 16937503 | 4.04E-04 | 1.9084064 | down |  |
| 17059974 | 0.00901764 | 1.9066892 | down | solutecarrierfamily25(aspartate/glutamatecarrier),member13 |
| 16712277 | 0.00165241 | 1.9066888 | down | ST8alpha-N-acetyl-neuraminidealpha-2,8-sialyltransferase6 |
| 17009482 | 0.022697529 | 1.9057846 | down | centromereproteinQ |
| 16707872 | 0.027782403 | 1.9057492 | down | cyclinJ |
| 16711847 | 0.011809266 | 1.9056909 | down | phytanoyl-CoA2-hydroxylase |
| 16665512 | 0.025771616 | 1.9054552 | down | asparagine-linkedglycosylation6,alpha-1,3-glucosyltransferasehomolog(S.cerevisiae) |
| 17085672 | 0.002290055 | 1.905319 | down | frataxin |
| 16780640 | 0.001791777 | 1.9040052 | down |  |
| 16959835 | 0.010830121 | 1.9027587 | down |  |
| 16942991 | 0.016636604 | 1.9021708 | down | ADP-ribosylationfactor-like13B |
| 16748620 | 0.001179218 | 1.900236 | down | activatingtranscriptionfactor7interactingprotein |
| 17080082 | 0.004739525 | 1.90007 | down | angiopoietin1 |
| 17079315 | 3.46E-04 | 1.8998034 | down |  |
| 16708802 | 0.027336258 | 1.8992178 | down |  |
| 16977016 | 0.005680719 | 1.89881 | down | SDA1domaincontaining1 |
| 17084990 | 0.020021526 | 1.8979304 | down | polymerase(RNA)IpolypeptideE,53kDa |
| 16841885 | 0.04682457 | 1.8971491 | down | COP9constitutivephotomorphogenichomologsubunit3(Arabidopsis) |
| 16721773 | 0.013156021 | 1.8969775 | down | importin7 |
| 16808433 | 2.23E-04 | 1.8959597 | down | protein(peptidylprolylcis/transisomerase)NIMA-interacting,4pseudogene1 |
| 17013065 | 0.014559749 | 1.8951552 | down | ABRAC-terminallike |
| 16706499 | 0.02688296 | 1.8949577 | down | peptidylprolylisomeraseF |
| 16791300 | 1.11E-04 | 1.894559 | down | celldeath-inducingDFFA-likeeffectorb |
| 16947556 | 1.73E-04 | 1.8941689 | down | structuralmaintenanceofchromosomes4 |
| 16940396 | 0.014249593 | 1.8936536 | down |  |
| 17000129 | 0.001866399 | 1.8920889 | down | H2Ahistonefamily,memberY |
| 16912192 | 0.004085985 | 1.8916037 | down | GINScomplexsubunit1(Psf1homolog) |
| 16852206 | 0.007077541 | 1.8908534 | down | HAUSaugmin-likecomplex,subunit1 |
| 16679981 | 8.28E-04 | 1.8905095 | down |  |
| 16669212 | 0.006216742 | 1.889025 | down | transcriptionterminationfactor,RNApolymeraseII |
| 16922584 | 4.19E-04 | 1.8884544 | down | chromatinassemblyfactor1,subunitB(p60) |
| 16924276 | 0.047693785 | 1.8864878 | down |  |
| 16777502 | 0.007888608 | 1.8857138 | down | centromereproteinJ |
| 17100614 | 0.005366862 | 1.8847908 | down |  |
| 16924878 | 4.08E-05 | 1.8839805 | down | T-celllymphomainvasionandmetastasis1 |
| 16989241 | 0.032329954 | 1.8822405 | down | microRNA3661 |
| 16766348 | 0.021019232 | 1.8821731 | down | zincfingerandBTBdomaincontaining39 |
| 16880442 | 0.00264615 | 1.8821063 | down |  |
| 16925674 | 0.006018102 | 1.8820225 | down | proteasome(prosome,macropain)assemblychaperone1 |
| 16898498 | 4.37E-04 | 1.8798473 | down | WDrepeatdomain92\|proteinphosphatase3,regulatorysubunitB,alpha |
| 17094028 | 0.003242978 | 1.8780088 | down | translocaseofoutermitochondrialmembrane5homolog(yeast) |
| 17082032 | 0.03485765 | 1.8779012 | down |  |
| 16836492 | 0.016869385 | 1.8771651 | down | prolinerich11 |
| 16814498 | 0.014115825 | 1.8770937 | down | meteorin,glialcelldifferentiationregulator |
| 16793052 | 0.005888259 | 1.8768935 | down | glucosamine-phosphateN-acetyltransferase1 |
| 16712531 | 0.004291175 | 1.8744674 | down | RhoGTPaseactivatingprotein21 |
| 17076804 | 0.003765598 | 1.8741821 | down |  |
| 16973578 | 0.002567649 | 1.8737472 | down | stem-loopbindingprotein |
| 16938899 | 9.72E-04 | 1.8734907 | down | mutLhomolog1,coloncancer,nonpolyposistype2(E.coli) |
| 16878556 | 0.014904555 | 1.8733202 | down | WDrepeatdomain43 |
| 17112349 | 0.027371353 | 1.8720646 | down | cysteinylleukotrienereceptor1 |
| 16668002 | 0.005874176 | 1.8705074 | down | familywithsequencesimilarity102,memberB |
| 16773507 | 0.002934202 | 1.8695835 | down | generaltranscriptionfactorIIIA |
| 16907546 | 0.006983056 | 1.8692828 | down | glycinecleavagesystemproteinH(aminomethylcarrier)pseudogene3 |
| 16961308 | 0.001795702 | 1.8689585 | down | golgiintegralmembraneprotein4 |
| 17064459 | 0.002137884 | 1.8687936 | down | ATP-bindingcassette,sub-familyF(GCN20),member2 |
| 17086784 | 0.02391516 | 1.8681128 | down | centromereproteinP |
| 16779119 | 0.001889953 | 1.8679074 | down | karyopherinalpha3(importinalpha4) |
| 16753800 | 0.005040931 | 1.866844 | down | nucleoporin107kDa |
| 16986748 | 0.005294134 | 1.8661691 | down | mutShomolog3(E.coli) |
| 17118019 | 0.024915164 | 1.8661302 | down |  |
| 17022849 | 0.03542691 | 1.866105 | down |  |
| 16687418 | 0.022767138 | 1.8659719 | down | transmembraneprotein48 |
| 16967135 | 0.037399255 | 1.8649575 | down |  |
| 16693409 | 0.007991258 | 1.8645226 | down | S100calciumbindingproteinA12 |
| 16820537 | 0.002851918 | 1.8638684 | down | cirrhosis,autosomalrecessive1A(cirhin) |
| 16718983 | 1.27E-04 | 1.8636537 | down | minichromosomemaintenancecomplexbindingprotein |
| 17118305 | 0.013811166 | 1.8622986 | down |  |
| 17114220 | 0.001081683 | 1.8621771 | down | muscleblind-likesplicingregulator3 |
| 16897631 | 0.041330267 | 1.8590853 | down |  |
| 16979482 | 0.001645918 | 1.8588713 | down | annexinA5 |
| 17100689 | 0.025032012 | 1.858699 | down |  |
| 16722252 | 0.00517907 | 1.857842 | down | fattyacylCoAreductase1 |
| 16993506 | 0.011828338 | 1.8565438 | down | CCR4-NOTtranscriptioncomplex,subunit6 |
| 16907257 | 0.005561825 | 1.85623 | down | traffickingprotein,kinesinbinding2 |
| 16969131 | 0.047170695 | 1.8552779 | down | methionylaminopeptidase1 |
| 16934671 | 0.012313026 | 1.8551188 | down | thiosulfatesulfurtransferase(rhodanese) |
| 16855184 | 0.010064416 | 1.8545445 | down | acetyl-CoAacyltransferase2 |
| 17113565 | 0.005001715 | 1.8544879 | down |  |
| 16901986 | 0.00673878 | 1.8541867 | down | interleukin1,beta |
| 16871718 | 0.005316232 | 1.8528781 | down | zincfingerprotein850 |
| 16955133 | 0.011699938 | 1.8526287 | down | ARP8actin-relatedprotein8homolog(yeast) |
| 17005234 | 0.005746258 | 1.8523096 | down | E2Ftranscriptionfactor3 |
| 16820787 | 0.001158763 | 1.8522328 | down | splicingfactor3b,subunit3,130kDa |
| 16826243 | 0.004838975 | 1.8518605 | down | integrinalphaFG-GAPrepeatcontaining1 |
| 17035225 | 0.012743269 | 1.8513514 | down | mediatorofDNA-damagecheckpoint1\|MDC1antisenseRNA1(non-proteincoding) |
| 16708815 | 0.011310459 | 1.850369 | down | programmedcelldeath11 |
| 16860103 | 0.003020942 | 1.8491609 | down | zincfingerprotein93 |
| 17106574 | 0.003586924 | 1.8489308 | down | solutecarrierfamily25(mitochondrialcarrier |
| 16963646 | 0.003015137 | 1.8486444 | down |  |
| 17040598 | 0.013374276 | 1.8485707 | down | mediatorofDNA-damagecheckpoint1 |
| 17087779 | 0.024702664 | 1.84854 | down | FP944 |
| 17117547 | 0.00991918 | 1.8483607 | down | IGYY565 |
| 16686796 | 0.009040366 | 1.8480175 | down | SCL/TAL1interruptinglocus |
| 16971308 | 0.002912197 | 1.8475592 | down | RhoGTPaseactivatingprotein10 |
| 17104122 | 0.029042391 | 1.847551 | down | melanomaantigenfamilyH,1 |
| 16724593 | 0.005917134 | 1.8473257 | down | proteintyrosinephosphatase,mitochondrial1\|NADHdehydrogenase(ubiquinone)Fe-Sprotein3,30kDa(NADH-coenzymeQreductase) |
| 16681082 | 0.014420007 | 1.8466833 | down | isoprenylcysteinecarboxylmethyltransferase |
| 16681661 | 1.18E-04 | 1.8466011 | down | mechanistictargetofrapamycin(serine/threoninekinase) |
| 16927174 | 0.009644983 | 1.8465738 | down | DiGeorgesyndromecriticalregiongene8\|microRNA1306\|microRNA3618 |
| 16807080 | 4.37E-04 | 1.846406 | down | ATPbindingdomain4 |
| 16869984 | 0.008484515 | 1.8452652 | down | epidermalgrowthfactorreceptorpathwaysubstrate15-like1 |
| 16944722 | 0.02783824 | 1.8450025 | down |  |
| 16674953 | 0.001804222 | 1.8449881 | down | tRNAsplicingendonuclease15homolog(S.cerevisiae) |
| 17072222 | 0.03764935 | 1.84421 | down |  |
| 17011895 | 0.001287228 | 1.8435271 | down | 5'-nucleotidasedomaincontaining1 |
| 16911675 | 0.014245736 | 1.8433791 | down | chromosome20openreadingframe72 |
| 17118323 | 0.008926874 | 1.8431975 | down |  |
| 16974779 | 9.07E-04 | 1.8422313 | down | Gprotein-coupledreceptor125 |
| 16699590 | 0.016839461 | 1.8419043 | down | TATAboxbindingprotein(TBP)-associatedfactor,RNApolymeraseI,A,48kDa |
| 16732676 | 0.021444531 | 1.8407512 | down | ubiquitinassociatedandSH3domaincontainingB |
| 16986895 | 0.027364494 | 1.8406515 | down | X-rayrepaircomplementingdefectiverepairinChinesehamstercells4 |
| 16892039 | 0.02203593 | 1.8398682 | down | proteasome(prosome,macropain)26Ssubunit,non-ATPase,1 |
| 16890602 | 0.022099113 | 1.8397107 | down | DKFZp434H1419 |
| 17117537 | 0.041483875 | 1.839244 | down |  |
| 16789524 | 0.00399285 | 1.8389602 | down |  |
| 17107293 | 0.001948359 | 1.8386184 | down | HIV-1Tatspecificfactor1 |
| 17027555 | 0.014225668 | 1.8386137 | down | mediatorofDNA-damagecheckpoint1 |
| 16816627 | 0.010164396 | 1.8378196 | down | exonucleaseNEF-sp |
| 17030400 | 0.014630609 | 1.8375275 | down | mediatorofDNA-damagecheckpoint1 |
| 16685544 | 0.02429309 | 1.8370321 | down | splicingfactor3a,subunit3,60kDa |
| 17006374 | 0.014042791 | 1.8368502 | down | MDC1antisenseRNA1(non-proteincoding) |
| 17033105 | 0.014470458 | 1.8368121 | down | mediatorofDNA-damagecheckpoint1 |
| 16673983 | 9.83E-04 | 1.8364971 | down | aspartyl-tRNAsynthetase2,mitochondrial |
| 16888106 | 0.007917114 | 1.8362675 | down | oxysterolbindingprotein-like6 |
| 16959112 | 0.004634822 | 1.836254 | down | phosphoinositide-3-kinase,regulatorysubunit4 |
| 16739247 | 0.02117051 | 1.8357496 | down |  |
| 16831338 | 0.03749941 | 1.8355187 | down | zincfingerprotein286A |
| 16946464 | 0.008082964 | 1.835473 | down | ATPase,Na+/K+transporting,beta3polypeptide |
| 17026207 | 0.014090708 | 1.8341572 | down | mediatorofDNA-damagecheckpoint1 |
| 16916946 | 0.025710428 | 1.8333759 | down | transmembraneprotein230 |
| 16738819 | 0.024565287 | 1.8316565 | down | membrane-spanning4-domains,subfamilyA,member6A |
| 16910501 | 0.005804677 | 1.8292456 | down | deoxythymidylatekinase(thymidylatekinase) |
| 17092955 | 0.003837563 | 1.828675 | down | caspaseactivityandapoptosisinhibitor1 |
| 16723464 | 0.006182946 | 1.828484 | down |  |
| 16746237 | 0.006416858 | 1.8283908 | down | sortingnexin19 |
| 16664865 | 9.30E-04 | 1.8262309 | down |  |
| 16701748 | 0.002272936 | 1.8256477 | down | GTPbindingprotein4 |
| 17074125 | 0.006539941 | 1.8237469 | down |  |
| 16970863 | 0.002674487 | 1.8218112 | down | N(alpha)-acetyltransferase15,NatAauxiliarysubunit |
| 16825410 | 0.007656544 | 1.8209144 | down | Tutranslationelongationfactor,mitochondrial\|microRNA4721 |
| 16773346 | 0.03336187 | 1.8207495 | down | myotubularinrelatedprotein6 |
| 16812738 | 0.008191926 | 1.8201782 | down | hepatoma-derivedgrowthfactor,relatedprotein3 |
| 16811975 | 0.02494698 | 1.8191985 | down | tetraspanin3 |
| 17114014 | 0.021614067 | 1.8170474 | down |  |
| 16776905 | 2.15E-05 | 1.8166367 | down | transcriptionfactorDp-1 |
| 16685845 | 0.023027869 | 1.8165877 | down |  |
| 16804631 | 0.009060725 | 1.8152854 | down | chromosome15openreadingframe42 |
| 16745798 | 0.013273485 | 1.8149117 | down | fasciculationandelongationproteinzeta1(zyginI) |
| 16718870 | 0.003777007 | 1.8146455 | down | eukaryotictranslationinitiationfactor3,subunitA |
| 16711723 | 0.0318649 | 1.8142104 | down |  |
| 16907303 | 0.017796986 | 1.8142066 | down | transmembraneprotein237 |
| 16907458 | 0.012531204 | 1.8140569 | down | WDrepeatdomain12 |
| 16910825 | 4.61E-04 | 1.8131938 | down | NOP56ribonucleoproteinhomolog(yeast)\|smallnucleolarRNA,C/Dbox86\|microRNA1292\|smallnucleolarRNA,C/Dbox110\|smallnucleolarRNA,C/Dbox57 |
| 16876881 | 0.04713564 | 1.8124487 | down | cleavageandpolyadenylationspecificfactor3,73kDa |
| 17115677 | 0.005666208 | 1.8121638 | down | GRB2-associatedbindingprotein3 |
| 16667173 | 0.027152497 | 1.8117899 | down |  |
| 16718766 | 0.001119853 | 1.811449 | down |  |
| 16674319 | 0.008646091 | 1.8109967 | down | familywithsequencesimilarity20,memberB |
| 16968878 | 0.017376265 | 1.8100733 | down | multimerin1 |
| 16830103 | 0.023780279 | 1.8090496 | down | rabaptin,RABGTPasebindingeffectorprotein1 |
| 16828826 | 0.00994237 | 1.808784 | down | coactosin-like1(Dictyostelium) |
| 16833139 | 0.017988589 | 1.8087068 | down | proteasome(prosome,macropain)26Ssubunit,non-ATPase,11 |
| 16780358 | 0.003553375 | 1.8073603 | down | UDP-glucoseglycoproteinglucosyltransferase2 |
| 16774271 | 0.012961272 | 1.8068833 | down | N(alpha)-acetyltransferase16,NatAauxiliarysubunit |
| 16752586 | 0.004294699 | 1.8067219 | down | nucleicacidbindingprotein2 |
| 17071204 | 0.026388284 | 1.8050276 | down |  |
| 16746379 | 1.71E-04 | 1.8048179 | down | non-SMCcondensinIIcomplex,subunitD3 |
| 16882304 | 0.010928663 | 1.8045241 | down | ubiquitinspecificpeptidase39 |
| 16718022 | 0.021957867 | 1.8038776 | down | polycombgroupringfinger6 |
| 16689098 | 0.027978634 | 1.803772 | down | zincfinger,HIT-typecontaining6 |
| 17098871 | 0.030434817 | 1.8037364 | down |  |
| 16681192 | 0.023823852 | 1.8028178 | down | nucleolarprotein9 |
| 17102538 | 0.013624027 | 1.8025489 | down | cytochromeb-245,betapolypeptide |
| 16974581 | 0.014320711 | 1.8020943 | down |  |
| 16885432 | 0.006678271 | 1.8019105 | down | UDP-glucoseglycoproteinglucosyltransferase1 |
| 16678105 | 0.001980838 | 1.8017552 | down | signalrecognitionparticle9kDa |
| 17022562 | 0.007605685 | 1.8015068 | down | solutecarrierfamily22(organiccation/carnitinetransporter),member16 |
| 16985075 | 0.001715353 | 1.7998364 | down |  |
| 16840732 | 0.006451268 | 1.7990985 | down | tumorproteinp53 |
| 17114551 | 0.008274594 | 1.7988975 | down | RNAbindingmotifprotein,X-linked\|smallnucleolarRNA,C/Dbox61 |
| 16806823 | 0.02487654 | 1.7984091 | down | apoptosis,caspaseactivationinhibitor |
| 16931358 | 0.00496002 | 1.7980698 | down |  |
| 16899429 | 0.015718678 | 1.7976305 | down | GC-richsequenceDNA-bindingfactor2 |
| 16815807 | 0.007396538 | 1.7973071 | down | uncharacterizedLOC100287628\|activatingtranscriptionfactor7interactingprotein2 |
| 16708533 | 0.033060484 | 1.7971171 | down | peroxisomeproliferator-activatedreceptorgamma,coactivator-related1 |
| 16855539 | 0.00320235 | 1.7966998 | down |  |
| 16959325 | 0.003094778 | 1.796065 | down | topoisomerase(DNA)IIbindingprotein1 |
| 16699739 | 2.99E-04 | 1.7953373 | down | poly(ADP-ribose)polymerase1 |
| 16840284 | 0.008737931 | 1.7949693 | down | complementcomponent1,qsubcomponentbindingprotein |
| 17044193 | 0.027516961 | 1.7946373 | down |  |
| 16792712 | 0.01721604 | 1.7942597 | down |  |
| 16705377 | 0.005779336 | 1.7932135 | down | RNA,5Sribosomal319 |
| 17063073 | 0.002781543 | 1.7931641 | down | solutecarrierfamily35,memberB4 |
| 16790078 | 0.00102934 | 1.7925204 | down |  |
| 16807098 | 0.009704926 | 1.7922103 | down | microRNA3942 |
| 17057174 | 0.002854916 | 1.7914222 | down | polymerase(DNAdirected),delta2,accessorysubunit |
| 16980946 | 0.00947402 | 1.7907133 | down | plateletderivedgrowthfactorC |
| 16854842 | 0.025354553 | 1.789404 | down |  |
| 16826755 | 0.008815461 | 1.7883482 | down | uncharacterizedLOC100653273\|uncharacterizedLOC100652989 |
| 17107697 | 0.008616953 | 1.7878344 | down | myotubularin1 |
| 16838057 | 0.022948451 | 1.7878144 | down |  |
| 16992096 | 0.004474521 | 1.787456 | down | coiled-coildomaincontaining99 |
| 16683723 | 1.65E-04 | 1.7872849 | down | stathmin1 |
| 17064197 | 8.07E-04 | 1.7872694 | down | zincfingerfamilymember767 |
| 16805995 | 0.024665145 | 1.7870493 | down | nonimprintedinPrader-Willi/Angelmansyndrome1 |
| 16798810 | 0.025093617 | 1.7866604 | down |  |
| 16987720 | 0.017316736 | 1.7865107 | down | diphosphoinositolpentakisphosphatekinase2 |
| 17023924 | 0.018519077 | 1.7863402 | down | MYBantisenseRNA1(non-proteincoding) |
| 16799637 | 0.017142598 | 1.7861232 | down |  |
| 17058617 | 0.005351638 | 1.7843796 | down | replicationfactorC(activator1)2,40kDa |
| 16918976 | 0.007305995 | 1.7839737 | down |  |
| 17096242 | 0.024922902 | 1.783942 | down | AhpC/TSAantioxidantenzymedomaincontaining1 |
| 16840006 | 0.006295126 | 1.783241 | down | ubiquitin-conjugatingenzymeE2G1 |
| 16944894 | 0.035508413 | 1.7832386 | down | uridinemonophosphatesynthetase |
| 16966369 | 0.006747867 | 1.7820125 | down | transmembraneprotein33 |
| 16787279 | 0.048082575 | 1.7816215 | down | zincfingerCCCH-typecontaining14 |
| 16851121 | 0.029359909 | 1.781501 | down | SEH1-like(S.cerevisiae) |
| 16667546 | 0.033625487 | 1.7811571 | down | amylo-alpha-1,6-glucosidase,4-alpha-glucanotransferase |
| 16780441 | 0.001695029 | 1.780618 | down |  |
| 16816364 | 0.021876367 | 1.7803771 | down |  |
| 16675218 | 0.012352457 | 1.7802707 | down |  |
| 16726790 | 0.014876031 | 1.7794431 | down | polymerase(DNAdirected),alpha2,accessorysubunit |
| 16897026 | 0.01862643 | 1.7791013 | down | zincfingerprotein36,C3Htype-like2 |
| 16960271 | 0.018218206 | 1.7789066 | down | helicase-liketranscriptionfactor |
| 16757687 | 0.017714856 | 1.7774845 | down | replicationfactorC(activator1)5,36.5kDa |
| 17038267 | 0.04419457 | 1.7769704 | down | mutShomolog5(E.coli)\|MSH5-SAPCD1readthrough(non-proteincoding)\|suppressorAPCdomaincontaining1 |
| 16718903 | 0.023571867 | 1.7765063 | down | sideroflexin4 |
| 16948320 | 0.03490132 | 1.7759464 | down | ubiquitinspecificpeptidase13(isopeptidaseT-3) |
| 17016353 | 0.030432021 | 1.7754833 | down |  |
| 17022854 | 0.008150848 | 1.7748847 | down | histonedeacetylase2 |
| 16711866 | 5.18E-04 | 1.7747859 | down | selenophosphatesynthetase1 |
| 16658664 | 0.001015546 | 1.7741568 | down | solutecarrierfamily25(pyrimidinenucleotidecarrier),member33 |
| 16895848 | 0.001290911 | 1.7740852 | down | proteinphosphatase,Mg2+/Mn2+dependent,1G |
| 17010278 | 0.023958713 | 1.7726285 | down |  |
| 16837205 | 0.018271146 | 1.7722552 | down | nucleolarprotein11 |
| 16700934 | 0.008187593 | 1.7721288 | down | HEATrepeatcontaining1 |
| 16798733 | 0.009828839 | 1.7718785 | down | M-phasephosphoprotein10(U3smallnucleolarribonucleoprotein)pseudogene |
| 16662692 | 0.010767678 | 1.7707508 | down | UTP11-like,U3smallnucleolarribonucleoprotein,(yeast) |
| 16741287 | 0.003038822 | 1.7691904 | down | carnitinepalmitoyltransferase1A(liver) |
| 16999432 | 0.012536925 | 1.7690241 | down | chromosome5openreadingframe63 |
| 17078121 | 7.32E-05 | 1.768867 | down | lactamase,beta2 |
| 17015715 | 0.011934129 | 1.7683618 | down | TBC1domainfamily,member7 |
| 17062163 | 0.043357708 | 1.7678301 | down | cortactinbindingprotein2 |
| 16709732 | 0.01984909 | 1.7677982 | down |  |
| 16760668 | 0.007108271 | 1.7675092 | down | lysophosphatidylcholineacyltransferase3 |
| 16839345 | 0.044235323 | 1.7673672 | down | v-crksarcomavirusCT10oncogenehomolog(avian) |
| 16737783 | 4.16E-04 | 1.7663813 | down | cytoskeletonassociatedprotein5 |
| 16704844 | 0.019563159 | 1.7661791 | down | translocaseofinnermitochondrialmembrane23homolog(yeast) |
| 16847034 | 0.030732188 | 1.7655885 | down | myeloperoxidase |
| 16917030 | 0.002977647 | 1.7655077 | down | tRNAmethyltransferase6homolog(S.cerevisiae) |
| 16860175 | 0.041665252 | 1.7652755 | down | zincfingerprotein738 |
| 16699877 | 0.017326074 | 1.7643807 | down | laminBreceptor |
| 16902603 | 0.010383331 | 1.7641317 | down | polymerase(RNA)II(DNAdirected)polypeptideD |
| 17088576 | 5.17E-04 | 1.7638941 | down | UDP-GlcNAc:betaGalbeta-1,3-N-acetylglucosaminyltransferase5pseudogene |
| 16783905 | 0.005175051 | 1.7635244 | down | leucinerichrepeatprotein1 |
| 16842452 | 0.048656467 | 1.7635016 | down | ubiquitinspecificpeptidase22 |
| 16779701 | 1.28E-05 | 1.7632333 | down | dachshundhomolog1(Drosophila) |
| 16797751 | 0.01388793 | 1.7627629 | down | tubulin,gammacomplexassociatedprotein5 |
| 16775421 | 0.020922191 | 1.7626797 | down | ubiquitincarboxyl-terminalesteraseL3(ubiquitinthiolesterase) |
| 16773759 | 3.52E-04 | 1.7623099 | down | furryhomolog(Drosophila) |
| 17117588 | 0.001481524 | 1.7612869 | down | RANbindingprotein1pseudogene |
| 16816249 | 0.002115064 | 1.760511 | down | NODALmodulator3\|NODALmodulator1\|NODALmodulator2 |
| 16777384 | 0.001039032 | 1.7599866 | down | spasticataxiaofCharlevoix-Saguenay(sacsin) |
| 17094226 | 0.022542654 | 1.7587473 | down |  |
| 16676265 | 0.010555135 | 1.7574607 | down | smallnuclearribonucleoproteinpolypeptideE |
| 17033478 | 0.04926104 | 1.7570268 | down | mutShomolog5(E.coli)\|MSH5-SAPCD1readthrough(non-proteincoding)\|suppressorAPCdomaincontaining1 |
| 16890519 | 0.004869642 | 1.7564266 | down | histonedemethylaseUTY-like |
| 16858714 | 0.02397374 | 1.7559935 | down | ribonucleaseH2,subunitA |
| 16704436 | 0.049718514 | 1.7557973 | down | ArfGAPwithGTPasedomain,ankyrinrepeatandPHdomain9\|ArfGAPwithGTPasedomain,ankyrinrepeatandPHdomain10 |
| 16742671 | 0.027692072 | 1.7552358 | down | asparaginyl-tRNAsynthetase2,mitochondrial(putative) |
| 16986203 | 0.002496364 | 1.7552105 | down | hexosaminidaseB(betapolypeptide) |
| 16996946 | 0.03167617 | 1.7550939 | down | glucuronidase,betapseudogene3\|glucuronidase,betapseudogene9 |
| 16696270 | 0.030958658 | 1.7545524 | down | methyltransferaselike18 |
| 16759411 | 0.01108253 | 1.752565 | down | E1Abindingproteinp400 |
| 17061119 | 0.034527715 | 1.7524441 | down | RASp21proteinactivator4\|RASp21proteinactivator4B\|uroplakin3B-like |
| 16857457 | 0.009448472 | 1.751677 | down |  |
| 16706990 | 0.016481392 | 1.7515603 | down | multipleinositol-polyphosphatephosphatase1 |
| 16972396 | 0.014036734 | 1.7507844 | down | UDP-N-acetyl-alpha-D-galactosamine:polypeptideN-acetylgalactosaminyltransferase7(GalNAc-T7) |
| 17115692 | 0.006096767 | 1.75064 | down | membraneprotein,palmitoylated1,55kDa |
| 16904667 | 0.007739998 | 1.7489872 | down | sodiumchannel,voltage-gated,typeIX,alphasubunit |
| 16845817 | 0.01977733 | 1.7489138 | down | dephospho-CoAkinasedomaincontaining |
| 17087343 | 0.019280914 | 1.7485538 | down | nuclearcapbindingproteinsubunit1,80kDa |
| 16759592 | 0.006266638 | 1.7481043 | down | peroxisomalmembraneprotein2,22kDa |
| 17095882 | 0.003408392 | 1.747835 | down | osteomodulin |
| 16667303 | 0.001824201 | 1.7474105 | down | ATP-bindingcassette,sub-familyD(ALD),member3 |
| 16832429 | 0.03364918 | 1.7471188 | down | transmembraneprotein97 |
| 16698801 | 0.005445426 | 1.7468624 | down |  |
| 17098896 | 0.00703693 | 1.7455554 | down | highmobilitygroupAT-hook1pseudogene4 |
| 16930093 | 0.008266663 | 1.7446655 | down | translocaseofoutermitochondrialmembrane22homolog(yeast) |
| 17085082 | 0.003934089 | 1.7438507 | down | aldehydedehydrogenase1family,memberB1 |
| 16974534 | 0.020120636 | 1.7437123 | down | prominin1 |
| 16801557 | 0.014897425 | 1.7427623 | down | cyclinB2 |
| 16783983 | 0.046217564 | 1.7424513 | down | ATPsynthase,H+transporting,mitochondrialFocomplex,subunits(factorB) |
| 16693515 | 0.003468714 | 1.7423521 | down | interleukinenhancerbindingfactor2,45kDa |
| 17050154 | 0.04043499 | 1.7422739 | down | proteinkinase,cAMP-dependent,regulatory,typeII,beta |
| 16661910 | 0.006879835 | 1.7419355 | down |  |
| 16996201 | 0.022018539 | 1.7405692 | down | DEAH(Asp-Glu-Ala-His)boxpolypeptide29 |
| 17112729 | 0.025536623 | 1.7389221 | down | translocaseofinnermitochondrialmembrane8homologA(yeast) |
| 16820398 | 0.001837742 | 1.7380254 | down | solutecarrierfamily7(aminoacidtransporterlightchain,y+Lsystem),member6 |
| 16857972 | 0.03301282 | 1.736905 | down | zincfingerprotein559\|ZNF559-ZNF177readthrough\|zincfingerprotein177 |
| 16694529 | 0.023539511 | 1.7357658 | down | chaperonincontainingTCP1,subunit3(gamma) |
| 16795368 | 0.007600075 | 1.7357458 | down | stonin2 |
| 16832852 | 3.54E-04 | 1.7354312 | down | ATPasefamily,AAAdomaincontaining5 |
| 17095870 | 0.004358457 | 1.7349076 | down | osteoglycin |
| 16854702 | 2.16E-04 | 1.7342744 | down | INO80complexsubunitC |
| 16839261 | 0.009480426 | 1.7342502 | down | glyoxalasedomaincontaining4 |
| 17115801 | 0.03005147 | 1.7341379 | down | coagulationfactorVIII-associated3\|coagulationfactorVIII-associated2\|coagulationfactorVIII-associated1 |
| 16673598 | 0.027388372 | 1.7329333 | down |  |
| 16956621 | 0.025900876 | 1.7328588 | down | MYCinducednuclearantigen |
| 16985329 | 0.0208021 | 1.7321951 | down | neurolysin(metallopeptidaseM3family) |
| 17108582 | 0.029765563 | 1.7321807 | down | coagulationfactorVIII-associated3\|coagulationfactorVIII-associated2\|coagulationfactorVIII-associated1 |
| 16762597 | 0.02887432 | 1.7320552 | down | transmembrane7superfamilymember3 |
| 16925239 | 0.028908577 | 1.7316235 | down | downstreamneighborofSON |
| 16950448 | 0.002256129 | 1.7315896 | down |  |
| 17047397 | 0.014700729 | 1.7305135 | down | rhomboiddomaincontaining2 |
| 16752643 | 0.022878217 | 1.7287898 | down |  |
| 16834302 | 0.036968715 | 1.7287642 | down | ATPase,H+transporting,lysosomalV0subunita1 |
| 16907912 | 0.00467284 | 1.727837 | down | IKAROSfamilyzincfinger2(Helios) |
| 16794888 | 0.003146736 | 1.7266314 | down | acylphosphatase1,erythrocyte(common)type |
| 16826414 | 0.003172154 | 1.7265359 | down | bromodomaincontaining7 |
| 16765281 | 0.036681425 | 1.7263361 | down |  |
| 16845172 | 0.006136976 | 1.7256941 | down | PSMC3interactingprotein |
| 17104983 | 0.034461763 | 1.7253877 | down | chromosomeXopenreadingframe26 |
| 17000858 | 0.006760196 | 1.7253267 | down | diaphanoushomolog1(Drosophila) |
| 17106822 | 0.035077125 | 1.7251635 | down | oculocerebrorenalsyndromeofLowe |
| 16785509 | 0.032112904 | 1.7250268 | down |  |
| 16800871 | 0.021578247 | 1.7242135 | down | EP300interactinginhibitorofdifferentiation1 |
| 16915448 | 0.045489136 | 1.7240367 | down | familywithsequencesimilarity217,memberB |
| 16858344 | 4.14E-04 | 1.7238923 | down | SWI/SNFrelated,matrixassociated,actindependentregulatorofchromatin,subfamilya,member4 |
| 17025070 | 0.014851065 | 1.7226856 | down | transcriptionfactorB1,mitochondrial |
| 16862333 | 0.029219378 | 1.7223997 | down | smallnuclearribonucleoproteinpolypeptideA |
| 16772513 | 0.027578536 | 1.7193182 | down |  |
| 16828905 | 0.003313308 | 1.7191904 | down | ERmembraneproteincomplexsubunit8 |
| 16954576 | 6.58E-04 | 1.7188035 | down | Vpr(HIV-1)bindingprotein |
| 16930418 | 0.032812547 | 1.7187245 | down | adenylosuccinatelyase |
| 16854202 | 0.047545962 | 1.7181807 | down | abhydrolasedomaincontaining3 |
| 16911804 | 0.033402797 | 1.7177334 | down | solutecarrierfamily24(sodium/potassium/calciumexchanger),member3 |
| 16845567 | 0.021270815 | 1.7174639 | down | chromosome17openreadingframe65 |
| 16890574 | 4.15E-04 | 1.7174155 | down | X-rayrepaircomplementingdefectiverepairinChinesehamstercells5(double-strand-breakrejoining) |
| 17028547 | 0.019114679 | 1.7173786 | down |  |
| 16935069 | 0.039311405 | 1.7167492 | down |  |
| 16687829 | 0.031053443 | 1.7167125 | down | OMA1zincmetallopeptidasehomolog(S.cerevisiae)\|disabledhomolog1(Drosophila) |
| 16784582 | 0.017990649 | 1.7150711 | down | adaptor-relatedproteincomplex5,mu1subunit |
| 16837689 | 3.19E-04 | 1.7143533 | down | immaturecoloncarcinomatranscript1 |
| 16693259 | 0.01201645 | 1.7143528 | down | thioesterasesuperfamilymember4 |
| 16897056 | 0.004593033 | 1.7142234 | down | leucine-richpentatricopeptiderepeatcontaining |
| 16943868 | 0.008564775 | 1.7134204 | down | abhydrolasedomaincontaining10 |
| 16978568 | 0.018242974 | 1.713368 | down | centromereproteinE,312kDa |
| 16889026 | 0.011061929 | 1.7133241 | down | solutecarrierfamily39(zinctransporter),member10 |
| 16871655 | 0.03802945 | 1.7126385 | down | zincfingerprotein14homolog(mouse) |
| 16996545 | 0.013316333 | 1.7123079 | down | DEPdomaincontaining1B |
| 16854046 | 0.020461408 | 1.7118524 | down | centrosomalprotein76kDa |
| 17019208 | 0.001350522 | 1.7116003 | down | mitochondrialribosomalproteinS10 |
| 16847238 | 0.029442668 | 1.7113769 | down | spindleandkinetochoreassociatedcomplexsubunit2 |
| 16710271 | 0.018710684 | 1.7113605 | down | buddinguninhibitedbybenzimidazoles3homolog(yeast) |
| 16780793 | 7.61E-04 | 1.7111949 | down | testisexpressed30 |
| 16769443 | 0.007493935 | 1.710818 | down | nucleartranscriptionfactorY,beta |
| 16781536 | 0.001278812 | 1.7106732 | down | purinenucleosidephosphorylase |
| 16670081 | 0.012070662 | 1.710591 | down | chromodomainhelicaseDNAbindingprotein1-like |
| 16852331 | 0.001240293 | 1.7104534 | down | malicenzyme2,NAD(+)-dependent,mitochondrial |
| 16958236 | 0.037658095 | 1.7102519 | down | proteintyrosinephosphatase-like(prolineinsteadofcatalyticarginine),memberb |
| 16995339 | 0.036828097 | 1.7101089 | down | chromosome5openreadingframe42 |
| 16773874 | 0.026824472 | 1.7100159 | down | PDS5,regulatorofcohesionmaintenance,homologB(S.cerevisiae) |
| 16904773 | 0.008685566 | 1.7099457 | down |  |
| 16662123 | 0.048978955 | 1.7093258 | down | zincfingerandBTBdomaincontaining8A |
| 16841483 | 0.008631296 | 1.7092535 | down | elaChomolog2(E.coli) |
| 16696295 | 0.025710406 | 1.7090967 | down | kinesin-associatedprotein3 |
| 16953279 | 0.001405701 | 1.7084492 | down | celldivisioncycle25homologA(S.pombe) |
| 16885199 | 0.015512414 | 1.7081578 | down | translin |
| 16738429 | 0.003889974 | 1.7074783 | down | structurespecificrecognitionprotein1 |
| 16972750 | 0.032832395 | 1.7074515 | down | CDKN2Ainteractingprotein |
| 16959628 | 0.0352594 | 1.7068436 | down | debranchingenzymehomolog1(S.cerevisiae) |
| 16889932 | 0.04100991 | 1.7067968 | down |  |
| 16932914 | 0.020354893 | 1.7066802 | down | zincfingerprotein280B |
| 16821296 | 0.048163906 | 1.7063649 | down | c-Mafinducingprotein |
| 16959531 | 0.021014689 | 1.7061348 | down | stromalantigen1 |
| 16826985 | 6.15E-05 | 1.7061306 | down | glutamic-oxaloacetictransaminase2,mitochondrial(aspartateaminotransferase2) |
| 16705074 | 0.03072149 | 1.705878 | down | transcriptionfactorA,mitochondrial |
| 16937035 | 0.044488847 | 1.7058678 | down | ADP-ribosylationfactor-like8B |
| 16968378 | 0.008906977 | 1.7057763 | down | enolase-phosphatase1 |
| 16658184 | 0.001355937 | 1.7044259 | down | tumorproteinp63regulated1-like |
| 16790267 | 0.016969427 | 1.7041848 | down | suppressorofTy16homolog(S.cerevisiae) |
| 16968051 | 0.005587779 | 1.7039928 | down | septin11 |
| 16781516 | 0.004959902 | 1.7026163 | down | APEXnuclease(multifunctionalDNArepairenzyme)1 |
| 16885978 | 3.90E-04 | 1.7024337 | down | R3Hdomaincontaining1 |
| 17079448 | 0.009956188 | 1.7019336 | down | NIPA-likedomaincontaining2 |
| 16660527 | 0.024847485 | 1.701032 | down | zincfingerandBTBdomaincontaining40 |
| 16962921 | 0.015249486 | 1.700459 | down | ATPasetype13A3\|uncharacterizedLOC100507033 |
| 16768341 | 0.043100715 | 1.6992521 | down | ATPase,Ca++transporting,plasmamembrane1 |
| 17024633 | 0.018909588 | 1.6986974 | down | nucleoporin43kDa |
| 16918445 | 8.04E-04 | 1.6985638 | down | E2Ftranscriptionfactor1 |
| 16925194 | 3.26E-04 | 1.6975691 | down | phosphoribosylglycinamideformyltransferase,phosphoribosylglycinamidesynthetase,phosphoribosylaminoimidazolesynthetase |
| 17096423 | 0.021403827 | 1.6974646 | down | hemogen |
| 17092688 | 0.030009568 | 1.6952292 | down | HAUSaugmin-likecomplex,subunit6 |
| 16689113 | 0.020654164 | 1.6952262 | down | collagen,typeXXIV,alpha1 |
| 17086634 | 0.01313192 | 1.6947527 | down | CDC28proteinkinaseregulatorysubunit2 |
| 16983672 | 0.01742066 | 1.6939561 | down |  |
| 16950932 | 0.001739529 | 1.6925265 | down | nucleoporin210kDa |
| 16948236 | 0.023764512 | 1.6923016 | down | zincfingerprotein639 |
| 16743342 | 0.017560149 | 1.6922605 | down | TATAboxbindingprotein(TBP)-associatedfactor,RNApolymeraseI,D,41kDa\|microRNA1304\|smallnucleolarRNA,C/Dbox5\|smallnucleolarRNA,H/ACAbox32\|smallnucleolarRNA,H/ACAbox40\|smallnucleolarRNA,H/ACAbox18\|smallnucleolarRNA,H/ACAbox8 |
| 16678155 | 0.011259711 | 1.6920316 | down |  |
| 16990381 | 0.002826487 | 1.6917374 | down |  |
| 17051626 | 0.011477415 | 1.690586 | down | mesodermspecifictranscripthomolog(mouse) |
| 17074571 | 0.0233054 | 1.6904399 | down | PIN2/TERF1interacting,telomeraseinhibitor1\|SRY(sexdeterminingregionY)-box7 |
| 17069989 | 0.003845786 | 1.6903164 | down | telomericrepeatbindingfactor(NIMA-interacting)1 |
| 16951601 | 0.009867426 | 1.689198 | down | topoisomerase(DNA)IIbeta180kDa |
| 17099769 | 0.001292286 | 1.6887525 | down | calmodulinregulatedspectrin-associatedprotein1 |
| 16877019 | 0.003856503 | 1.6883569 | down | ribonucleotidereductaseM2 |
| 16666268 | 0.007588427 | 1.6881667 | down | Rabgeranylgeranyltransferase,betasubunit\|smallnucleolarRNA,C/Dbox45C\|smallnucleolarRNA,C/Dbox45A\|smallnucleolarRNA,C/Dbox45B |
| 16927198 | 0.011012426 | 1.6881425 | down | RANbindingprotein1 |
| 16716846 | 0.01006923 | 1.6869352 | down | aldehydedehydrogenase18family,memberA1 |
| 16718553 | 0.029808726 | 1.6864171 | down | DNAcross-linkrepair1A |
| 16822290 | 0.009738017 | 1.6855792 | down | polymerase(RNA)III(DNAdirected)polypeptideK,12.3kDa |
| 16846993 | 0.015809119 | 1.6853245 | down | serine/arginine-richsplicingfactor1 |
| 16717943 | 0.003325084 | 1.6849661 | down | ARP1actin-relatedprotein1homologA,centractinalpha(yeast) |
| 16936306 | 0.003518202 | 1.684527 | down | megalencephalicleukoencephalopathywithsubcorticalcysts1 |
| 16855820 | 0.001065152 | 1.6838198 | down | rotatin |
| 16855355 | 0.034251735 | 1.6838031 | down |  |
| 16824352 | 5.46E-04 | 1.6834269 | down | xylosyltransferaseI |
| 16803562 | 8.73E-04 | 1.6830602 | down | cholinergicreceptor,nicotinic,alpha5(neuronal) |
| 16794767 | 0.001043884 | 1.6824875 | down |  |
| 17015987 | 0.008127008 | 1.6823299 | down |  |
| 16672097 | 0.013886835 | 1.6815134 | down | apolipoproteinA-Ibindingprotein |
| 16803317 | 0.025701415 | 1.6812689 | down | reticulocalbin2,EF-handcalciumbindingdomain |
| 16846016 | 0.007185595 | 1.6803937 | down | ADP-ribosylationfactor-like17B\|ADP-ribosylationfactor-likeprotein17-like\|ADP-ribosylationfactor-like17A |
| 16900605 | 8.70E-04 | 1.6802855 | down |  |
| 17043437 | 0.035069395 | 1.6799132 | down | chromosome7openreadingframe26 |
| 16877762 | 0.008140073 | 1.6798766 | down | centromereproteinO |
| 16882258 | 0.002367807 | 1.6791427 | down | methionineadenosyltransferaseII,alpha |
| 16853828 | 0.007385714 | 1.6788149 | down | proteinphosphatase4,regulatorysubunit1 |
| 16982011 | 0.025827719 | 1.6785827 | down | caspase3,apoptosis-relatedcysteinepeptidase |
| 16843728 | 0.005354206 | 1.6784279 | down | acetyl-CoAcarboxylasealpha |
| 16987914 | 0.005233742 | 1.6783442 | down | WDrepeatdomain36 |
| 16900734 | 0.013652079 | 1.6781679 | down |  |
| 16977461 | 0.020520624 | 1.6781143 | down | SEC31homologA(S.cerevisiae)\|THAP9antisenseRNA1(non-proteincoding) |
| 16771894 | 0.025873031 | 1.6774157 | down | M-phasephosphoprotein9 |
| 16664559 | 0.004648896 | 1.6771544 | down |  |
| 16713628 | 0.009922555 | 1.6769944 | down | zincfinger,AN1-typedomain4 |
| 16705778 | 0.011552156 | 1.6769327 | down | sphingosine-1-phosphatelyase1 |
| 16835589 | 0.005112682 | 1.6753937 | down |  |
| 16908415 | 0.00213032 | 1.6750551 | down | zincfingerprotein142 |
| 16777274 | 0.009212784 | 1.6743679 | down | RNA,U4smallnuclear9,pseudogene |
| 16860499 | 0.0026694 | 1.6742942 | down | zincfingerprotein507 |
| 16973797 | 0.031036774 | 1.6742569 | down |  |
| 16881136 | 0.007525567 | 1.6740669 | down |  |
| 16708668 | 0.002148425 | 1.6732007 | down |  |
| 16845349 | 0.00392237 | 1.6729989 | down | breastcancer1,earlyonset\|uncharacterizedLOC100505899 |
| 16975659 | 0.048991796 | 1.6716965 | down | COMMdomaincontaining8 |
| 16755115 | 3.81E-04 | 1.6713873 | down | mitochondrialribosomalproteinL42 |
| 17062255 | 0.02982473 | 1.671182 | down | familywithsequencesimilarity3,memberC |
| 16953735 | 0.0286364 | 1.6710421 | down | proteinkinase,cAMP-dependent,regulatory,typeII,alpha |
| 16824429 | 7.03E-04 | 1.6706308 | down | NODALmodulator2\|NODALmodulator1\|NODALmodulator3 |
| 16878358 | 0.002621953 | 1.6706047 | down | GPN-loopGTPase1\|zincfingerprotein512 |
| 16697695 | 3.19E-04 | 1.6702254 | down | kinesinfamilymember14 |
| 16763853 | 0.007580763 | 1.6694301 | down | SUMO1/sentrinspecificpeptidase1 |
| 16746808 | 0.0029009 | 1.6691737 | down | FK506bindingprotein4,59kDa |
| 16815246 | 0.006303683 | 1.6687442 | down | zymogengranuleprotein16homologB(rat) |
| 16888047 | 0.003586132 | 1.6687272 | down | alkylglyceronephosphatesynthase |
| 16894424 | 0.003668167 | 1.6685157 | down | nucleolarprotein10 |
| 16663064 | 0.031897448 | 1.6679368 | down | zincfingerprotein642 |
| 17068956 | 3.71E-04 | 1.6677055 | down | mitochondrialribosomalproteinL15 |
| 17022216 | 0.018478943 | 1.6674463 | down | reticulon4interactingprotein1 |
| 16906509 | 0.037998114 | 1.6671319 | down | transmembraneprotein194B |
| 16768579 | 0.001334872 | 1.6671176 | down | coiled-coildomaincontaining41 |
| 16658889 | 0.004565691 | 1.6670612 | down | apoptosis-inducing,TAF9-likedomain1\|APITD1-CORTreadthrough\|cortistatin |
| 17045198 | 0.001947156 | 1.666235 | down | anillin,actinbindingprotein |
| 16991180 | 0.011265044 | 1.6654866 | down | chromosome5openreadingframe62 |
| 17066961 | 0.009883698 | 1.6645561 | down | ADAM-like,decysin1 |
| 17079968 | 0.014961187 | 1.66451 | down |  |
| 17072313 | 0.047477294 | 1.6637667 | down | WDrepeatdomain67 |
| 17043355 | 0.005809553 | 1.6633493 | down | aminoacyltRNAsynthetasecomplex-interactingmultifunctionalprotein2 |
| 16811816 | 0.04715401 | 1.6618283 | down | proteintyrosinephosphatase,non-receptortype9 |
| 16744415 | 0.031353027 | 1.6617742 | down | interleukin18(interferon-gamma-inducingfactor) |
| 16819952 | 0.002253718 | 1.6601753 | down | core-bindingfactor,betasubunit |
| 17006378 | 0.003520682 | 1.6597914 | down | tubulin,betaclassI\|tubulin,betapseudogene1 |
| 16906000 | 0.001655368 | 1.6596507 | down | SEC14andspectrindomains1 |
| 17075604 | 0.027524548 | 1.6582205 | down | potassiumchanneltetramerisationdomaincontaining9\|potassiumchanneltetramerisationdomaincontaining9pseudogene2 |
| 16833441 | 0.040319543 | 1.6581814 | down | zincfinger,HIT-typecontaining3 |
| 17018382 | 0.011928231 | 1.6581511 | down |  |
| 16768205 | 0.044940047 | 1.6576996 | down | centrosomalprotein290kDa |
| 16846181 | 0.012221836 | 1.6576607 | down | chromoboxhomolog1 |
| 16765513 | 0.01830268 | 1.6567954 | down | chromoboxhomolog5 |
| 17018685 | 0.00267182 | 1.6561852 | down | peptidylprolylisomerase(cyclophilin)-like1 |
| 16895179 | 0.01319812 | 1.655887 | down | tumorproteinp53inducibleprotein3 |
| 16836156 | 0.015386076 | 1.6557542 | down |  |
| 16785631 | 0.01018336 | 1.6556212 | down | eukaryotictranslationinitiationfactor2,subunit1alpha,35kDa |
| 16962264 | 0.02091706 | 1.6546179 | down | melanomaantigenfamilyF,1 |
| 17097281 | 0.004723659 | 1.6546073 | down | sushidomaincontaining1 |
| 16995281 | 0.00268683 | 1.6540956 | down | NADkinasedomaincontaining1 |
| 16820992 | 0.020322539 | 1.653876 | down |  |
| 16702656 | 0.001679157 | 1.6537958 | down | heatshock70kDaprotein14 |
| 16843241 | 0.018600173 | 1.653706 | down | myosinID |
| 16688308 | 0.014592771 | 1.6526712 | down | SERPINE1mRNAbindingprotein1 |
| 16825794 | 0.02776012 | 1.6525154 | down | dCTPpyrophosphatase1 |
| 16884520 | 0.007841361 | 1.6522919 | down |  |
| 16828734 | 0.007739215 | 1.6522698 | down | membrane-boundtranscriptionfactorpeptidase,site1 |
| 16994890 | 0.030777143 | 1.6520905 | down | drosha,ribonucleasetypeIII |
| 17023150 | 0.01796697 | 1.6518223 | down | mannosidase,alpha,class1A,member1 |
| 17095887 | 0.018857816 | 1.6514287 | down | asporin |
| 16781591 | 0.020408854 | 1.6504985 | down | ribonuclease,RNaseAfamily,k6 |
| 16762798 | 0.017115263 | 1.6498405 | down | importin8 |
| 16799259 | 0.021355057 | 1.649255 | down |  |
| 16731189 | 0.04199693 | 1.6483872 | down | chromosome11openreadingframe57 |
| 16846675 | 0.010146857 | 1.6480265 | down | leucinerichrepeatcontaining59 |
| 17062639 | 0.04619655 | 1.6474857 | down | RNAbindingmotifprotein28 |
| 16943435 | 0.036535576 | 1.6472337 | down | centrosomalprotein97kDa |
| 17092302 | 0.008028723 | 1.6467319 | down |  |
| 17097869 | 0.002447416 | 1.6464595 | down | multipleEGF-like-domains9 |
| 16939910 | 0.021979192 | 1.6461248 | down | zincfingerprotein660\|zincfingerprotein197 |
| 17032627 | 0.033104293 | 1.6460158 | down | vacuolarproteinsorting52homolog(S.cerevisiae) |
| 16820193 | 0.001021079 | 1.6457812 | down | CCCTC-bindingfactor(zincfingerprotein) |
| 17076624 | 0.004825132 | 1.6455172 | down |  |
| 17018733 | 0.024173494 | 1.6454235 | down | coiled-coildomaincontaining167 |
| 16997046 | 0.04973718 | 1.6453027 | down | glucuronidase,betapseudogene9\|glucuronidase,betapseudogene3\|glucuronidase,betapseudogene2 |
| 16841852 | 0.045659296 | 1.6438202 | down | phospholipaseDfamily,member6 |
| 16663075 | 0.028117478 | 1.6438104 | down | defectsinmorphology1homolog(S.cerevisiae) |
| 16701324 | 0.004572189 | 1.6436594 | down | heterogeneousnuclearribonucleoproteinU(scaffoldattachmentfactorA) |
| 16853377 | 0.016419183 | 1.6423839 | down | THOcomplex1 |
| 17052252 | 0.009391793 | 1.642321 | down | thromboxaneAsynthase1(platelet) |
| 16823083 | 0.005951251 | 1.6420977 | down | phosphoglycolatephosphatase |
| 17055630 | 0.029497737 | 1.6419703 | down | TWISTneighbor |
| 16888087 | 0.012341542 | 1.6414403 | down | RNAbindingmotifprotein45 |
| 16911943 | 0.001446331 | 1.6410682 | down | 5'-3'exoribonuclease2 |
| 16715409 | 0.004615231 | 1.6410229 | down | DnaJ(Hsp40)homolog,subfamilyC,member9\|uncharacterizedLOC100653136\|uncharacterizedLOC100652993\|mitochondrialribosomalproteinS16 |
| 17089982 | 0.010534645 | 1.6404717 | down | dolichylpyrophosphatephosphatase1 |
| 17012165 | 0.003162389 | 1.63992 | down | heatshocktranscriptionfactor2 |
| 16791669 | 0.008129891 | 1.6398287 | down | HECTdomaincontainingE3ubiquitinproteinligase1 |
| 16719515 | 0.007739896 | 1.639299 | down | antigenidentifiedbymonoclonalantibodyKi-67 |
| 17006230 | 0.044345256 | 1.6390337 | down |  |
| 16778325 | 4.97E-04 | 1.6383104 | down | prolineandserinerich1 |
| 16784033 | 0.003789811 | 1.6382821 | down |  |
| 16707551 | 0.018370073 | 1.6376954 | down | centrosomalprotein55kDa |
| 17012804 | 0.02831655 | 1.637633 | down | v-mybmyeloblastosisviraloncogenehomolog(avian) |
| 16945101 | 0.002237938 | 1.6366799 | down | minichromosomemaintenancecomplexcomponent2 |
| 16842673 | 9.78E-04 | 1.636398 | down | spermassociatedantigen5\|uncharacterizedserine/threonine-proteinkinaseSgK494 |
| 16780664 | 0.0191035 | 1.6362607 | down | transmembraneandtetratricopeptiderepeatcontaining4 |
| 16927025 | 0.026552476 | 1.6358675 | down |  |
| 16683271 | 0.003235392 | 1.6358092 | down | heterogeneousnuclearribonucleoproteinR |
| 16954856 | 0.001257192 | 1.6353472 | down | polybromo1 |
| 17090893 | 0.008641079 | 1.635217 | down | WDrepeatdomain5 |
| 16862066 | 6.06E-04 | 1.6350873 | down | translocaseofinnermitochondrialmembrane50homolog(S.cerevisiae) |
| 16808314 | 0.045753382 | 1.6350689 | down | diphosphoinositolpentakisphosphatekinase1 |
| 16884703 | 0.003936299 | 1.6350302 | down | COBWdomaincontaining1\|COBWdomaincontaining2\|COBWdomaincontaining3\|COBWdomaincontaining5\|COBWdomaincontaining6\|COBWdomaincontaining7\|uncharacterizedLOC100653334 |
| 17047338 | 0.003870957 | 1.6338795 | down |  |
| 16767794 | 0.03902688 | 1.6333975 | down | oxysterolbindingprotein-like8 |
| 17019728 | 0.037711885 | 1.6328521 | down | phospholipaseA2,groupVII(platelet-activatingfactoracetylhydrolase,plasma) |
| 16699320 | 0.017491613 | 1.632329 | down | Gpatchdomaincontaining2 |
| 16678838 | 0.010024183 | 1.6321237 | down | nucleoside-triphosphatase,cancer-related |
| 16963358 | 0.026491495 | 1.631845 | down | ringfingerprotein168,E3ubiquitinproteinligase |
| 16888317 | 0.014780524 | 1.6317824 | down | spermspecificantigen2 |
| 17011671 | 0.020587593 | 1.6316288 | down | adenosylmethioninedecarboxylase1 |
| 16808751 | 0.033466082 | 1.6315199 | down | myelinexpressionfactor2 |
| 17059932 | 0.0346005 | 1.6304162 | down | paraoxonase2 |
| 16825468 | 0.006924084 | 1.6302886 | down | bolAhomolog2(E.coli)\|bolAhomolog2B(E.coli)\|smg-1homolog,phosphatidylinositol3-kinase-relatedkinase(C.elegans)pseudogene |
| 16763021 | 0.036804914 | 1.6301411 | down | tyrosyl-tRNAsynthetase2,mitochondrial |
| 17027947 | 0.013790042 | 1.630078 | down |  |
| 16825683 | 0.006901358 | 1.6300623 | down | bolAhomolog2(E.coli)\|bolAhomolog2B(E.coli)\|smg-1homolog,phosphatidylinositol3-kinase-relatedkinase(C.elegans)pseudogene |
| 16860531 | 0.004847038 | 1.6295986 | down | programmedcelldeath5 |
| 17015434 | 0.04459345 | 1.6287527 | down | mutedhomolog(mouse)\|EEF1E1-MUTEDreadthrough\|MUTED-TXNDC5readthrough(non-proteincoding)\|thioredoxindomaincontaining5(endoplasmicreticulum)\|eukaryotictranslationelongationfactor1epsilon1 |
| 16839331 | 0.008422036 | 1.6277179 | down | tyrosine3-monooxygenase/tryptophan5-monooxygenaseactivationprotein,epsilonpolypeptide |
| 17003401 | 0.017644912 | 1.6275507 | down | chromosome5openreadingframe25pseudogene |
| 17023095 | 0.002632789 | 1.6271868 | down | minichromosomemaintenancecomplexcomponent9 |
| 17109775 | 0.011405359 | 1.626686 | down |  |
| 16703452 | 0.037652213 | 1.626592 | down | prenyl(decaprenyl)diphosphatesynthase,subunit1 |
| 16808306 | 0.001626733 | 1.6257695 | down | diphosphoinositolpentakisphosphatekinase1 |
| 17093122 | 0.002784205 | 1.624899 | down | NADHdehydrogenase(ubiquinone)1betasubcomplex,6,17kDa |
| 16783925 | 0.004079416 | 1.6247447 | down |  |
| 16766001 | 0.029717188 | 1.6246234 | down | citratesynthase |
| 17024980 | 0.005542042 | 1.6245413 | down | F-boxprotein5 |
| 16758242 | 0.011821691 | 1.6243963 | down | B-cellCLL/lymphoma7A |
| 17029942 | 0.033928923 | 1.6236016 | down | vacuolarproteinsorting52homolog(S.cerevisiae) |
| 16756334 | 0.002656991 | 1.6234865 | down | polymerase(RNA)III(DNAdirected)polypeptideB |
| 16833263 | 0.001628664 | 1.6233811 | down | ligaseIII,DNA,ATP-dependent |
| 16791781 | 0.019116364 | 1.6232806 | down | chromosome14openreadingframe126 |
| 16745946 | 0.01854301 | 1.6230385 | down |  |
| 16834441 | 0.001165926 | 1.6229488 | down | vacuolarproteinsorting25homolog(S.cerevisiae) |
| 16856257 | 0.013384035 | 1.6225928 | down | polypyrimidinetractbindingprotein1 |
| 16954925 | 0.019425265 | 1.6224868 | down | NIMA(neverinmitosisgenea)-relatedkinase4 |
| 16761843 | 4.59E-04 | 1.6220938 | down | RhoGDPdissociationinhibitor(GDI)beta |
| 16991859 | 0.007494825 | 1.620991 | down | hyaluronan-mediatedmotilityreceptor(RHAMM) |
| 16804557 | 0.012721136 | 1.6206138 | down |  |
| 17007632 | 0.02924929 | 1.6199948 | down |  |
| 17018130 | 0.035086975 | 1.6195318 | down | vacuolarproteinsorting52homolog(S.cerevisiae) |
| 16695888 | 0.013025657 | 1.6195209 | down | aldehydedehydrogenase9family,memberA1 |
| 16842722 | 0.004719922 | 1.619146 | down |  |
| 17042619 | 0.034877025 | 1.6187557 | down | vacuolarproteinsorting52homolog(S.cerevisiae) |
| 16840759 | 0.00748123 | 1.6187018 | down | LSMdomaincontaining1 |
| 16786432 | 0.025401864 | 1.6185813 | down | lin-52homolog(C.elegans) |
| 17037427 | 0.034625027 | 1.6184292 | down | vacuolarproteinsorting52homolog(S.cerevisiae) |
| 16660098 | 0.002607827 | 1.6183009 | down |  |
| 16866946 | 0.010901847 | 1.6182663 | down | translocaseofinnermitochondrialmembrane13homolog(yeast) |
| 16870131 | 0.041881863 | 1.617741 | down | HAUSaugmin-likecomplex,subunit8 |
| 16677425 | 0.013460988 | 1.6175889 | down | centromereproteinF,350/400kDa(mitosin) |
| 16758697 | 0.048424505 | 1.6169455 | down | ATPase,H+transporting,lysosomalV0subunita2 |
| 16837029 | 0.02210272 | 1.6166487 | down | proteasome(prosome,macropain)26Ssubunit,ATPase,5 |
| 16709298 | 0.033836536 | 1.6159495 | down | vesicletransportthroughinteractionwitht-SNAREshomolog1A(yeast) |
| 16699066 | 0.001039967 | 1.615769 | down | transmembraneprotein206 |
| 16766260 | 0.014958294 | 1.615365 | down | ATPsynthase,H+transporting,mitochondrialF1complex,betapolypeptide |
| 16843680 | 1.05E-04 | 1.6153213 | down | myosinXIX |
| 17072225 | 0.017649833 | 1.6152883 | down | Mdm2,transformed3T3celldoubleminute2,p53bindingprotein(mouse)bindingprotein,104kDa |
| 16728994 | 0.002615386 | 1.6150427 | down | polymerase(DNA-directed),delta3,accessorysubunit |
| 16825866 | 0.03361141 | 1.6149458 | down |  |
| 17071119 | 3.25E-04 | 1.6142895 | down | phosphatidylserinesynthase1 |
| 17061110 | 0.011896425 | 1.6140606 | down | RASp21proteinactivator4\|RASp21proteinactivator4C,pseudogene\|RASp21proteinactivator4B\|uroplakin3B-like |
| 16937152 | 0.03125983 | 1.6135155 | down | longintergenicnon-proteincodingRNA312 |
| 16845589 | 4.04E-05 | 1.6131445 | down | upstreambindingtranscriptionfactor,RNApolymeraseI |
| 17056587 | 0.001983998 | 1.6124183 | down | dpy-19-like1(C.elegans) |
| 16690843 | 0.008341331 | 1.6118834 | down | WDrepeatdomain77 |
| 16851349 | 0.017808286 | 1.6115575 | down | microRNA320c-1 |
| 16725589 | 0.009402222 | 1.6109239 | down | transmembraneprotein138 |
| 16760465 | 0.005332965 | 1.6100543 | down | chromodomainhelicaseDNAbindingprotein4 |
| 16932300 | 0.0491941 | 1.6098226 | down | ubiquitinfusiondegradation1like(yeast) |
| 16763246 | 0.025686186 | 1.6097621 | down | glucosidexylosyltransferase1 |
| 17026012 | 0.01868483 | 1.6092378 | down | programmedcelldeath2 |
| 16708312 | 0.004037811 | 1.6091897 | down | familywithsequencesimilarity178,memberA |
| 16881514 | 0.004297823 | 1.6088718 | down | Alstromsyndrome1 |
| 16667206 | 0.045463745 | 1.6084241 | down | coiled-coildomaincontaining18 |
| 16730935 | 4.21E-04 | 1.6084121 | down | DEAD(Asp-Glu-Ala-Asp)boxpolypeptide10 |
| 16846714 | 4.45E-04 | 1.6074306 | down | ankyrinrepeatdomain40 |
| 17071625 | 0.009389265 | 1.6074206 | down | frizzledfamilyreceptor6 |
| 16815411 | 0.039094634 | 1.6074139 | down | uncharacterizedproteinFLJ39639\|zincfingerprotein263 |
| 16883107 | 0.046869732 | 1.6069477 | down | ankyrinrepeatdomain36\|ankyrinrepeatdomain36C |
| 17052685 | 8.04E-04 | 1.6068333 | down | Tcellreceptorbetaconstant2 |
| 16808304 | 0.03647978 | 1.6061404 | down | diphosphoinositolpentakisphosphatekinase1 |
| 17041494 | 0.002985563 | 1.6061234 | down | zincfingerprotein57homolog(mouse) |
| 16887635 | 0.003041319 | 1.6055502 | down | histoneacetyltransferase1 |
| 16891575 | 0.021964353 | 1.6053883 | down | acyl-CoAsynthetaselong-chainfamilymember3 |
| 17082604 | 0.005350976 | 1.605157 | down | cleavageandpolyadenylationspecificfactor1,160kDa\|microRNA1234\|microRNA939 |
| 16726224 | 4.62E-04 | 1.6045141 | down | stress-induced-phosphoprotein1 |
| 16671503 | 0.004697709 | 1.6043373 | down | CDC28proteinkinaseregulatorysubunit1B |
| 16779546 | 0.007798267 | 1.60432 | down | diaphanoushomolog3(Drosophila) |
| 16829369 | 0.008487633 | 1.6040078 | down | Fanconianemia,complementationgroupA |
| 16757710 | 0.009183016 | 1.603834 | down | phosphatidylethanolaminebindingprotein1 |
| 16792381 | 0.005066682 | 1.6038159 | down | MIS18bindingprotein1 |
| 16879174 | 0.046033144 | 1.603592 | down | glutaminyl-peptidecyclotransferase |
| 16912625 | 0.010914247 | 1.6033893 | down | microtubule-associatedprotein,RP/EBfamily,member1 |
| 16766683 | 0.022887142 | 1.6032078 | down | cyclin-dependentkinase4 |
| 17000066 | 0.019161463 | 1.6031989 | down | CDKN2AinteractingproteinN-terminallike |
| 17027559 | 0.006333182 | 1.6029699 | down | tubulin,betaclassI |
| 16934227 | 0.013077568 | 1.6022971 | down | chromosome22openreadingframe28 |
| 16690343 | 0.017854553 | 1.6022569 | down | vav3guaninenucleotideexchangefactor |
| 16833476 | 0.011591291 | 1.6014049 | down | dehydrogenase/reductase(SDRfamily)member11 |
| 16991374 | 0.047998648 | 1.6013339 | down | SAP30-like |
| 16704909 | 0.008395532 | 1.6012026 | down | ArfGAPwithGTPasedomain,ankyrinrepeatandPHdomain6 |
| 16707884 | 0.024585878 | 1.6003155 | down | zincfingerprotein518A |
| 16814504 | 0.016234623 | 1.5999203 | down | familywithsequencesimilarity173,memberA |
| 16670739 | 0.006947871 | 1.5998968 | down | myeloid/lymphoidormixed-lineageleukemia(trithoraxhomolog,Drosophila) |
| 16768936 | 0.041308988 | 1.5996982 | down | TMPOantisenseRNA1(non-proteincoding) |
| 16745525 | 4.38E-04 | 1.599095 | down | heatshock70kDaprotein8\|smallnucleolarRNA,C/Dbox14D\|smallnucleolarRNA,C/Dbox14C |
| 16933630 | 0.002735454 | 1.5987811 | down | THOcomplex5 |
| 17066018 | 0.016824046 | 1.5984786 | down | zincfinger,DHHC-typecontaining2 |
| 17077244 | 0.014436913 | 1.5982637 | down | lysophospholipaseI |
| 17022936 | 0.016197588 | 1.598168 | down |  |
| 16937793 | 0.019667635 | 1.5973355 | down |  |
| 17030404 | 0.005549016 | 1.5972817 | down | tubulin,betaclassI |
| 16843187 | 0.021983618 | 1.5967798 | down |  |
| 16737314 | 0.031649977 | 1.5960556 | down |  |
| 16957106 | 0.035446145 | 1.5955215 | down | intraflagellartransport57homolog(Chlamydomonas) |
| 17035229 | 0.005286913 | 1.5954973 | down | tubulin,betaclassI |
| 17100585 | 0.015926149 | 1.5946152 | down | zincfinger,MYND-typecontaining19 |
| 16772625 | 0.001164361 | 1.5937408 | down | polymerase(DNAdirected),epsilon,catalyticsubunit |
| 16662908 | 0.04280271 | 1.5935706 | down | peptidylprolylisomeraseE(cyclophilinE) |
| 17065047 | 0.001923399 | 1.5935049 | down |  |
| 17062878 | 0.002006137 | 1.593347 | down | centrosomalprotein41kDa |
| 17033109 | 0.006059338 | 1.5931183 | down | tubulin,betaclassI |
| 17040137 | 0.039593276 | 1.5930488 | down | vacuolarproteinsorting52homolog(S.cerevisiae) |
| 17111688 | 0.021970557 | 1.5928494 | down | LAS1-like(S.cerevisiae) |
| 17076273 | 0.0082729 | 1.5924504 | down | chromosomeXopenreadingframe56pseudogene |
| 16947173 | 0.005998633 | 1.591967 | down | membranemetallo-endopeptidase |
| 17117633 | 3.09E-04 | 1.5913315 | down |  |
| 16757209 | 0.011364177 | 1.5912999 | down | ADAMmetallopeptidasedomain1,pseudogene |
| 16993156 | 0.041816384 | 1.5912158 | down |  |
| 16817363 | 0.017546194 | 1.591092 | down | eukaryotictranslationinitiationfactor3,subunitC\|eukaryotictranslationinitiationfactor3,subunitC-like |
| 16738205 | 0.004817123 | 1.5908366 | down | nucleoporin160kDa |
| 16705715 | 0.00392465 | 1.5906177 | down | eukaryotictranslationinitiationfactor4Ebindingprotein2 |
| 17026211 | 0.006067289 | 1.5906142 | down | tubulin,betaclassI |
| 16815985 | 0.023410685 | 1.5904882 | down | MKL/myocardin-like2 |
| 17037888 | 0.006023462 | 1.5903044 | down | tubulin,betaclassI |
| 17040602 | 0.006126467 | 1.5901316 | down | tubulin,betaclassI |
| 16809403 | 0.001311447 | 1.5894747 | down | myosinVC |
| 16851801 | 0.022494044 | 1.589425 | down | ringfingerprotein125,E3ubiquitinproteinligase |
| 16829885 | 0.018501535 | 1.5888512 | down | proteasome(prosome,macropain)subunit,betatype,6 |
| 16778688 | 0.010718812 | 1.5881723 | down | nuclearfragileXmentalretardationproteininteractingprotein1 |
| 16715028 | 0.007207606 | 1.5880815 | down |  |
| 16918522 | 7.42E-04 | 1.5877373 | down | phosphatidylinositolglycananchorbiosynthesis,classU |
| 17047965 | 2.34E-04 | 1.587434 | down |  |
| 16911698 | 0.017180772 | 1.5869849 | down | CSRP2bindingprotein |
| 17004048 | 0.041318923 | 1.5868754 | down | zincfingerprotein62homolog(mouse) |
| 16935517 | 7.56E-04 | 1.5863721 | down | centromereproteinM |
| 16749759 | 0.033576004 | 1.5863613 | down | bicaudalDhomolog1(Drosophila) |
| 16997519 | 0.002766222 | 1.5863116 | down | arylsulfataseB |
| 16691752 | 0.020841481 | 1.5858349 | down |  |
| 16662737 | 0.012006138 | 1.5853575 | down | akirin1 |
| 16997860 | 0.017835762 | 1.5853075 | down | cyclinH |
| 17001324 | 0.015945626 | 1.5849361 | down | januskinaseandmicrotubuleinteractingprotein2 |
| 16849982 | 0.003375385 | 1.5844493 | down | Aly/REFexportfactor |
| 16836260 | 0.015848756 | 1.5841457 | down | hepaticleukemiafactor |
| 16929193 | 0.03446864 | 1.5835453 | down |  |
| 17044250 | 0.029686378 | 1.5832984 | down |  |
| 16817006 | 0.03323249 | 1.5831007 | down | dynactin5(p25) |
| 16695048 | 0.034348235 | 1.5827764 | down | spectrin,alpha,erythrocytic1(elliptocytosis2) |
| 16961379 | 0.040095605 | 1.581789 | down | leucinerichrepeatcontaining34 |
| 16668702 | 0.010197662 | 1.5814035 | down | chromosome1openreadingframe162 |
| 16881485 | 0.0340597 | 1.5812961 | down | chaperonincontainingTCP1,subunit7(eta) |
| 16847795 | 0.001889394 | 1.5809909 | down | testisexpressed2 |
| 16883201 | 0.02008696 | 1.5809759 | down |  |
| 17093031 | 0.01805801 | 1.5806128 | down | MOBkinaseactivator3B |
| 16721759 | 0.009385531 | 1.5806041 | down | TMEM9BantisenseRNA1(non-proteincoding) |
| 17044698 | 0.03573483 | 1.5800211 | down |  |
| 16944386 | 0.030805035 | 1.5799129 | down | translocaseofinnermitochondrialmembranedomaincontaining1 |
| 16941184 | 0.006131377 | 1.5797963 | down | RAD54-like2(S.cerevisiae) |
| 16924979 | 0.006256069 | 1.5797616 | down |  |
| 17004642 | 0.002032353 | 1.5797573 | down | smallnuclearribonucleoprotein48kDa(U11/U12) |
| 16682989 | 0.015630625 | 1.5796828 | down | endothelinconvertingenzyme1 |
| 16882277 | 0.002585917 | 1.57884 | down | vesicle-associatedmembraneprotein8(endobrevin) |
| 17093204 | 0.005363234 | 1.5787863 | down | BCL2-associatedathanogene |
| 16666851 | 0.00959536 | 1.5780616 | down | heparansulfate2-O-sulfotransferase1\|uncharacterizedLOC339524 |
| 16860168 | 0.008164141 | 1.5780531 | down | zincfingerprotein431 |
| 16670404 | 0.016856574 | 1.5778829 | down | vacuolarproteinsorting45homolog(S.cerevisiae) |
| 16796607 | 0.02021982 | 1.5776495 | down | SETdomaincontaining3 |
| 16996917 | 0.017444156 | 1.5774941 | down |  |
| 17095111 | 0.03006051 | 1.5773827 | down | guaninenucleotidebindingprotein(Gprotein),qpolypeptide |
| 16913456 | 0.046791635 | 1.5769188 | down | catenin,betalike1 |
| 16661589 | 0.023692004 | 1.5767212 | down | regulatorofchromosomecondensation1\|smallnucleolarRNAhostgene3(non-proteincoding)\|smallnucleolarRNA,H/ACAbox73A |
| 16940738 | 0.022170462 | 1.5762028 | down | N-acylaminoacyl-peptidehydrolase |
| 17051965 | 0.008311051 | 1.5761704 | down | nucleoporin205kDa |
| 16964749 | 6.35E-05 | 1.5758477 | down |  |
| 16807600 | 0.013640347 | 1.5754061 | down |  |
| 16726936 | 0.005177431 | 1.5752794 | down | familywithsequencesimilarity89,memberB |
| 17088589 | 0.007290161 | 1.5730231 | down | centriolin |
| 16743374 | 0.01962209 | 1.5727377 | down | MRE11meioticrecombination11homologA(S.cerevisiae) |
| 16810633 | 0.003803586 | 1.5726064 | down | mitochondrialmethionyl-tRNAformyltransferase |
| 17117613 | 0.04977143 | 1.5724148 | down | ankyrinrepeatdomain10\|ANKRD10intronictranscript1(non-proteincoding) |
| 16864708 | 0.002176373 | 1.5722673 | down |  |
| 16683676 | 0.003219064 | 1.5719488 | down |  |
| 16694716 | 0.009661217 | 1.5719273 | down | interferonstimulatedexonucleasegene20kDa-like2 |
| 17077399 | 0.018281076 | 1.5716274 | down | inositolmonophosphatasedomaincontaining1 |
| 16825306 | 0.01363418 | 1.5703912 | down | eukaryotictranslationinitiationfactor3,subunitC-like |
| 16919915 | 0.02460826 | 1.5702236 | down | zincfinger,MYND-typecontaining8 |
| 17113129 | 3.84E-05 | 1.5700893 | down | nucleoporin62kDaC-terminallike |
| 16689581 | 0.017837524 | 1.5699455 | down | glomulin,FKBPassociatedprotein |
| 16848739 | 0.00291451 | 1.5694631 | down | hematologicalandneurologicalexpressed1 |
| 16842070 | 0.018145552 | 1.5682533 | down | topoisomerase(DNA)IIIalpha |
| 16811020 | 0.003010719 | 1.5680715 | down | alpha-andgamma-adaptinbindingprotein |
| 16889375 | 0.022375805 | 1.5679427 | down | NIF3NGG1interactingfactor3-like1(S.cerevisiae) |
| 16784367 | 0.020049704 | 1.567689 | down | suppressorofcytokinesignaling4 |
| 16809748 | 0.009689574 | 1.5675609 | down | meiosis-specificnuclearstructural1 |
| 16837754 | 0.014339547 | 1.5673659 | down | mitochondrialribosomalproteinS7 |
| 16966393 | 0.024685139 | 1.5670882 | down | solutecarrierfamily30(zinctransporter),member9 |
| 16825012 | 0.00542381 | 1.5667055 | down | ubiquitinspecificpeptidase31 |
| 16948021 | 0.009960732 | 1.5666558 | down | epithelialcelltransformingsequence2oncogene |
| 16814083 | 0.002811846 | 1.5663733 | down | smallnuclearribonucleoprotein25kDa(U11/U12) |
| 17014169 | 0.027292168 | 1.5660542 | down | tubbylikeprotein4 |
| 16774771 | 0.002387867 | 1.5658206 | down | cytidineanddCMPdeaminasedomaincontaining1 |
| 16811129 | 0.013231322 | 1.565693 | down | acidic(leucine-rich)nuclearphosphoprotein32family,memberA |
| 16746857 | 0.02733818 | 1.5652275 | down | uncharacterizedLOC100507424\|integrinalphaFG-GAPrepeatcontaining2 |
| 16941871 | 0.04113953 | 1.5650731 | down | interleukin17receptorB |
| 17036160 | 0.00187429 | 1.5648171 | down | zincfingerprotein57homolog(mouse) |
| 17031413 | 0.001787777 | 1.5647614 | down | zincfingerprotein57homolog(mouse) |
| 16804022 | 0.004412951 | 1.5646884 | down |  |
| 16846504 | 0.027491517 | 1.5646335 | down | familywithsequencesimilarity117,memberA |
| 16833449 | 0.04777509 | 1.5644398 | down | phosphatidylinositolglycananchorbiosynthesis,classW |
| 17117756 | 0.043163296 | 1.5643829 | down | familywithsequencesimilarity136,memberApseudogene |
| 16928046 | 0.005190521 | 1.5642047 | down | SWI/SNFrelated,matrixassociated,actindependentregulatorofchromatin,subfamilyb,member1 |
| 16883546 | 0.005270553 | 1.5641654 | down |  |
| 16979060 | 0.008407403 | 1.5638585 | down | chromosome4openreadingframe21 |
| 16878335 | 0.007135723 | 1.5636492 | down | zincfingerprotein512 |
| 16887561 | 0.003501498 | 1.5636164 | down | DDB1andCUL4associatedfactor17 |
| 16864806 | 0.049366437 | 1.5633849 | down | zincfingerprotein766 |
| 16929347 | 0.021941395 | 1.5627013 | down | tyrosine3-monooxygenase/tryptophan5-monooxygenaseactivationprotein,etapolypeptide |
| 16956002 | 5.29E-04 | 1.5625979 | down | EGFdomain-specificO-linkedN-acetylglucosamine(GlcNAc)transferase |
| 16663317 | 0.01444691 | 1.5625876 | down | Yboxbindingprotein1 |
| 16848453 | 0.008371207 | 1.5621847 | down | solutecarrierfamily39(metaliontransporter),member11 |
| 17064939 | 3.15E-04 | 1.5619777 | down | non-SMCcondensinIIcomplex,subunitG2 |
| 16662134 | 4.01E-04 | 1.561381 | down | retinoblastomabindingprotein4\|retinoblastomabindingprotein4pseudogene1 |
| 16920910 | 0.028810397 | 1.561297 | down | proteasome(prosome,macropain)subunit,alphatype,7 |
| 17051620 | 0.003055977 | 1.5608221 | down |  |
| 16958100 | 0.035465367 | 1.5607549 | down | karyopherinalpha1(importinalpha5) |
| 16803185 | 0.04428741 | 1.5605465 | down | COMMdomaincontaining4 |
| 16799776 | 0.005357747 | 1.5605294 | down | OIP5antisenseRNA1(non-proteincoding) |
| 16961551 | 0.035567928 | 1.5604956 | down | phospholipaseD1,phosphatidylcholine-specific |
| 17038911 | 0.001950637 | 1.5604664 | down | zincfingerprotein57homolog(mouse) |
| 17019280 | 0.015307508 | 1.5603137 | down | male-enhancedantigen1 |
| 17093595 | 0.00206716 | 1.5602665 | down | Fanconianemia,complementationgroupG |
| 16740797 | 0.004810683 | 1.5595415 | down | mitochondrialribosomalproteinL11 |
| 16664118 | 9.76E-04 | 1.5593998 | down | nuclearautoantigenicspermprotein(histone-binding) |
| 16868564 | 0.011340522 | 1.5592806 | down | eukaryotictranslationinitiationfactor3,subunitG |
| 17041350 | 0.024662934 | 1.5590357 | down | ribosomalproteinS18 |
| 17063047 | 5.06E-04 | 1.558898 | down | coiled-coil-helix-coiled-coil-helixdomaincontaining3 |
| 17000465 | 0.046269055 | 1.5587854 | down | eukaryotictranslationterminationfactor1 |
| 16809929 | 0.01722126 | 1.5587406 | down | SAFB-like,transcriptionmodulator |
| 16861852 | 0.030353613 | 1.5586177 | down | actinin,alpha4 |
| 17076063 | 0.006719257 | 1.5584904 | down | glutathionereductase |
| 16721965 | 0.014034078 | 1.558105 | down | Ctr9,Paf1/RNApolymeraseIIcomplexcomponent,homolog(S.cerevisiae) |
| 16901427 | 0.012743837 | 1.5579759 | down | UDP-glucuronatedecarboxylase1 |
| 16919769 | 9.77E-04 | 1.5576936 | down | nuclearreceptorcoactivator5 |
| 17016739 | 0.001677396 | 1.55751 | down | zincfingerprotein57homolog(mouse) |
| 16714269 | 6.29E-04 | 1.5574387 | down | translocaseofinnermitochondrialmembrane23homolog(yeast) |
| 16968675 | 0.03311071 | 1.556964 | down | heatshockprotein90kDaalpha(cytosolic),classBmember3,pseudogene |
| 17079220 | 0.03684435 | 1.5565262 | down | RAD54homologB(S.cerevisiae)\|fibrinogensilencerbindingprotein |
| 16818733 | 0.001018392 | 1.5553539 | down | HEATrepeatcontaining3 |
| 17033869 | 0.001963685 | 1.5553272 | down | zincfingerprotein57homolog(mouse) |
| 16682259 | 0.008617952 | 1.5553111 | down | ciliaryrootletcoiled-coil,rootletinpseudogene2 |
| 16821021 | 0.006098159 | 1.5548205 | down | proteasome(prosome,macropain)26Ssubunit,non-ATPase,7 |
| 16853042 | 0.029088026 | 1.5548205 | down | translocaseofinnermitochondrialmembrane21homolog(yeast) |
| 16882658 | 0.03364749 | 1.5546981 | down | ribose5-phosphateisomeraseA |
| 16834634 | 0.033189714 | 1.5544592 | down | DEAH(Asp-Glu-Ala-His)boxpolypeptide8 |
| 16763512 | 0.010117124 | 1.5544395 | down | solutecarrierfamily38,member1 |
| 16723447 | 0.030546471 | 1.5537844 | down |  |
| 16916546 | 1.54E-04 | 1.553594 | down | smallnuclearribonucleoproteinpolypeptidesBandB1 |
| 17106587 | 0.008121495 | 1.5534841 | down | ubiquitin-conjugatingenzymeE2A |
| 16877272 | 0.004494878 | 1.5530096 | down |  |
| 16817811 | 0.020449406 | 1.5520463 | down | proteinphosphatase4,catalyticsubunit |
| 16784498 | 0.009826317 | 1.5517075 | down | pellinoE3ubiquitinproteinligasefamilymember2 |
| 16804205 | 5.02E-04 | 1.5512856 | down | zincfingerandSCANdomaincontaining2 |
| 16678611 | 0.006125805 | 1.5502831 | down | componentofoligomericgolgicomplex2 |
| 16961466 | 0.008495851 | 1.5499258 | down | ribosomalproteinL22-like1 |
| 16962995 | 0.019698156 | 1.5493532 | down | largesubunitGTPase1homolog(S.cerevisiae) |
| 16663743 | 0.017719507 | 1.5492878 | down | importin13 |
| 16788142 | 0.024943467 | 1.5491763 | down | vacciniarelatedkinase1 |
| 16840293 | 0.004803545 | 1.5489095 | down | DEAH(Asp-Glu-Ala-His)boxpolypeptide33 |
| 16985304 | 0.020953415 | 1.548825 | down | chromosome5openreadingframe44 |
| 16894361 | 0.019407364 | 1.5486314 | down | tyrosine3-monooxygenase/tryptophan5-monooxygenaseactivationprotein,thetapolypeptide |
| 16753964 | 0.028672475 | 1.5483987 | down | chaperonincontainingTCP1,subunit2(beta) |
| 17025297 | 0.005525352 | 1.5483615 | down | t-complex1\|smallnucleolarRNA,H/ACAbox29 |
| 16716547 | 0.014595691 | 1.5482427 | down | insulin-degradingenzyme |
| 16669121 | 9.68E-04 | 1.5481371 | down | ATPase,Na+/K+transporting,alpha1polypeptide |
| 16811832 | 0.044859614 | 1.5480713 | down | snurportin1 |
| 16682930 | 0.006026298 | 1.5477351 | down | eukaryotictranslationinitiationfactor4gamma,3 |
| 17071041 | 0.007285274 | 1.5474851 | down | NADHdehydrogenase(ubiquinone)complexI,assemblyfactor6\|uncharacterizedLOC100506538 |
| 16829835 | 0.012684505 | 1.5474026 | down | arrestin,beta2 |
| 16821398 | 0.009866929 | 1.5473244 | down | heatshockfactorbindingprotein1 |
| 16914743 | 0.029528618 | 1.546914 | down | smallnucleolarRNA,C/Dbox12B |
| 17067970 | 0.03364118 | 1.5468361 | down | ash2(absent,small,orhomeotic)-like(Drosophila) |
| 16826038 | 4.96E-04 | 1.5465902 | down | coldshockdomainproteinApseudogene1 |
| 16888270 | 0.006066212 | 1.5461462 | down | integrin,alpha4(antigenCD49D,alpha4subunitofVLA-4receptor) |
| 16749826 | 0.011689655 | 1.5457022 | down | dynamin1-like |
| 17019831 | 0.020344622 | 1.5456407 | down | methylmalonylCoAmutase |
| 17077994 | 0.046285257 | 1.5449013 | down |  |
| 16700630 | 0.046315692 | 1.5445516 | down | TAR(HIV-1)RNAbindingprotein1 |
| 16774917 | 0.015754895 | 1.5443285 | down | ribonucleaseH2,subunitB |
| 16990083 | 0.006817382 | 1.5443184 | down | solutecarrierfamily35,memberA4 |
| 16889935 | 2.50E-04 | 1.5443183 | down |  |
| 17098187 | 0.019942107 | 1.5441484 | down | spermatidperinuclearRNAbindingprotein |
| 16759469 | 0.040469054 | 1.542795 | down | smallnucleolarRNA,H/ACAbox49 |
| 16823097 | 0.020984838 | 1.5427638 | down | RNAbindingproteinS1,serine-richdomain |
| 16697043 | 0.0386966 | 1.5426438 | down | DEAH(Asp-Glu-Ala-His)boxpolypeptide9\|uncharacterizedLOC647070 |
| 16664215 | 0.013503323 | 1.5425996 | down |  |
| 17015975 | 0.043342404 | 1.5421218 | down | thiopurineS-methyltransferase |
| 16657680 | 0.003115105 | 1.5420687 | down | UDP-Gal:betaGalbeta1,3-galactosyltransferasepolypeptide6 |
| 16825592 | 0.029425832 | 1.5419981 | down | HIRAinteractingprotein3 |
| 17098464 | 0.035783567 | 1.5417403 | down | non-proteincodingRNA,repressorofNFAT |
| 16969591 | 0.006190254 | 1.5416445 | down | hydroxyacyl-CoAdehydrogenase |
| 16978334 | 6.87E-04 | 1.5416348 | down | H2Ahistonefamily,memberZ |
| 16948871 | 0.002154953 | 1.5413908 | down | proteasome(prosome,macropain)26Ssubunit,non-ATPase,2 |
| 16725735 | 2.76E-04 | 1.5413474 | down | flapstructure-specificendonuclease1 |
| 16706200 | 0.017304685 | 1.5413144 | down | vinculin |
| 16695944 | 0.022332683 | 1.5412918 | down | transcriptionaladaptor1 |
| 16876849 | 0.00899832 | 1.5407466 | down | ArfGAPwithSH3domain,ankyrinrepeatandPHdomain2 |
| 16935767 | 0.011114399 | 1.5405848 | down | malonylCoA:ACPacyltransferase(mitochondrial) |
| 16804236 | 0.046220925 | 1.540114 | down | zincfingerprotein592 |
| 16939023 | 0.011522986 | 1.5395467 | down | CTD(carboxy-terminaldomain,RNApolymeraseII,polypeptideA)smallphosphatase-like |
| 16858235 | 0.019940041 | 1.5393271 | down | interleukinenhancerbindingfactor3,90kDa |
| 16993291 | 0.006955967 | 1.5390954 | down | zincfingerprotein354B |
| 16692656 | 0.010064025 | 1.5386118 | down | splicingfactor3b,subunit4,49kDa |
| 16936933 | 0.007472965 | 1.5385442 | down | SETdomainandmarinertransposasefusiongene |
| 16732088 | 0.029604763 | 1.5381333 | down | traffickingproteinparticlecomplex4\|microRNA3656 |
| 16704351 | 0.011758756 | 1.5380038 | down | zincfingerprotein22(KOX15) |
| 16847621 | 0.04012049 | 1.5377364 | down | FtsJhomolog3(E.coli) |
| 16754177 | 0.015767202 | 1.5366135 | down | transmembraneprotein19 |
| 16833963 | 0.014655049 | 1.536207 | down | smallnucleolarRNA,C/Dbox124 |
| 16995938 | 0.00832006 | 1.5361605 | down | chromosome5openreadingframe34 |
| 16854316 | 0.04641095 | 1.5359799 | down | oxysterolbindingprotein-like1A |
| 17048388 | 0.00570462 | 1.5358046 | down | coiled-coildomaincontaining132 |
| 16733045 | 0.006142822 | 1.5356396 | down | etoposideinduced2.4mRNA |
| 16854016 | 0.011815096 | 1.5354416 | down | spirehomolog1(Drosophila) |
| 16738023 | 0.04139166 | 1.535224 | down | proteasome(prosome,macropain)26Ssubunit,ATPase,3 |
| 16786941 | 0.001071208 | 1.5349638 | down | AHA1,activatorofheatshock90kDaproteinATPasehomolog1(yeast) |
| 16980245 | 0.023756279 | 1.534751 | down | glycophorinE(MNSbloodgroup) |
| 17053769 | 0.028063208 | 1.533485 | down |  |
| 16815971 | 0.001955268 | 1.5333339 | down | excisionrepaircross-complementingrodentrepairdeficiency,complementationgroup4 |
| 16700421 | 0.009616326 | 1.5332378 | down | tetratricopeptiderepeatdomain13 |
| 16870931 | 0.04680791 | 1.5328397 | down | importin5pseudogene |
| 16781136 | 0.037643373 | 1.532621 | down | tubulin,gammacomplexassociatedprotein3 |
| 16917689 | 0.003062313 | 1.53262 | down | RalGTPaseactivatingprotein,alphasubunit2(catalytic) |
| 16941693 | 0.015302889 | 1.5324314 | down | signalpeptidasecomplexsubunit1homolog(S.cerevisiae) |
| 16675844 | 0.00668399 | 1.5323728 | down | importin9 |
| 16896961 | 0.04444742 | 1.5319241 | down | thyroidadenomaassociated |
| 16881274 | 0.004897018 | 1.5318234 | down | M-phasephosphoprotein10(U3smallnucleolarribonucleoprotein) |
| 16730306 | 0.010463862 | 1.5317743 | down | serine/arginine-richsplicingfactor8 |
| 17114114 | 0.001020509 | 1.5314947 | down | ecto-NOXdisulfide-thiolexchanger2 |
| 17046886 | 0.017780304 | 1.5313222 | down | generaltranscriptionfactorIIi,pseudogene1\|generaltranscriptionfactorII,i,pseudogene |
| 16816601 | 0.033112325 | 1.5306954 | down | acyl-CoAsynthetasemedium-chainfamilymember3 |
| 16878406 | 0.02625458 | 1.5302817 | down | mitochondrialribosomalproteinL33 |
| 17107559 | 9.90E-04 | 1.5299463 | down | AF4/FMR2family,member2 |
| 16793225 | 0.003169121 | 1.5297719 | down | discs,large(Drosophila)homolog-associatedprotein5 |
| 16761269 | 0.012941419 | 1.528607 | down | C-typelectindomainfamily7,memberA |
| 17080614 | 0.005763566 | 1.5285571 | down | mitochondrialribosomalproteinL13\|uncharacterizedLOC100506980 |
| 16755173 | 0.035509594 | 1.5283394 | down | plexinC1 |
| 16825196 | 0.024087522 | 1.5282619 | down | non-SMCelement1homolog(S.cerevisiae) |
| 17071412 | 0.008754183 | 1.5281986 | down | spermassociatedantigen1 |
| 16948909 | 1.66E-05 | 1.5281591 | down | eukaryotictranslationinitiationfactor4gamma,1 |
| 16836232 | 0.018913027 | 1.527997 | down | syntaxinbindingprotein4 |
| 16989054 | 0.046714805 | 1.5279049 | down |  |
| 16694728 | 0.007156565 | 1.5269325 | down | mitochondrialribosomalproteinL24 |
| 17058731 | 0.019174365 | 1.5269016 | down | generaltranscriptionfactorIIi,pseudogene1\|generaltranscriptionfactorII,i,pseudogene |
| 16766403 | 0.018679416 | 1.5260475 | down | transmembraneprotein194A |
| 16947107 | 0.005692716 | 1.5255635 | down | purinergicreceptorP2Y,G-proteincoupled,1 |
| 16685609 | 0.014331903 | 1.5253885 | down |  |
| 16745597 | 0.025122847 | 1.525349 | down | zincfingerprotein202 |
| 16968848 | 0.017858965 | 1.5251269 | down | tiggertransposableelementderived2 |
| 16690139 | 0.034485098 | 1.5250208 | down | exostoses(multiple)-like2 |
| 16717394 | 0.04214884 | 1.5249747 | down |  |
| 16821280 | 0.03219143 | 1.5249661 | down | gigaxonin |
| 16758624 | 0.004227927 | 1.5248712 | down | DEAD(Asp-Glu-Ala-Asp)boxpolypeptide55 |
| 16778959 | 0.038731348 | 1.5246891 | down | succinate-CoAligase,ADP-forming,betasubunit |
| 16775056 | 0.039904155 | 1.5235941 | down | heterogeneousnuclearribonucleoproteinA1-like2 |
| 17005740 | 0.034197725 | 1.523412 | down | HLAcomplexgroup11(non-proteincoding) |
| 16711383 | 0.031207372 | 1.5233132 | down | tubulin,alpha-like3 |
| 16718769 | 0.010500899 | 1.5232642 | down | PDZdomaincontaining8 |
| 16935646 | 0.04344612 | 1.5224469 | down |  |
| 17002337 | 0.014669137 | 1.5222776 | down |  |
| 16823193 | 0.003190588 | 1.5221671 | down | transcriptionelongationfactorB(SIII),polypeptide2(18kDa,elonginB) |
| 16901894 | 0.037178468 | 1.5220915 | down | zincfingerCCCH-typecontaining8 |
| 16977970 | 0.010333107 | 1.5219097 | down | GPRINfamilymember3 |
| 16743056 | 0.040807907 | 1.5218787 | down | malicenzyme3,NADP(+)-dependent,mitochondrial |
| 16943548 | 0.024761865 | 1.5217327 | down | activatedleukocytecelladhesionmolecule |
| 16972850 | 0.02124996 | 1.5213865 | down | coiled-coildomaincontaining111 |
| 16934786 | 0.014220044 | 1.5198238 | down |  |
| 16685281 | 7.29E-04 | 1.5197195 | down | mitochondrialribosomalproteinS15 |
| 16988450 | 0.005769707 | 1.5194552 | down | serumresponsefactorbindingprotein1 |
| 16748751 | 0.002033388 | 1.5186266 | down | serine/threoninekinasereceptorassociatedprotein\|uncharacterizedLOC100653179\|uncharacterizedLOC100653013 |
| 16887289 | 0.032909963 | 1.5185125 | down | peptidylprolylisomeraseG(cyclophilinG) |
| 16723468 | 0.005662565 | 1.518175 | down | cellcycleassociatedprotein1 |
| 16745767 | 0.00203964 | 1.5178038 | down | transmembraneprotein218 |
| 16840262 | 0.001891478 | 1.5177944 | down | nucleoporin88kDa |
| 17047611 | 0.002588644 | 1.5177394 | down | PMS2postmeioticsegregationincreased2(S.cerevisiae)pseudogene |
| 16959465 | 0.017358417 | 1.5177282 | down | anaphasepromotingcomplexsubunit13 |
| 17058651 | 0.003333964 | 1.5176867 | down | stromalantigen3-like2 |
| 17096205 | 0.03131566 | 1.5174544 | down | zincfingerprotein367 |
| 16769159 | 0.007102574 | 1.5174276 | down | N-acetylglucosamine-1-phosphatetransferase,alphaandbetasubunits |
| 16830182 | 0.001434881 | 1.5173674 | down | thioredoxindomaincontaining17 |
| 16918011 | 1.04E-04 | 1.5168633 | down | chromosome20openreadingframe3 |
| 17111260 | 0.039430294 | 1.5166776 | down |  |
| 16834764 | 0.011668026 | 1.5165114 | down |  |
| 16819082 | 0.00887459 | 1.5161041 | down | lysophosphatidylcholineacyltransferase2 |
| 16950895 | 0.014991793 | 1.515138 | down | ribosomalproteinL32 |
| 16713085 | 0.024368493 | 1.5146439 | down | kinesinfamilymember5B |
| 16821330 | 0.008279571 | 1.5145386 | down | phospholipaseC,gamma2(phosphatidylinositol-specific) |
| 16855283 | 0.01361549 | 1.5145324 | down | CXXCfingerprotein1 |
| 16776188 | 0.001533862 | 1.5143766 | down | tripeptidylpeptidaseII |
| 16895431 | 0.041274477 | 1.5143664 | down |  |
| 16670847 | 0.02621541 | 1.5139326 | down |  |
| 17067755 | 0.012121447 | 1.5133389 | down |  |
| 16695907 | 0.010647531 | 1.5130588 | down | transmembraneandcoiled-coildomains1 |
| 16768478 | 0.002389774 | 1.5130191 | down | earlyendosomeantigen1 |
| 17010246 | 0.019398717 | 1.5129256 | down | potassiumvoltage-gatedchannel,KQT-likesubfamily,member5 |
| 16876950 | 0.034054436 | 1.5119404 | down | TATAboxbindingprotein(TBP)-associatedfactor,RNApolymeraseI,B,63kDa |
| 17100390 | 7.26E-05 | 1.5118847 | down | transmembraneprotein203 |
| 16941952 | 0.014646941 | 1.5118015 | down | coiled-coildomaincontaining66 |
| 16662900 | 0.01490003 | 1.5117943 | down |  |
| 16846476 | 9.54E-04 | 1.5114324 | down | solutecarrierfamily35,memberB1 |
| 16806834 | 0.024354544 | 1.5112958 | down | ERmembraneproteincomplexsubunit7 |
| 16792798 | 0.005847991 | 1.5105786 | down | salvadorhomolog1(Drosophila) |
| 17061108 | 0.034685835 | 1.5099431 | down | RASp21proteinactivator4\|RASp21proteinactivator4C,pseudogene\|RASp21proteinactivator4B\|uroplakin3B-like |
| 16863737 | 0.008068605 | 1.5093069 | down |  |
| 16668730 | 0.001797012 | 1.5089785 | down | DEAD(Asp-Glu-Ala-Asp)boxpolypeptide20 |
| 16708049 | 0.003079175 | 1.5089767 | down | MARVELdomaincontaining1 |
| 16837061 | 0.011945711 | 1.5089488 | down | smallnucleolarRNA,C/Dbox104 |
| 16807027 | 0.044901047 | 1.5084842 | down | aquariushomolog(mouse) |
| 16905514 | 0.035384126 | 1.508379 | down |  |
| 16933591 | 0.001454108 | 1.5072563 | down | adaptor-relatedproteincomplex1,beta1subunit |
| 16900737 | 0.005913451 | 1.5070735 | down | ankyrinrepeatdomain36B |
| 16776856 | 0.015693782 | 1.5069867 | down | lysosomal-associatedmembraneprotein1 |
| 16935751 | 4.31E-04 | 1.5068781 | down | tubulintyrosineligase-likefamily,member1 |
| 16778493 | 0.01449964 | 1.5065585 | down |  |
| 16866985 | 3.65E-04 | 1.5065062 | down | solutecarrierfamily39(zinctransporter),member3 |
| 17043856 | 0.001707117 | 1.5064607 | down | arylhydrocarbonreceptor |
| 16723832 | 0.003839593 | 1.505879 | down | apoptosisinhibitor5 |
| 16755878 | 0.008996489 | 1.5058254 | down | cholinephosphotransferase1 |
| 16692724 | 0.044642445 | 1.5056164 | down | acidic(leucine-rich)nuclearphosphoprotein32family,memberE |
| 16789953 | 0.026992178 | 1.5047734 | down | smallnucleolarRNA,C/Dbox126 |
| 16907960 | 0.034554664 | 1.504349 | down | BRCA1associatedRINGdomain1 |
| 16659171 | 0.013619147 | 1.5043218 | down | mitofusin2 |
| 16939863 | 0.026337707 | 1.5041517 | down | chromosome3openreadingframe23 |
| 17070120 | 0.033278354 | 1.5037404 | down | cysteine-richsecretoryproteinLCCLdomaincontaining1 |
| 16687445 | 0.004181027 | 1.5034736 | down | Yip1domainfamily,member1 |
| 16752305 | 0.005476154 | 1.5034306 | down | cyclin-dependentkinase2 |
| 16976158 | 0.025141079 | 1.5032682 | down | phosphoribosylpyrophosphateamidotransferase |
| 16988861 | 0.012963263 | 1.5031834 | down | isochorismatasedomaincontaining1 |
| 16708468 | 0.019125255 | 1.5026855 | down | beta-transducinrepeatcontainingE3ubiquitinproteinligase |
| 16702147 | 0.041569553 | 1.5025048 | down | RNAbindingmotifprotein17 |
| 16715031 | 0.018620078 | 1.5022684 | down | solutecarrierfamily25(mitochondrialcarrier |
| 17011966 | 0.041174415 | 1.5021743 | down | RWDdomaincontaining1 |
| 16700289 | 0.002455617 | 1.501854 | down | nucleoporin133kDa |
| 17115505 | 0.014831336 | 1.5016968 | down | filaminA,alpha |
| 16658904 | 0.016586434 | 1.5013566 | down |  |
| 17003050 | 0.003060908 | 1.5009347 | down |  |
| 17109706 | 0.03597725 | 1.5006903 | down | eukaryotictranslationinitiationfactor1A,X-linked |
| 16700034 | 0.003613981 | 1.5005211 | down | lin-9homolog(C.elegans) |
| 17049103 | 0.016179053 | 1.5005091 | down | COP9constitutivephotomorphogenichomologsubunit6(Arabidopsis) |
| 16678231 | 0.028956756 | 1.5003597 | down |  |
| 17092252 | 0.002866454 | 1.5002711 | down | RANbindingprotein6 |

**rNK versus untreated FC1.5 p<0.05**

| **Transcript Cluster Id** | **p value** | **FC (abs)** | **Regulation** | **Gene Description** |
| --- | --- | --- | --- | --- |
| 16977045 | 0.017769916 | 77.87659 | up | chemokine(C-X-Cmotif)ligand9 |
| 16885116 | 0.027132038 | 10.575573 | up |  |
| 17074313 | 0.04128986 | 8.150738 | up | defensin,alpha1B |
| 17074322 | 0.041698508 | 7.870333 | up | defensin,alpha1B |
| 16689332 | 0.047296844 | 7.6799583 | up | guanylatebindingprotein1,interferon-inducible |
| 16808708 | 0.014366162 | 7.143064 | up |  |
| 16800662 | 0.02002616 | 5.139975 | up |  |
| 16976827 | 0.017387742 | 4.759786 | up | chemokine(C-X-Cmotif)ligand5 |
| 16726880 | 5.30E-04 | 4.743591 | up |  |
| 17093536 | 0.035020612 | 4.7282944 | up | chemokine(C-Cmotif)ligand19 |
| 16836624 | 0.002703153 | 4.434107 | up | microRNA21 |
| 17076726 | 0.017439704 | 4.059862 | up | plasminogenactivator,tissue |
| 16800642 | 0.021221545 | 3.9796307 | up | highmobilitygroupnucleosomalbindingdomain2pseudogene46 |
| 16786650 | 0.001048182 | 3.937867 | up | RNA,5Sribosomal387 |
| 16808696 | 0.015264223 | 3.81821 | up | solutecarrierfamily30(zinctransporter),member4 |
| 16752132 | 0.013216789 | 3.8083954 | up | mucin-like1 |
| 16906534 | 0.009088473 | 3.7811959 | up | signaltransducerandactivatoroftranscription1,91kDa |
| 16999776 | 0.012888817 | 3.5451055 | up | interferonregulatoryfactor1 |
| 16859763 | 0.018136749 | 3.4466944 | up | interferon,gamma-inducibleprotein30 |
| 16922606 | 2.71E-04 | 3.4051702 | up |  |
| 16842659 | 4.02E-05 | 3.1950235 | up | aldolaseC,fructose-bisphosphate |
| 16860971 | 0.044974364 | 3.1658285 | up | hepcidinantimicrobialpeptide |
| 17051159 | 0.002369523 | 3.159224 | up | hypoxiainduciblelipiddroplet-associated |
| 16693406 | 0.005183137 | 3.1022785 | up |  |
| 17039977 | 0.02351892 | 3.0755155 | up | transporter1,ATP-bindingcassette,sub-familyB(MDR/TAP) |
| 17017979 | 0.023637144 | 3.0655887 | up | transporter1,ATP-bindingcassette,sub-familyB(MDR/TAP) |
| 17032476 | 0.023710957 | 3.059446 | up | transporter1,ATP-bindingcassette,sub-familyB(MDR/TAP) |
| 17027144 | 0.023714395 | 3.0573893 | up | transporter1,ATP-bindingcassette,sub-familyB(MDR/TAP) |
| 17034791 | 0.023687495 | 3.0570984 | up | transporter1,ATP-bindingcassette,sub-familyB(MDR/TAP) |
| 17037271 | 0.02376716 | 3.054837 | up | transporter1,ATP-bindingcassette,sub-familyB(MDR/TAP) |
| 17029788 | 0.023927031 | 3.050278 | up | transporter1,ATP-bindingcassette,sub-familyB(MDR/TAP) |
| 16774310 | 0.019344894 | 3.042505 | up |  |
| 16918516 | 0.047425613 | 2.9902403 | up |  |
| 17042487 | 0.024190366 | 2.9379222 | up | transporter1,ATP-bindingcassette,sub-familyB(MDR/TAP) |
| 16998044 | 4.09E-04 | 2.8686085 | up |  |
| 17063977 | 0.004537367 | 2.8033936 | up | familywithsequencesimilarity115,memberCpseudogene |
| 16761776 | 0.003979773 | 2.7975912 | up |  |
| 16671139 | 0.012402956 | 2.761531 | up | S100calciumbindingproteinA9 |
| 16977190 | 0.005244 | 2.7561858 | up |  |
| 16794145 | 0.018688155 | 2.7332768 | up |  |
| 16745561 | 0.009297184 | 2.7204921 | up | smallnucleolarRNA,C/Dbox14E |
| 16693414 | 0.009747786 | 2.7077627 | up | S100calciumbindingproteinA8 |
| 17106305 | 0.005281829 | 2.704188 | up |  |
| 16720085 | 0.025625244 | 2.6884542 | up | interferoninducedtransmembraneprotein1 |
| 16884918 | 2.89E-04 | 2.6373837 | up | insulininducedgene2 |
| 16981516 | 0.001925986 | 2.5925999 | up |  |
| 16671137 | 0.032868814 | 2.584088 | up |  |
| 16990203 | 0.020974569 | 2.5669377 | up | vaultRNA1-3 |
| 16702226 | 7.52E-04 | 2.5640488 | up |  |
| 17070242 | 0.04973608 | 2.5509794 | up |  |
| 16986913 | 0.04288209 | 2.527672 | up | versican |
| 17013170 | 0.04105762 | 2.5228915 | up |  |
| 16786058 | 0.03289269 | 2.447333 | up | smallnucleolarRNA,C/Dbox56B |
| 16800657 | 0.04714282 | 2.4322362 | up |  |
| 16922603 | 0.029799277 | 2.4275572 | up |  |
| 16976821 | 0.04438631 | 2.4274495 | up | pro-plateletbasicprotein(chemokine(C-X-Cmotif)ligand7) |
| 16702541 | 0.008598362 | 2.4265323 | up |  |
| 16913196 | 0.039130945 | 2.4181557 | up |  |
| 16830461 | 7.55E-04 | 2.4138002 | up | fibroblastgrowthfactor11 |
| 17005586 | 0.004784922 | 2.398263 | up | histonecluster2,H4b\|histonecluster4,H4\|histonecluster2,H4a\|histonecluster1,H4l\|histonecluster1,H4e\|histonecluster1,H4b\|histonecluster1,H4h\|histonecluster1,H4c\|histonecluster1,H4j\|histonecluster1,H4k\|histonecluster1,H4f\|histonecluster1,H4d\|histonecluster1,H4a\|histonecluster1,H4i |
| 16698782 | 0.001175976 | 2.3981893 | up | microRNA29c |
| 16719644 | 0.002144028 | 2.3776066 | up | BCL2/adenovirusE1B19kDainteractingprotein3\|BCL2/adenovirusE1B19kDainteractingprotein3pseudogene1 |
| 17056857 | 0.010506989 | 2.372718 | up | Tcellreceptorgammavariable1(non-functional) |
| 16697243 | 0.007376324 | 2.362058 | up |  |
| 16727626 | 0.032269347 | 2.338778 | up | RNA,U7smallnuclear23pseudogene |
| 16793674 | 0.002038207 | 2.3373005 | up |  |
| 16843511 | 0.006494513 | 2.3341434 | up | chemokine(C-Cmotif)ligand5 |
| 16999169 | 0.024794027 | 2.3309577 | up |  |
| 17056813 | 0.019816691 | 2.317897 | up | TCRgammaalternatereadingframeprotein\|Tcellreceptorgammajoining2\|Tcellreceptorgammajoining1 |
| 17056823 | 0.020095993 | 2.313509 | up | TCRgammaalternatereadingframeprotein\|Tcellreceptorgammajoining2\|Tcellreceptorgammajoining1 |
| 16958124 | 0.043311045 | 2.3069394 | up | poly(ADP-ribose)polymerasefamily,member9 |
| 16891840 | 0.014406325 | 2.2974153 | up |  |
| 16931766 | 0.048872307 | 2.2669294 | up | kelchdomaincontaining7B |
| 16984010 | 0.006408109 | 2.261575 | up | interleukin7receptor |
| 17102129 | 0.03395287 | 2.2441084 | up | spermidine/spermineN1-acetyltransferase1 |
| 16697018 | 0.004982186 | 2.2252705 | up | regulatorofG-proteinsignaling16 |
| 16956448 | 0.001904074 | 2.2217581 | up | glucan(1,4-alpha-),branchingenzyme1 |
| 16905123 | 0.016555421 | 2.212218 | up |  |
| 16726081 | 0.016329337 | 2.1893313 | up | retinoicacidreceptorresponder(tazaroteneinduced)3 |
| 16944695 | 0.043405354 | 2.1786144 | up | poly(ADP-ribose)polymerasefamily,member14 |
| 17016360 | 1.94E-04 | 2.175679 | up | histonecluster1,H4b\|histonecluster2,H4b\|histonecluster4,H4\|histonecluster2,H4a\|histonecluster1,H4l\|histonecluster1,H4e\|histonecluster1,H4h\|histonecluster1,H4c\|histonecluster1,H4j\|histonecluster1,H4k\|histonecluster1,H4f\|histonecluster1,H4d\|histonecluster1,H4a\|histonecluster1,H4i |
| 17007962 | 0.02972404 | 2.1698632 | up |  |
| 16702215 | 0.004371119 | 2.16048 | up |  |
| 17100651 | 0.003201058 | 2.1467712 | up |  |
| 17113443 | 0.020245316 | 2.110114 | up |  |
| 17117752 | 0.002752479 | 2.098584 | up |  |
| 17046911 | 0.02370489 | 2.0960512 | up | neutrophilcytosolicfactor1Bpseudogene |
| 17103185 | 0.006463696 | 2.0926182 | up | TIMPmetallopeptidaseinhibitor1 |
| 17027130 | 0.012205166 | 2.085087 | up | proteasome(prosome,macropain)subunit,betatype,8(largemultifunctionalpeptidase7) |
| 17017965 | 0.011801667 | 2.080377 | up | proteasome(prosome,macropain)subunit,betatype,8(largemultifunctionalpeptidase7) |
| 17042473 | 0.011709536 | 2.0781817 | up | proteasome(prosome,macropain)subunit,betatype,8(largemultifunctionalpeptidase7) |
| 17032462 | 0.011984028 | 2.0742738 | up | proteasome(prosome,macropain)subunit,betatype,8(largemultifunctionalpeptidase7) |
| 17034777 | 0.012195047 | 2.0735443 | up | proteasome(prosome,macropain)subunit,betatype,8(largemultifunctionalpeptidase7) |
| 17052864 | 0.004219543 | 2.0721955 | up | familywithsequencesimilarity115,memberC\|familywithsequencesimilarity115,memberCpseudogene\|familywithsequencesimilarity115,memberD(pseudogene) |
| 17029774 | 0.012070986 | 2.0712247 | up | proteasome(prosome,macropain)subunit,betatype,8(largemultifunctionalpeptidase7) |
| 17059828 | 0.020402774 | 2.0712001 | up | tissuefactorpathwayinhibitor2 |
| 17039963 | 0.012593623 | 2.0678055 | up | proteasome(prosome,macropain)subunit,betatype,8(largemultifunctionalpeptidase7) |
| 16913075 | 0.01205828 | 2.0662925 | up | RNA,5Sribosomal483 |
| 16822637 | 0.00830792 | 2.058381 | up | tryptasebeta2(gene/pseudogene)\|tryptasealpha/beta1 |
| 17062945 | 0.03754198 | 2.0575433 | up |  |
| 16916600 | 0.016329382 | 2.054244 | up |  |
| 17037257 | 0.011287863 | 2.0526874 | up | proteasome(prosome,macropain)subunit,betatype,8(largemultifunctionalpeptidase7) |
| 16857519 | 0.009497579 | 2.0465915 | up | egf-likemodulecontaining,mucin-like,hormonereceptor-like1 |
| 17016390 | 0.002081507 | 2.0374436 | up | histonecluster1,H2bg\|histonecluster1,H2bc\|histonecluster1,H2bi\|histonecluster1,H2be\|histonecluster1,H2bf |
| 16678917 | 0.036292832 | 2.0352898 | up |  |
| 17056853 | 0.002890285 | 2.016464 | up | Tcellreceptorgammavariable2 |
| 17056849 | 0.016938968 | 2.0157933 | up | TCRgammaalternatereadingframeprotein\|Tcellreceptorgammavariable3 |
| 16760868 | 0.00195003 | 2.0134642 | up | solutecarrierfamily2(facilitatedglucosetransporter),member3 |
| 16689354 | 0.03144813 | 1.9890617 | up | guanylatebindingprotein2,interferon-inducible |
| 16693409 | 0.018652577 | 1.986117 | up | S100calciumbindingproteinA12 |
| 16782010 | 0.028582564 | 1.9859891 | up | Tcellreceptoralphajoining59(non-functional) |
| 17056830 | 0.044375654 | 1.985699 | up | Tcellreceptorgammavariable10(non-functional) |
| 17106242 | 0.027061097 | 1.9839317 | up | uncharacterizedLOC401613 |
| 16888979 | 0.018267108 | 1.9815502 | up |  |
| 16924756 | 0.017452987 | 1.9738619 | up | BACH1antisenseRNA1(non-proteincoding) |
| 16994693 | 0.02517513 | 1.9732468 | up |  |
| 17106307 | 0.029920356 | 1.9688296 | up |  |
| 16906612 | 0.013715056 | 1.9682508 | up |  |
| 17005072 | 0.009397415 | 1.9669532 | up |  |
| 17050348 | 0.025844403 | 1.9627535 | up |  |
| 16701599 | 0.01333103 | 1.9618106 | up | olfactoryreceptor,family11,subfamilyL,member1 |
| 16800660 | 0.02665047 | 1.9483472 | up |  |
| 16770780 | 0.002493688 | 1.9465799 | up |  |
| 17005858 | 0.027840892 | 1.9444176 | up | histonecluster1,H2ai\|histonecluster1,H2ah\|histonecluster1,H2ag\|histonecluster1,H2am\|histonecluster1,H2al\|histonecluster1,H2ak |
| 17117736 | 0.017535366 | 1.937132 | up |  |
| 16850216 | 0.01376075 | 1.9360776 | up | secretedandtransmembrane1 |
| 16889455 | 0.0250841 | 1.930859 | up | caspase10,apoptosis-relatedcysteinepeptidase |
| 16684142 | 0.026443398 | 1.9284608 | up | RNA,U7smallnuclear29pseudogene\|RNA,U7smallnuclear45pseudogene |
| 16765537 | 0.030627819 | 1.913271 | up | Gprotein-coupledreceptor84 |
| 16943336 | 0.043224603 | 1.9069873 | up | transmembraneprotein45A |
| 17072019 | 0.044447877 | 1.902082 | up |  |
| 17106312 | 0.01264724 | 1.9005877 | up |  |
| 17031373 | 0.024586635 | 1.8996717 | up | ubiquitinD\|gamma-aminobutyricacid(GABA)Breceptor,1 |
| 17023377 | 0.008984009 | 1.8986162 | up | RNA,5Sribosomal217 |
| 17112184 | 0.002757659 | 1.8980131 | up |  |
| 16695121 | 0.001956194 | 1.8926014 | up | absentinmelanoma2 |
| 16724861 | 0.04277291 | 1.8914059 | up | serpinpeptidaseinhibitor,cladeG(C1inhibitor),member1 |
| 16924521 | 0.011194805 | 1.8875444 | up | longintergenicnon-proteincodingRNA515 |
| 16904667 | 0.037064698 | 1.87017 | up | sodiumchannel,voltage-gated,typeIX,alphasubunit |
| 16672478 | 0.043131992 | 1.8641424 | up | SLAMfamilymember8 |
| 16823866 | 0.031575385 | 1.8600737 | up | suppressorofcytokinesignaling1 |
| 17005582 | 0.02596415 | 1.8533751 | up | histonecluster1,H2bc\|histonecluster1,H2bi\|histonecluster1,H2be\|histonecluster1,H2bf\|histonecluster1,H2bg |
| 16741897 | 0.023343286 | 1.8533425 | up |  |
| 16929615 | 0.0278356 | 1.8526525 | up | apolipoproteinL,6 |
| 16910851 | 0.018707335 | 1.8522912 | up |  |
| 17009424 | 0.01721439 | 1.8472996 | up |  |
| 16959925 | 0.03628153 | 1.8443961 | up | 5'-3'exoribonuclease1 |
| 16889450 | 0.022619229 | 1.8361484 | up | RNA,U7smallnuclear45pseudogene\|RNA,U7smallnuclear29pseudogene |
| 16849400 | 0.006921251 | 1.8342721 | up | suppressorofcytokinesignaling3 |
| 16870200 | 0.04856673 | 1.8333384 | up | bonemarrowstromalcellantigen2 |
| 16872602 | 0.031651836 | 1.8288866 | up | carcinoembryonicantigen-relatedcelladhesionmolecule4 |
| 16769865 | 0.02613495 | 1.8283713 | up | USP30antisenseRNA1(non-proteincoding) |
| 16945470 | 0.03181723 | 1.8281528 | up |  |
| 17056787 | 0.021375952 | 1.8263813 | up |  |
| 16888963 | 0.00487863 | 1.8253984 | up | nucleicacidbindingprotein1 |
| 16921578 | 6.29E-04 | 1.8245877 | up |  |
| 16830577 | 0.015049754 | 1.8232648 | up |  |
| 16984801 | 2.69E-04 | 1.8136278 | up | granzymeA(granzyme1,cytotoxicT-lymphocyte-associatedserineesterase3) |
| 16819355 | 0.042614944 | 1.8110509 | up | NLRfamily,CARDdomaincontaining5 |
| 16843372 | 0.032855157 | 1.8040618 | up |  |
| 17001242 | 0.038661163 | 1.80304 | up |  |
| 16669796 | 0.002005078 | 1.800452 | up | thioredoxininteractingprotein |
| 16802038 | 0.04981199 | 1.79956 | up |  |
| 17064285 | 0.032833822 | 1.7960314 | up | transmembraneprotein176B |
| 17113545 | 0.023620715 | 1.7936742 | up |  |
| 16819244 | 0.04889548 | 1.7917887 | up | metallothionein1C,pseudogene |
| 17027101 | 0.013423073 | 1.7907072 | up | transporter2,ATP-bindingcassette,sub-familyB(MDR/TAP) |
| 17042443 | 0.009055289 | 1.7900426 | up | transporter2,ATP-bindingcassette,sub-familyB(MDR/TAP) |
| 16804490 | 0.012941998 | 1.7900178 | up | interferonstimulatedexonucleasegene20kDa |
| 16782003 | 0.04067891 | 1.7898511 | up | Tcellreceptordeltaconstant |
| 16699456 | 0.008654757 | 1.7892563 | up | microRNA194-1 |
| 16663005 | 0.00236907 | 1.7874342 | up |  |
| 17108703 | 0.004614573 | 1.7869276 | up | RNA,5Sribosomal498 |
| 17104406 | 0.046840906 | 1.7866114 | up | RNA,5Sribosomal506 |
| 17037228 | 0.010584541 | 1.7862656 | up | transporter2,ATP-bindingcassette,sub-familyB(MDR/TAP) |
| 16800667 | 0.006321657 | 1.7818146 | up |  |
| 16944654 | 0.005910822 | 1.7817256 | up | familywithsequencesimilarity162,memberA |
| 17105047 | 0.002941084 | 1.7793778 | up | phosphoglyceratekinase1\|uncharacterizedLOC100653302\|uncharacterizedLOC100652805 |
| 17032433 | 0.008710569 | 1.7743155 | up | transporter2,ATP-bindingcassette,sub-familyB(MDR/TAP) |
| 16973756 | 0.003753268 | 1.7716643 | up |  |
| 16738544 | 0.01966744 | 1.7670997 | up | ubiquitin-conjugatingenzymeE2L6 |
| 16694176 | 0.026593424 | 1.764627 | up |  |
| 16777963 | 0.023786128 | 1.7623658 | up |  |
| 17011412 | 0.046791915 | 1.7578658 | up |  |
| 16793475 | 0.014759177 | 1.7564327 | up | Gprotein-coupledreceptor135 |
| 16967368 | 0.02041019 | 1.7526339 | up | UDPglucuronosyltransferase2family,polypeptideB28 |
| 16843602 | 0.039620295 | 1.7466727 | up | chemokine(C-Cmotif)ligand3-like3\|chemokine(C-Cmotif)ligand3-like1 |
| 17029745 | 0.006515672 | 1.739037 | up | transporter2,ATP-bindingcassette,sub-familyB(MDR/TAP) |
| 17058392 | 0.006148362 | 1.7381339 | up |  |
| 16875340 | 0.04801168 | 1.7369815 | up | leukocyteimmunoglobulin-likereceptor,subfamilyB(withTMandITIMdomains),member3\|leukocyteimmunoglobulin-likereceptor,subfamilyA(withTMdomain),member6\|uncharacterizedLOC100291689 |
| 17047367 | 0.006204039 | 1.7358159 | up |  |
| 16758052 | 0.046472106 | 1.7336086 | up | purinergicreceptorP2X,ligand-gatedionchannel,7 |
| 17063973 | 0.012085971 | 1.7292575 | up |  |
| 16715384 | 0.019234607 | 1.7289001 | up |  |
| 17084417 | 0.03869059 | 1.727447 | up |  |
| 16949442 | 0.045067728 | 1.7260989 | up | receptor(chemosensory)transporterprotein4 |
| 16998039 | 0.006440214 | 1.7250264 | up |  |
| 17117443 | 0.01407959 | 1.7232064 | up |  |
| 16692597 | 0.00166998 | 1.7180848 | up | uncharacterizedLOC388692\|uncharacterizedLOC440570 |
| 16726886 | 0.014597818 | 1.7174711 | up |  |
| 17070061 | 0.027173541 | 1.713489 | up | lymphocyteantigen96 |
| 16692570 | 0.001570918 | 1.7131903 | up | uncharacterizedLOC388692\|uncharacterizedLOC440570 |
| 17081401 | 0.001305001 | 1.7084138 | up | N-mycdownstreamregulated1 |
| 16701398 | 0.003283432 | 1.7082586 | up | SMYD3intronictranscript1(non-proteincoding) |
| 16785142 | 0.016489113 | 1.7079028 | up |  |
| 17007307 | 0.02588049 | 1.7024542 | up | proteasome(prosome,macropain)subunit,betatype,9(largemultifunctionalpeptidase2) |
| 16887736 | 0.007502988 | 1.7024065 | up | pyruvatedehydrogenasekinase,isozyme1 |
| 17052857 | 0.012395181 | 1.6985812 | up |  |
| 16696575 | 0.001560617 | 1.696909 | up | RNA,5Sribosomal68 |
| 16786801 | 0.005987288 | 1.6949081 | up | vasohibin1 |
| 17011939 | 0.034921408 | 1.6919713 | up | familywithsequencesimilarity26,memberF |
| 16997199 | 0.037704803 | 1.6904888 | up | ectodermal-neuralcortex1(withBTB-likedomain) |
| 16917632 | 0.03036344 | 1.6878504 | up |  |
| 17005655 | 0.017456666 | 1.6858754 | up | butyrophilin,subfamily3,memberA1 |
| 16692553 | 0.005508591 | 1.6855572 | up |  |
| 16769440 | 0.002391503 | 1.6840698 | up | phosphoglyceratemutase1(brain)\|phosphoglyceratemutasefamilymember4 |
| 16709128 | 0.032034606 | 1.6814755 | up | dualspecificityphosphatase5 |
| 16874828 | 0.015467865 | 1.6799724 | up | naturalkillercellgroup7sequence |
| 17066897 | 0.003112146 | 1.6796553 | up | solutecarrierfamily25(mitochondrialirontransporter),member37 |
| 16702175 | 0.015824609 | 1.677035 | up | 6-phosphofructo-2-kinase/fructose-2,6-biphosphatase3 |
| 16912597 | 0.019928264 | 1.6745622 | up | DNA(cytosine-5-)-methyltransferase3beta |
| 16996810 | 0.012011249 | 1.672464 | up | MAST4antisenseRNA1(non-proteincoding) |
| 16976674 | 0.03355828 | 1.6706489 | up |  |
| 16721280 | 0.043655902 | 1.6679223 | up | tripartitemotifcontaining22 |
| 16679480 | 0.04378968 | 1.6634165 | up | microRNA4677 |
| 16662584 | 0.003540248 | 1.6593584 | up | SH3domaincontaining21\|uncharacterizedLOC100127947 |
| 17012370 | 0.009622036 | 1.6578488 | up |  |
| 16762686 | 0.01298109 | 1.6570841 | up |  |
| 16819202 | 0.015263672 | 1.6547893 | up | metallothionein3 |
| 16829570 | 0.005917913 | 1.6523347 | up | serpinpeptidaseinhibitor,cladeF(alpha-2antiplasmin,pigmentepitheliumderivedfactor),member1 |
| 16798071 | 0.001081957 | 1.6502405 | up |  |
| 17000208 | 0.04981286 | 1.649359 | up | vaultRNA2-1 |
| 16778731 | 0.029782902 | 1.647927 | up | smallnucleolarRNA,H/ACAbox31 |
| 16913617 | 0.034253508 | 1.645708 | up | microRNA548o-2 |
| 17100655 | 0.002214416 | 1.643563 | up |  |
| 17063975 | 0.028893769 | 1.6432844 | up | CTAGEfamily,member6,pseudogene\|CTAGEfamily,member15,pseudogene |
| 16802942 | 0.044729307 | 1.6401538 | up |  |
| 17084363 | 0.027922243 | 1.6385994 | up | annexinA2pseudogene2 |
| 16838455 | 0.018578146 | 1.6382229 | up |  |
| 16672596 | 0.023979548 | 1.6323748 | up | SUMO1pseudogene3 |
| 17033661 | 0.02921314 | 1.6307906 | up | proteasome(prosome,macropain)subunit,betatype,9(largemultifunctionalpeptidase2) |
| 17038644 | 0.028421236 | 1.6290598 | up | proteasome(prosome,macropain)subunit,betatype,9(largemultifunctionalpeptidase2) |
| 16780271 | 0.023195686 | 1.6273934 | up | ATP-bindingcassette,sub-familyC(CFTR/MRP),member4 |
| 16860709 | 0.002066109 | 1.6251204 | up | glucose-6-phosphateisomerase |
| 16852622 | 0.009232872 | 1.6219858 | up | mucosaassociatedlymphoidtissuelymphomatranslocationgene1 |
| 16772811 | 0.0105443 | 1.618353 | up | zincfingerprotein605 |
| 17028360 | 0.030208396 | 1.609856 | up | proteasome(prosome,macropain)subunit,betatype,9(largemultifunctionalpeptidase2) |
| 16998848 | 0.020603282 | 1.6077609 | up |  |
| 16972155 | 0.021716693 | 1.6035575 | up | methylsterolmonooxygenase1 |
| 16843627 | 0.03203604 | 1.5988892 | up | chemokine(C-Cmotif)ligand3-like3\|chemokine(C-Cmotif)ligand3-like1 |
| 16960618 | 0.037900604 | 1.5984176 | up | transmembraneprotein14E |
| 16962022 | 0.016908323 | 1.5975919 | up | lysosomal-associatedmembraneprotein3 |
| 17079513 | 0.029320477 | 1.5955007 | up |  |
| 16922819 | 0.043202806 | 1.5947361 | up |  |
| 16827483 | 0.04360182 | 1.5929912 | up | proteasome(prosome,macropain)subunit,betatype,10 |
| 16859509 | 0.0173444 | 1.5920926 | up | familywithsequencesimilarity125,memberA\|uncharacterizedLOC100507535 |
| 16962584 | 0.027595205 | 1.5910084 | up | B-cellCLL/lymphoma6 |
| 16925452 | 0.0047131 | 1.5888205 | up | claudin14 |
| 16873060 | 0.019047946 | 1.5852975 | up | plasminogenactivator,urokinasereceptor |
| 16891254 | 0.003346242 | 1.5847572 | up |  |
| 16708489 | 0.009729545 | 1.5840361 | up | deletedinprimaryciliarydyskinesiahomolog(mouse) |
| 16976833 | 0.009596402 | 1.5826583 | up | chemokine(C-X-Cmotif)ligand3 |
| 16986866 | 0.020621076 | 1.5817873 | up | ATPase,H+transporting,lysosomalaccessoryprotein1-like\|uncharacterizedLOC645079 |
| 17076493 | 0.0052311 | 1.5798788 | up |  |
| 16948569 | 0.02831655 | 1.5786302 | up | KLHL6antisenseRNA1(non-proteincoding) |
| 16985440 | 5.88E-04 | 1.5778215 | up | microtubuleassociatedserine/threoninekinasefamilymember4 |
| 16693142 | 0.031171277 | 1.576949 | up |  |
| 16751246 | 0.031899523 | 1.5765617 | up |  |
| 16974244 | 0.018470332 | 1.5759453 | up | microRNA548i-2 |
| 16997858 | 0.007151366 | 1.5722456 | up |  |
| 16974521 | 0.034946837 | 1.5714066 | up |  |
| 17117684 | 0.004704906 | 1.5711571 | up | sortingnexin29pseudogene2\|nuclearporecomplexinteractingprotein-like3 |
| 16681408 | 0.038134433 | 1.5710428 | up | solutecarrierfamily2(facilitatedglucose/fructosetransporter),member5 |
| 16996953 | 0.026031582 | 1.570767 | up | baculoviralIAPrepeat-containingprotein1-like |
| 16832421 | 0.041220956 | 1.5687003 | up |  |
| 16691730 | 6.69E-05 | 1.5686347 | up | FcfragmentofIgG,highaffinityIb,receptor(CD64)\|FcfragmentofIgG,highaffinityIc,receptor(CD64),pseudogene |
| 16793259 | 0.02031182 | 1.5679932 | up |  |
| 16863589 | 0.003553434 | 1.5678494 | up | complementcomponent5areceptor1 |
| 16835213 | 2.15E-04 | 1.5674412 | up | aminopeptidasepuromycinsensitive\|uncharacterizedLOC100653042 |
| 16777992 | 0.013322479 | 1.5662662 | up | mitochondrialinnermembraneorganizingsystem1pseudogene1 |
| 16706257 | 0.03354326 | 1.5605422 | up |  |
| 16757603 | 0.04291552 | 1.5583637 | up | longintergenicnon-proteincodingRNA173 |
| 16918662 | 0.022894798 | 1.5563959 | up | metallothionein1pseudogene3 |
| 16984968 | 0.006704512 | 1.5557317 | up |  |
| 16998037 | 0.007626055 | 1.5557009 | up |  |
| 17088991 | 0.001324522 | 1.5552546 | up | MIR181A2hostgene(non-proteincoding) |
| 16929631 | 0.016670592 | 1.5534971 | up | apolipoproteinL,1 |
| 16915712 | 0.021464335 | 1.5510434 | up | solutecarrierorganicaniontransporterfamily,member4A1 |
| 16901200 | 0.014137137 | 1.5425532 | up | ringfingerprotein149\|smallnucleolarRNA,C/Dbox89 |
| 16681989 | 0.00181652 | 1.5415275 | up |  |
| 17055261 | 0.003771174 | 1.5413598 | up |  |
| 17040615 | 0.029729413 | 1.5411322 | up | immediateearlyresponse3\|uncharacterizedLOC100294307 |
| 17033122 | 0.028891588 | 1.5403045 | up | immediateearlyresponse3\|uncharacterizedLOC100294307 |
| 17006392 | 0.028964853 | 1.540284 | up |  |
| 17027572 | 0.029716015 | 1.5401881 | up | immediateearlyresponse3\|uncharacterizedLOC100294307 |
| 16721278 | 0.009875468 | 1.5388178 | up | tripartitemotifcontaining78,pseudogene |
| 16741864 | 0.00497457 | 1.538052 | up | FCHanddoubleSH3domains2 |
| 16782637 | 0.02444847 | 1.5372282 | up |  |
| 17030417 | 0.031372692 | 1.5359098 | up | immediateearlyresponse3\|uncharacterizedLOC100294307 |
| 16850286 | 0.022535095 | 1.5357052 | up | microRNA4525 |
| 16743554 | 0.014006356 | 1.5355474 | up | RNA,5Sribosomal346 |
| 16734670 | 0.04636768 | 1.5350271 | up |  |
| 16934709 | 2.54E-04 | 1.5346189 | up | interleukin2receptor,beta |
| 17010872 | 0.027025467 | 1.5321572 | up | chromosome6openreadingframe165 |
| 16774545 | 0.003607678 | 1.5317998 | up |  |
| 17115850 | 0.015591544 | 1.5304571 | up | colonystimulatingfactor2receptor,alpha,low-affinity(granulocyte-macrophage) |
| 16872223 | 0.026088268 | 1.5295141 | up |  |
| 16847418 | 0.048618592 | 1.5273578 | up |  |
| 16949827 | 0.03614465 | 1.5243764 | up | XXYLT1antisenseRNA2(non-proteincoding) |
| 17053535 | 0.013218093 | 1.5182666 | up | acid-sensing(proton-gated)ionchannel3\|ATP-bindingcassette,sub-familyB(MDR/TAP),member8 |
| 17079910 | 0.017216794 | 1.5181903 | up | Kruppel-likefactor10 |
| 16843578 | 0.031693596 | 1.5130302 | up | chemokine(C-Cmotif)ligand3 |
| 17021382 | 0.02775919 | 1.5129743 | up |  |
| 16960114 | 0.012197262 | 1.5114291 | up | procollagen-lysine,2-oxoglutarate5-dioxygenase2 |
| 16922028 | 0.043406732 | 1.5110285 | up |  |
| 17074612 | 0.004222625 | 1.5103478 | up |  |
| 16679742 | 0.03802336 | 1.510219 | up | tripartitemotifcontaining58\|olfactoryreceptor,family2,subfamilyW,member3 |
| 16811372 | 0.009620925 | 1.5082176 | up | pyruvatekinase,muscle |
| 16872405 | 0.037505306 | 1.50819 | up |  |
| 16813871 | 0.021612873 | 1.5072982 | up | chondroitinsulfatesynthase1 |
| 17016379 | 0.016790427 | 1.5066729 | up | histonecluster1,H2bc\|histonecluster1,H2bi\|histonecluster1,H2be\|histonecluster1,H2bf\|histonecluster1,H2bg |
| 16818975 | 0.025600381 | 1.5065857 | up | RNA,5Sribosomal427 |
| 16787151 | 0.03995913 | 1.5059077 | up |  |
| 16905127 | 0.01412098 | 1.5049801 | up |  |
| 16949261 | 0.040813085 | 1.5041625 | up |  |
| 16787342 | 0.02559423 | 1.5024475 | up |  |
| 16755171 | 0.004819559 | 1.5009737 | up |  |
| 16704055 | 0.013974468 | 1.5003331 | up | hydroxysteroid(17-beta)dehydrogenase7pseudogene2 |
| 16681370 | 0.029493038 | 1.5001997 | up | enolase1,(alpha) |

| **Transcript Cluster Id** | **p value** | **FC (abs)** | **Regulation** | **Gene Description** |
| --- | --- | --- | --- | --- |
| 16926605 | 0.00585194 | 4.895132 | down |  |
| 16672435 | 0.0022245 | 3.5877059 | down | FcfragmentofIgE,highaffinityI,receptorfor |
| 17026568 | 0.011570944 | 3.1654081 | down | zincfingerprotein57homolog(mouse) |
| 17086193 | 0.002816812 | 3.0839136 | down | phosphoserineaminotransferase1 |
| 16976131 | 0.035739124 | 2.7919865 | down | RNA,5Sribosomal361\|RNA,5Sribosomal162 |
| 16869291 | 0.013751128 | 2.152957 | down | smallnucleolarRNA,C/Dbox41 |
| 17117665 | 0.045322903 | 2.1005886 | down |  |
| 16722299 | 0.011233272 | 2.0807023 | down | phosphodiesterase3B,cGMP-inhibited |
| 16931197 | 0.00270998 | 1.9867113 | down | familywithsequencesimilarity118,memberA |
| 16976192 | 0.03230723 | 1.9793811 | down | serinepeptidaseinhibitor,Kazaltype2(acrosin-trypsininhibitor) |
| 16681812 | 0.042056397 | 1.9632928 | down | RNA,U5Esmallnuclear4,pseudogene |
| 16983765 | 0.012967035 | 1.9632597 | down | natriureticpeptidereceptorC/guanylatecyclaseC(atrionatriureticpeptidereceptorC) |
| 17034339 | 0.034253154 | 1.9554423 | down | lymphotoxinbeta(TNFsuperfamily,member3) |
| 16806584 | 0.002296922 | 1.9552172 | down |  |
| 17053769 | 0.02729582 | 1.953471 | down |  |
| 17031867 | 0.03405678 | 1.9533128 | down | lymphotoxinbeta(TNFsuperfamily,member3) |
| 17026762 | 0.03403845 | 1.9527258 | down | lymphotoxinbeta(TNFsuperfamily,member3) |
| 17036587 | 0.03424976 | 1.9507655 | down | lymphotoxinbeta(TNFsuperfamily,member3) |
| 17029082 | 0.034473814 | 1.9502676 | down | lymphotoxinbeta(TNFsuperfamily,member3) |
| 17041863 | 0.034397822 | 1.9471858 | down | lymphotoxinbeta(TNFsuperfamily,member3) |
| 17039380 | 0.034630403 | 1.9469056 | down | lymphotoxinbeta(TNFsuperfamily,member3) |
| 17078529 | 0.003597538 | 1.9195426 | down |  |
| 16829967 | 0.005992991 | 1.9191308 | down | glycoproteinIb(platelet),alphapolypeptide |
| 17096423 | 0.014025813 | 1.9120662 | down | hemogen |
| 16752217 | 0.007523361 | 1.8908805 | down | growthdifferentiationfactor11 |
| 16692290 | 0.029193068 | 1.8850263 | down | phosphodiesterase4Dinteractingproteinpseudogene |
| 16789782 | 0.044553604 | 1.8667244 | down |  |
| 17012859 | 0.022678846 | 1.86434 | down | phosphodiesterase7B |
| 16836260 | 0.010747435 | 1.8545479 | down | hepaticleukemiafactor |
| 16838249 | 0.003329431 | 1.8465575 | down |  |
| 16906419 | 0.012161845 | 1.8384395 | down | solutecarrierfamily40(iron-regulatedtransporter),member1 |
| 16819539 | 0.028410394 | 1.8290519 | down | Gprotein-coupledreceptor56 |
| 17041494 | 0.005304545 | 1.8244601 | down | zincfingerprotein57homolog(mouse) |
| 17069728 | 0.036881797 | 1.8226554 | down | phosphatidylinositol-3,4,5-trisphosphate-dependentRacexchangefactor2 |
| 16923890 | 0.004385466 | 1.8199738 | down | collagen,typeVI,alpha2 |
| 16947287 | 0.011047595 | 1.8148521 | down | TCDD-induciblepoly(ADP-ribose)polymerase |
| 16712357 | 0.007362308 | 1.7942568 | down |  |
| 16797403 | 0.021183357 | 1.785883 | down | immunoglobulinheavyconstantmu |
| 16745798 | 0.035303153 | 1.7852122 | down | fasciculationandelongationproteinzeta1(zyginI) |
| 16720799 | 0.005274323 | 1.7634543 | down |  |
| 17031413 | 0.003991557 | 1.7416826 | down | zincfingerprotein57homolog(mouse) |
| 16926027 | 0.009327462 | 1.7406176 | down |  |
| 16721835 | 0.007380026 | 1.7392042 | down |  |
| 17036160 | 0.003903956 | 1.7389181 | down | zincfingerprotein57homolog(mouse) |
| 17038911 | 0.004012014 | 1.7346088 | down | zincfingerprotein57homolog(mouse) |
| 16799423 | 0.015845977 | 1.7345886 | down |  |
| 17047459 | 0.04541421 | 1.7341 | down | smallnucleolarRNA,H/ACAbox14A |
| 17033869 | 0.004233566 | 1.7319589 | down | zincfingerprotein57homolog(mouse) |
| 17016739 | 0.003918188 | 1.729308 | down | zincfingerprotein57homolog(mouse) |
| 17109775 | 0.005717031 | 1.7275326 | down |  |
| 17078626 | 0.001097954 | 1.723361 | down | solutecarrierfamily10(sodium/bileacidcotransporterfamily),member5 |
| 16781814 | 0.03045651 | 1.7148379 | down |  |
| 16674953 | 5.69E-04 | 1.7110343 | down | tRNAsplicingendonuclease15homolog(S.cerevisiae) |
| 16726876 | 0.03439836 | 1.7110012 | down |  |
| 16780808 | 0.016695663 | 1.7070755 | down | KDEL(Lys-Asp-Glu-Leu)containing1 |
| 16979825 | 4.22E-04 | 1.7015605 | down | poly(A)bindingprotein,cytoplasmic4-like |
| 16757886 | 0.025114508 | 1.6957499 | down | cytochromecoxidasesubunitVIapolypeptide1 |
| 16900605 | 0.021170307 | 1.6929938 | down |  |
| 16662427 | 0.039229553 | 1.6894693 | down |  |
| 16980946 | 0.004252054 | 1.6872393 | down | plateletderivedgrowthfactorC |
| 16711383 | 0.004861116 | 1.6836284 | down | tubulin,alpha-like3 |
| 17043843 | 0.009392266 | 1.6822494 | down | tetraspanin13 |
| 17063571 | 0.0352479 | 1.6646101 | down |  |
| 16705701 | 0.003961169 | 1.6622765 | down | H2Ahistonefamily,memberY2 |
| 16876939 | 0.007257694 | 1.6616415 | down |  |
| 16924878 | 0.001383749 | 1.6594869 | down | T-celllymphomainvasionandmetastasis1 |
| 16980051 | 0.04856932 | 1.6582278 | down | calmegin |
| 17096904 | 0.001361572 | 1.6566466 | down | catenin(cadherin-associatedprotein),alpha-like1 |
| 17028632 | 0.011966794 | 1.6556596 | down | zincfingerprotein57homolog(mouse) |
| 17087517 | 0.045091186 | 1.6521702 | down | nuclearreceptorsubfamily4,groupA,member3 |
| 16738258 | 0.04762135 | 1.6474321 | down |  |
| 16791810 | 0.017677477 | 1.6444911 | down | ARHGAP5antisenseRNA1(non-proteincoding) |
| 17105076 | 0.02481423 | 1.6418813 | down | purinergicreceptorP2Y,G-proteincoupled,10 |
| 16834764 | 0.012029305 | 1.6404507 | down |  |
| 16741584 | 4.46E-04 | 1.6357992 | down |  |
| 16793750 | 0.007761615 | 1.6328019 | down | sphingosine-1-phosphatephosphatase1 |
| 16808433 | 0.001481502 | 1.6319871 | down | protein(peptidylprolylcis/transisomerase)NIMA-interacting,4pseudogene1 |
| 16919044 | 0.029708987 | 1.6312287 | down | retinoblastoma-like1(p107) |
| 16887840 | 0.014348856 | 1.6267917 | down | celldivisioncycleassociated7 |
| 17052652 | 0.036713395 | 1.6260706 | down | protease,serine,1(trypsin1)\|protease,serine,3pseudogene2 |
| 16911149 | 0.003156867 | 1.6236814 | down | PCNAantisenseRNA1(non-proteincoding) |
| 16664215 | 0.010283499 | 1.6233557 | down |  |
| 16768341 | 0.002653209 | 1.6195536 | down | ATPase,Ca++transporting,plasmamembrane1 |
| 17115796 | 0.006319081 | 1.6164178 | down | microRNA1184-1\|microRNA1184-2\|microRNA1184-3 |
| 17115763 | 0.006387143 | 1.6158798 | down | microRNA1184-1\|microRNA1184-2\|microRNA1184-3 |
| 17108585 | 0.00650742 | 1.615002 | down | microRNA1184-1\|microRNA1184-2\|microRNA1184-3 |
| 16797415 | 0.034585442 | 1.6130592 | down | immunoglobulinheavyconstantalpha1\|immunoglobulinheavyconstantmu\|immunoglobulinheavyconstantalpha2(A2mmarker)\|immunoglobulinheavylocus\|immunoglobulinheavyjoining3 |
| 16802162 | 0.008298184 | 1.610924 | down | DIS3mitoticcontrolhomolog(S.cerevisiae)-like |
| 16814863 | 0.003093884 | 1.6106642 | down | smallnucleolarRNA,H/ACAbox78 |
| 16812738 | 0.001416103 | 1.6078587 | down | hepatoma-derivedgrowthfactor,relatedprotein3 |
| 16826532 | 0.004378492 | 1.6030545 | down |  |
| 17109335 | 0.001978923 | 1.6030376 | down |  |
| 17062255 | 0.03987208 | 1.597865 | down | familywithsequencesimilarity3,memberC |
| 16828886 | 0.042095967 | 1.5973828 | down | GINScomplexsubunit2(Psf2homolog) |
| 17046524 | 0.026054803 | 1.59711 | down |  |
| 16758242 | 0.012700661 | 1.5942899 | down | B-cellCLL/lymphoma7A |
| 16966897 | 0.04669132 | 1.5921022 | down | steroid5alpha-reductase3 |
| 16685848 | 0.020898668 | 1.5916315 | down |  |
| 16841768 | 0.03408473 | 1.5909507 | down | centromereproteinV |
| 16697196 | 0.020223822 | 1.5882003 | down | familywithsequencesimilarity129,memberA |
| 16856350 | 0.006340294 | 1.5880767 | down | calponin2 |
| 16788398 | 0.028701318 | 1.5877799 | down |  |
| 17013851 | 0.031113252 | 1.5871195 | down | myctarget1 |
| 17019683 | 0.032251287 | 1.5856593 | down |  |
| 16689113 | 0.02265467 | 1.5844388 | down | collagen,typeXXIV,alpha1 |
| 16929573 | 0.007475853 | 1.5834484 | down | minichromosomemaintenancecomplexcomponent5 |
| 16846714 | 0.003621576 | 1.5821521 | down | ankyrinrepeatdomain40 |
| 16845567 | 0.007906855 | 1.5746502 | down | chromosome17openreadingframe65 |
| 17065652 | 0.041009724 | 1.5740633 | down | methioninesulfoxidereductaseA |
| 16880122 | 0.035283595 | 1.5718879 | down | spectrin,beta,non-erythrocytic1 |
| 16702047 | 0.023044055 | 1.5633012 | down | neuroepithelialcelltransforming1 |
| 16740317 | 0.028567655 | 1.5547104 | down |  |
| 16665447 | 0.022452964 | 1.5519795 | down | ubiquitinspecificpeptidase1 |
| 17114220 | 0.002182641 | 1.5492917 | down | muscleblind-likesplicingregulator3 |
| 16916958 | 0.014190502 | 1.5435836 | down | proliferatingcellnuclearantigen |
| 16682259 | 0.036496982 | 1.5426749 | down | ciliaryrootletcoiled-coil,rootletinpseudogene2 |
| 16879883 | 0.03294874 | 1.541668 | down | mutShomolog2,coloncancer,nonpolyposistype1(E.coli) |
| 17060412 | 0.02596058 | 1.5416273 | down | minichromosomemaintenancecomplexcomponent7 |
| 16663075 | 0.022608979 | 1.5401121 | down | defectsinmorphology1homolog(S.cerevisiae) |
| 16701106 | 0.01362397 | 1.5394131 | down | choroideremia-like(Rabescortprotein2) |
| 16971737 | 0.002761156 | 1.5393757 | down | guanylatecyclase1,soluble,beta3 |
| 16973857 | 0.029059058 | 1.5385373 | down | familywithsequencesimilarity86,memberE,pseudogene |
| 16842266 | 0.009003142 | 1.5380825 | down | microfibrillar-associatedprotein4 |
| 16658889 | 0.003865877 | 1.5380158 | down | apoptosis-inducing,TAF9-likedomain1\|APITD1-CORTreadthrough\|cortistatin |
| 16900090 | 0.00267173 | 1.5372328 | down | immunoglobulinkappalocus\|immunoglobulinkappaconstant\|uncharacterizedLOC100294406 |
| 16972553 | 0.005078053 | 1.5322737 | down | WDrepeatdomain17 |
| 17095703 | 0.04666449 | 1.5320284 | down | nuclearfactor,interleukin3regulated |
| 16712292 | 0.015516065 | 1.5296671 | down | proteintyrosinephosphatase-like(prolineinsteadofcatalyticarginine),memberA |
| 16753426 | 0.029793715 | 1.5294765 | down | SLIT-ROBORhoGTPaseactivatingprotein1 |
| 16718983 | 1.99E-04 | 1.5290978 | down | minichromosomemaintenancecomplexbindingprotein |
| 16816364 | 0.04131947 | 1.5287231 | down |  |
| 17115505 | 0.002452877 | 1.5282216 | down | filaminA,alpha |
| 16773076 | 0.002253269 | 1.5272532 | down | longintergenicnon-proteincodingRNA539 |
| 16895179 | 0.017494103 | 1.5262325 | down | tumorproteinp53inducibleprotein3 |
| 17079448 | 0.008866873 | 1.5256712 | down | NIPA-likedomaincontaining2 |
| 16938562 | 0.008901117 | 1.5252968 | down | transforminggrowthfactor,betareceptorII(70/80kDa) |
| 16844872 | 0.007153549 | 1.5232517 | down | junctionplakoglobin |
| 16792810 | 0.004548466 | 1.5225053 | down | ninein(GSK3Binteractingprotein) |
| 16947173 | 0.003955463 | 1.5223854 | down | membranemetallo-endopeptidase |
| 17115801 | 0.00205979 | 1.5206931 | down | coagulationfactorVIII-associated3\|coagulationfactorVIII-associated2\|coagulationfactorVIII-associated1 |
| 17108582 | 0.002071573 | 1.5201523 | down | coagulationfactorVIII-associated3\|coagulationfactorVIII-associated2\|coagulationfactorVIII-associated1 |
| 16754966 | 0.013584054 | 1.5194286 | down |  |
| 16974581 | 0.01310485 | 1.5179716 | down |  |
| 16736807 | 0.03144121 | 1.5179313 | down | coiled-coildomaincontaining34 |
| 17076963 | 0.039674144 | 1.5176525 | down |  |
| 17111858 | 0.008282167 | 1.5150267 | down | solutecarrierfamily7(cationicaminoacidtransporter,y+system),member3 |
| 16903897 | 0.04936782 | 1.5145273 | down | nuclearreceptorsubfamily4,groupA,member2 |
| 16658184 | 0.003700031 | 1.5108161 | down | tumorproteinp63regulated1-like |
| 17114567 | 0.021091172 | 1.5094087 | down | smallnucleolarRNA,C/Dbox61\|RNAbindingmotifprotein,X-linked |
| 16842452 | 0.002637529 | 1.5082083 | down | ubiquitinspecificpeptidase22 |
| 16915993 | 0.001140649 | 1.5080634 | down | pancreaticprogenitorcelldifferentiationandproliferationfactorhomolog(zebrafish) |
| 16826692 | 0.009763919 | 1.5068274 | down | autocrinemotilityfactorreceptor,E3ubiquitinproteinligase |
| 17102789 | 0.028210592 | 1.5061179 | down | CASKantisenseRNA1(non-proteincoding) |
| 17101248 | 0.02085942 | 1.5048248 | down | ARSDantisenseRNA1(non-proteincoding) |
| 16871613 | 0.002712597 | 1.5047548 | down | polymerase(RNA)II(DNAdirected)polypeptideI,14.5kDa |
| 16778611 | 0.02135706 | 1.5044726 | down | coiled-coildomaincontaining122 |
| 17100820 | 0.004542769 | 1.5043285 | down |  |
| 17100824 | 0.004546168 | 1.5040772 | down |  |
| 16780069 | 0.0456598 | 1.5038579 | down | sproutyhomolog2(Drosophila) |
| 16773946 | 0.004753583 | 1.5008758 | down | replicationfactorC(activator1)3,38kDa |
