# Supplementary material for: Natural Killer Cells Improve Hematopoietic Stem Cell Engraftment by Increasing Stem Cell Clonogenicity In Vitro and in a Humanized Mouse Model
Source: PLoS One. 2015 Oct 14;10(10):e0138623. doi: 10.1371/journal.pone.0138623 (PMC4605799; doi:10.1371/journal.pone.0138623)
Supplement: S2 Table — (DOCX) [file pone.0138623.s002.docx]

**rNK versus aNK FC1.5 p<0.05**

| **Transcript Cluster Id** | **p value** | **FC (abs)** | **Regulation** | **Gene Description** |
| --- | --- | --- | --- | --- |
| 16767247 | 0.04160391 | 10.17823 | up | interferon,gamma |
| 17030610 | 0.03277602 | 9.723695 | up | lymphotoxinalpha(TNFsuperfamily,member1) |
| 17027784 | 0.022765107 | 9.718365 | up | lymphotoxinalpha(TNFsuperfamily,member1) |
| 17038107 | 0.03279022 | 9.707645 | up | lymphotoxinalpha(TNFsuperfamily,member1) |
| 17035408 | 0.032853447 | 9.707032 | up | lymphotoxinalpha(TNFsuperfamily,member1) |
| 17040702 | 0.032762676 | 9.215528 | up | lymphotoxinalpha(TNFsuperfamily,member1) |
| 17033327 | 0.032800518 | 9.196816 | up | lymphotoxinalpha(TNFsuperfamily,member1) |
| 17006649 | 0.03276022 | 9.194144 | up | lymphotoxinalpha(TNFsuperfamily,member1) |
| 16990203 | 0.009762405 | 6.456399 | up | vaultRNA1-3 |
| 17102801 | 0.00162832 | 4.484479 | up | Gprotein-coupledreceptor82 |
| 17057422 | 0.013418341 | 4.23128 | up | smallnucleolarRNA,H/ACAbox9\|smallnucleolarRNAhostgene15(non-proteincoding) |
| 16746992 | 0.002509215 | 3.795439 | up | cyclinD2 |
| 16904278 | 0.005779179 | 3.565058 | up | dipeptidyl-peptidase4 |
| 16775643 | 0.021595936 | 3.399355 | up | Nedd4familyinteractingprotein2 |
| 16711501 | 0.03540616 | 3.257147 | up | interleukin2receptor,alpha |
| 16855673 | 0.001118921 | 3.161919 | up | B-cellCLL/lymphoma2 |
| 16954567 | 0.041844435 | 3.022447 | up | cytokineinducibleSH2-containingprotein |
| 16810933 | 4.45E-04 | 2.600071 | up | TIMELESSinteractingprotein |
| 16933760 | 0.020509174 | 2.598695 | up | leukemiainhibitoryfactor |
| 16768941 | 0.014205333 | 2.515762 | up | IKBKBinteractingprotein |
| 16798820 | 0.011363606 | 2.404908 | up |  |
| 16725041 | 0.004299273 | 2.400245 | up | familywithsequencesimilarity111,memberB |
| 16810970 | 0.020393215 | 2.370893 | up | smallnucleolarRNA,C/Dbox18C\|ribosomalproteinL4\|smallnucleolarRNA,C/Dbox18B\|smallnucleolarRNA,C/Dbox18A\|smallnucleolarRNA,C/Dbox16 |
| 16961616 | 0.024302348 | 2.276472 | up | tumornecrosisfactor(ligand)superfamily,member10 |
| 16812738 | 0.009581755 | 2.243221 | up | hepatoma-derivedgrowthfactor,relatedprotein3 |
| 17011694 | 0.003749728 | 2.219088 | up | ribosomeproductionfactor2homolog(S.cerevisiae) |
| 16989861 | 0.003216182 | 2.218182 | up |  |
| 16798919 | 0.014732875 | 2.204917 | up | RhoGTPaseactivatingprotein11A |
| 16780793 | 0.016928066 | 2.179852 | up | testisexpressed30 |
| 16756134 | 0.011065716 | 2.152962 | up |  |
| 17044403 | 0.03551486 | 2.115269 | up |  |
| 16834056 | 0.04966852 | 2.103941 | up | celldivisioncycle6homolog(S.cerevisiae) |
| 17020787 | 0.011777116 | 2.0782 | up | Mab-21domaincontaining1 |
| 16702229 | 0.02694075 | 2.022386 | up |  |
| 16783836 | 0.01637918 | 2.012981 | up | Fanconianemia,complementationgroupM |
| 16703858 | 0.003077479 | 2.012914 | up | cAMPresponsiveelementmodulator |
| 17117843 | 0.021991102 | 2.010837 | up |  |
| 16784226 | 0.0182935 | 2.007484 | up |  |
| 17109808 | 0.007570454 | 1.990486 | up | apolipoproteinO |
| 16715133 | 0.013682765 | 1.988464 | up | pyrophosphatase(inorganic)1 |
| 16813319 | 0.027523052 | 1.981414 | up |  |
| 16793460 | 0.04563341 | 1.979367 | up | translocaseofinnermitochondrialmembrane9homolog(yeast) |
| 16855065 | 0.047661427 | 1.956845 | up | immediateearlyresponse3interactingprotein1 |
| 16698023 | 8.31E-04 | 1.937434 | up | ubiquitin-conjugatingenzymeE2T(putative) |
| 17072222 | 0.023650723 | 1.924228 | up |  |
| 17017224 | 0.003681119 | 1.917111 | up | lymphotoxinbeta(TNFsuperfamily,member3) |
| 16718983 | 0.047368784 | 1.898585 | up | minichromosomemaintenancecomplexbindingprotein |
| 16784272 | 0.009281545 | 1.88628 | up |  |
| 16886919 | 0.008545741 | 1.873913 | up | proteasome(prosome,macropain)26Ssubunit,non-ATPase,14 |
| 17005396 | 0.016368672 | 1.873223 | up | geminin,DNAreplicationinhibitor |
| 16793052 | 0.0218018 | 1.872576 | up | glucosamine-phosphateN-acetyltransferase1 |
| 16804902 | 3.95E-04 | 1.867459 | up | Bloomsyndrome,RecQhelicase-like |
| 16864129 | 0.004539818 | 1.867144 | up | fms-relatedtyrosinekinase3ligand |
| 16810974 | 0.03779928 | 1.853935 | up | smallnucleolarRNA,C/Dbox16\|smallnucleolarRNA,C/Dbox18C\|smallnucleolarRNA,C/Dbox18B\|smallnucleolarRNA,C/Dbox18A\|ribosomalproteinL4 |
| 16783905 | 0.015551373 | 1.847301 | up | leucinerichrepeatprotein1 |
| 16960186 | 0.041325636 | 1.832673 | up | phospholipidscramblase1 |
| 17004843 | 0.008894132 | 1.827727 | up |  |
| 16876764 | 0.028429264 | 1.82549 | up | radicalS-adenosylmethioninedomaincontaining2 |
| 16851397 | 0.029127898 | 1.815136 | up | retinoblastomabindingprotein8 |
| 17015975 | 0.017046748 | 1.815041 | up | thiopurineS-methyltransferase |
| 16825104 | 0.020310558 | 1.810342 | up | partnerandlocalizerofBRCA2 |
| 16917317 | 0.048582427 | 1.806544 | up |  |
| 16899086 | 0.018784093 | 1.793985 | up | bolAhomolog3(E.coli) |
| 16870776 | 0.04827589 | 1.790808 | up | zincfingerprotein826,pseudogene |
| 16897797 | 0.020585135 | 1.75157 | up | polyribonucleotidenucleotidyltransferase1 |
| 16774669 | 0.004103601 | 1.746791 | up | nudix(nucleosidediphosphatelinkedmoietyX)-typemotif15 |
| 16832852 | 0.001585936 | 1.743038 | up | ATPasefamily,AAAdomaincontaining5 |
| 16718553 | 0.007062209 | 1.735006 | up | DNAcross-linkrepair1A |
| 16820820 | 0.023199035 | 1.73456 | up | smallnucleolarRNA,C/Dbox111 |
| 16755750 | 0.011317682 | 1.731805 | up |  |
| 16707221 | 0.007060121 | 1.730919 | up | kinesinfamilymember20B |
| 16687467 | 0.012687255 | 1.729351 | up | heatshockproteinfamilyB(small),member11 |
| 16952451 | 0.021691605 | 1.729174 | up |  |
| 16878674 | 0.014659388 | 1.72127 | up |  |
| 16714998 | 0.019257735 | 1.704435 | up | DNAreplicationhelicase2homolog(yeast) |
| 16957769 | 0.016112795 | 1.7027 | up |  |
| 17102111 | 0.016218042 | 1.690846 | up | peroxiredoxin4 |
| 16813317 | 0.033823553 | 1.676192 | up |  |
| 16985079 | 0.025911635 | 1.675251 | up | NADHdehydrogenase(ubiquinone)complexI,assemblyfactor2 |
| 16793190 | 0.005095983 | 1.671032 | up | WDrepeatandHMG-boxDNAbindingprotein1 |
| 17011079 | 0.011233036 | 1.666352 | up | mannosidase,endo-alpha |
| 16751083 | 0.012538875 | 1.664785 | up | Laribonucleoproteindomainfamily,member4 |
| 16976108 | 0.04219697 | 1.664013 | up |  |
| 16916958 | 0.015958516 | 1.663216 | up | proliferatingcellnuclearantigen |
| 16747402 | 0.027029883 | 1.658294 | up | lymphocyte-activationgene3 |
| 17015771 | 0.031087061 | 1.65802 | up | coiled-coildomaincontaining90A |
| 16899050 | 0.03240025 | 1.646534 | up | TP53RKbindingprotein |
| 16830182 | 0.021252127 | 1.645329 | up | thioredoxindomaincontaining17 |
| 16941661 | 0.009888412 | 1.642119 | up | guaninenucleotidebindingprotein-like3(nucleolar)\|smallnucleolarRNA,C/Dbox19B |
| 16701748 | 0.012190883 | 1.639767 | up | GTPbindingprotein4 |
| 17092020 | 0.015133952 | 1.637232 | up |  |
| 16708552 | 0.02417755 | 1.635942 | up | nucleolarandcoiled-bodyphosphoprotein1 |
| 16890005 | 0.015870314 | 1.632565 | up | FASTkinasedomains2 |
| 16938296 | 0.012376676 | 1.631087 | up | SGOL1antisenseRNA1(non-proteincoding) |
| 17117537 | 0.046670392 | 1.627827 | up |  |
| 16777549 | 0.014915281 | 1.626422 | up | myotubularinrelatedprotein6 |
| 16743779 | 0.002942442 | 1.623592 | up | DCN1,defectiveincullinneddylation1,domaincontaining5(S.cerevisiae) |
| 16690511 | 0.017129887 | 1.623064 | up |  |
| 16662692 | 0.002456797 | 1.621902 | up | UTP11-like,U3smallnucleolarribonucleoprotein,(yeast) |
| 16800962 | 0.037628915 | 1.621378 | up | solutecarrierfamily27(fattyacidtransporter),member2 |
| 16851344 | 6.51E-04 | 1.620854 | up | smallnuclearribonucleoproteinD1polypeptide16kDa |
| 16924523 | 0.026650855 | 1.619847 | up | mitochondrialribosomalproteinL39 |
| 16779720 | 0.012744382 | 1.616671 | up | mitoticspindleorganizingprotein1 |
| 16824022 | 0.036309943 | 1.616617 | up |  |
| 16983800 | 0.00822169 | 1.612807 | up | threonyl-tRNAsynthetase |
| 16704844 | 0.02491808 | 1.608227 | up | translocaseofinnermitochondrialmembrane23homolog(yeast) |
| 16775421 | 0.012787399 | 1.603624 | up | ubiquitincarboxyl-terminalesteraseL3(ubiquitinthiolesterase) |
| 17098411 | 0.028374288 | 1.598077 | up | heatshock70kDaprotein5(glucose-regulatedprotein,78kDa) |
| 16677913 | 0.024159843 | 1.594251 | up | delta(4)-desaturase,sphingolipid1 |
| 16740630 | 0.030687992 | 1.58759 | up | FOS-likeantigen1 |
| 16809587 | 0.003792702 | 1.584612 | up | ribosomalL24domaincontaining1 |
| 16736554 | 0.043657415 | 1.582927 | up | SPT2,SuppressorofTy,domaincontaining1(S.cerevisiae) |
| 16729557 | 0.01664203 | 1.582169 | up | chromosome11openreadingframe82 |
| 16902357 | 0.017589664 | 1.58162 | up | MKI67(FHAdomain)interactingnucleolarphosphoprotein |
| 16840284 | 0.001181922 | 1.581453 | up | complementcomponent1,qsubcomponentbindingprotein |
| 16777384 | 7.45E-04 | 1.579014 | up | spasticataxiaofCharlevoix-Saguenay(sacsin) |
| 16889602 | 0.001744075 | 1.577514 | up |  |
| 16677547 | 0.02315972 | 1.576587 | up | ribosomalRNAprocessing15homolog(S.cerevisiae) |
| 17078342 | 0.04137138 | 1.573541 | up | transcriptionelongationfactorB(SIII),polypeptide1(15kDa,elonginC) |
| 16889209 | 0.018311232 | 1.572417 | up | chromosome2openreadingframe47 |
| 17004747 | 0.005030525 | 1.571572 | up | PAK1interactingprotein1 |
| 16708728 | 0.034594946 | 1.56249 | up | sideroflexin2 |
| 16807600 | 0.033270072 | 1.56029 | up |  |
| 17004836 | 0.02871365 | 1.552735 | up | chromosome6openreadingframe228 |
| 16883426 | 0.002267277 | 1.552529 | up | eukaryotictranslationinitiationfactor5B |
| 16664861 | 0.047834165 | 1.549253 | up |  |
| 17059628 | 0.014143405 | 1.546153 | up | cytochromeP450,family51,subfamilyA,polypeptide1\|leucine-richrepeatsanddeathdomaincontaining1 |
| 16745798 | 0.03356581 | 1.542997 | up | fasciculationandelongationproteinzeta1(zyginI) |
| 16796122 | 0.01526769 | 1.541623 | up | chromosome14openreadingframe142 |
| 16943254 | 0.0071397 | 1.53317 | up | chromosome3openreadingframe26 |
| 16780441 | 0.021829251 | 1.529193 | up |  |
| 16836131 | 0.001696292 | 1.523794 | up |  |
| 16986117 | 0.02239966 | 1.520494 | up |  |
| 16959325 | 0.033531755 | 1.518194 | up | topoisomerase(DNA)IIbindingprotein1 |
| 16806702 | 0.01407111 | 1.513335 | up | golginA8family,memberI,pseudogene\|uncharacterizedLOC100289637\|golginA8family,memberJ\|golginsubfamilyAmember8-likeprotein2-like |
| 16960775 | 0.029779918 | 1.511936 | up | signalsequencereceptor,gamma(translocon-associatedproteingamma) |
| 16951485 | 0.020488542 | 1.511463 | up | shugoshin-like1(S.pombe) |
| 16912192 | 0.015747236 | 1.509944 | up | GINScomplexsubunit1(Psf1homolog) |
| 16731169 | 0.007903609 | 1.509277 | up | dihydrolipoamideS-acetyltransferase |
| 16947556 | 0.029475253 | 1.501676 | up | structuralmaintenanceofchromosomes4 |
| 17096580 | 0.018926786 | 1.500992 | up | endoplasmicreticulumprotein44 |

| **Transcript Cluster Id** | **p value** | **FC (abs)** | **Regulation** | **Gene Description** |
| --- | --- | --- | --- | --- |
| 16903140 | 0.001147259 | 5.053075 | down | chemokine(C-X-Cmotif)receptor4 |
| 16698697 | 0.003378362 | 3.9796472 | down | Fasapoptoticinhibitorymolecule3 |
| 16914395 | 0.01384247 | 3.9209547 | down | matrixmetallopeptidase9(gelatinaseB,92kDagelatinase,92kDatypeIVcollagenase) |
| 16744967 | 0.005987146 | 3.5636346 | down | adhesionmolecule,interactswithCXADRantigen1 |
| 16695392 | 0.03311914 | 3.5144753 | down | SLAMfamilymember6 |
| 16850656 | 0.008070719 | 3.4863536 | down | uncharacterizedLOC649446 |
| 16732584 | 0.02130911 | 3.2574694 | down | sortilin-relatedreceptor,L(DLRclass)Arepeatscontaining |
| 16952118 | 0.042427704 | 2.991043 | down | tetratricopeptiderepeatandankyrinrepeatcontaining1 |
| 16918196 | 0.023329232 | 2.9196284 | down |  |
| 17111955 | 0.03461012 | 2.9114377 | down | chemokine(C-X-Cmotif)receptor3 |
| 16916261 | 0.025667137 | 2.8966675 | down | protein-L-isoaspartate(D-aspartate)O-methyltransferasedomaincontaining2 |
| 16709201 | 0.004537053 | 2.894649 | down | programmedcelldeath4(neoplastictransformationinhibitor)\|microRNA4680 |
| 16722299 | 0.011303109 | 2.7889132 | down | phosphodiesterase3B,cGMP-inhibited |
| 16914952 | 0.012257496 | 2.6650422 | down |  |
| 17056833 | 0.017987939 | 2.6512716 | down | smallproline-richprotein2F\|Tcellreceptorgammavariable9 |
| 16897026 | 0.029098922 | 2.615436 | down | zincfingerprotein36,C3Htype-like2 |
| 16695769 | 0.004983496 | 2.6129327 | down | SH2domaincontaining1B |
| 17012859 | 0.002914959 | 2.5246093 | down | phosphodiesterase7B |
| 16845856 | 0.009273907 | 2.4964423 | down |  |
| 17103185 | 0.039534073 | 2.472551 | down | TIMPmetallopeptidaseinhibitor1 |
| 17098698 | 0.044436783 | 2.4441812 | down | familywithsequencesimilarity102,memberA |
| 17053362 | 0.030296447 | 2.3926504 | down |  |
| 16672373 | 0.032581467 | 2.3729556 | down | pyrinandHINdomainfamily,member1 |
| 16825638 | 9.11E-05 | 2.3723044 | down | yippee-like3(Drosophila) |
| 16667760 | 0.002639025 | 2.3169076 | down | sphingosine-1-phosphatereceptor1 |
| 17056701 | 0.017007034 | 2.2985904 | down | acyloxyacylhydrolase(neutrophil) |
| 17118287 | 0.003557319 | 2.278607 | down |  |
| 16843511 | 0.048746016 | 2.2532723 | down | chemokine(C-Cmotif)ligand5 |
| 16748205 | 0.009171358 | 2.2151797 | down | C-typelectindomainfamily2,memberD |
| 16730845 | 0.039221913 | 2.2108262 | down | ataxiatelangiectasiamutated\|nuclearprotein,ataxia-telangiectasialocus |
| 16956983 | 0.036968306 | 2.1915374 | down | Cblproto-oncogene,E3ubiquitinproteinligaseB |
| 17056734 | 0.017962858 | 2.1446295 | down | AOAHintronictranscript1(non-proteincoding) |
| 16962584 | 0.014495391 | 2.1235857 | down | B-cellCLL/lymphoma6 |
| 16735152 | 0.003623313 | 2.119105 | down | tripeptidylpeptidaseI |
| 17023927 | 0.006752873 | 2.1085618 | down | Abelsonhelperintegrationsite1 |
| 16975333 | 0.019062476 | 2.104086 | down | chromosome4openreadingframe34 |
| 16901974 | 0.011658465 | 2.0897498 | down | interleukin1,alpha |
| 16850923 | 0.034139767 | 2.0385296 | down |  |
| 16925398 | 0.045795392 | 2.03214 | down | RUNX1intronictranscript1(non-proteincoding) |
| 16836697 | 0.016139863 | 2.0274942 | down | breastcarcinomaamplifiedsequence3 |
| 16954343 | 0.020954382 | 2.0100477 | down | RBM5antisenseRNA |
| 16995601 | 0.036907367 | 2.001599 | down | FYNbindingprotein |
| 17063835 | 0.016031796 | 2.0007424 | down |  |
| 17118104 | 0.042712953 | 1.9973589 | down | putativePOM121-likeprotein1-like |
| 16761326 | 0.03726406 | 1.9968802 | down |  |
| 16745467 | 0.03848774 | 1.9946866 | down |  |
| 17118058 | 0.043403905 | 1.993184 | down | putativePOM121-likeprotein1-like |
| 17118127 | 0.043913983 | 1.991462 | down | putativePOM121-likeprotein1-like |
| 17118066 | 0.04341266 | 1.9912959 | down | putativePOM121-likeprotein1-like |
| 17094814 | 0.029047722 | 1.9748472 | down | transmembraneprotein2 |
| 16756310 | 0.009213807 | 1.9712113 | down | t-complex11(mouse)-like2 |
| 16863667 | 0.032972157 | 1.9531977 | down | gliomatumorsuppressorcandidateregiongene2 |
| 16930132 | 0.036191493 | 1.9487933 | down |  |
| 16718622 | 0.009531005 | 1.9379351 | down | actinbindingLIMprotein1 |
| 16984010 | 0.018112259 | 1.9244941 | down | interleukin7receptor |
| 16989202 | 0.009180167 | 1.9192152 | down | transcriptionfactor7(T-cellspecific,HMG-box) |
| 16834578 | 0.004495394 | 1.9191201 | down | neighborofBRCA1gene1 |
| 16956149 | 0.017564438 | 1.9163011 | down | forkheadboxP1 |
| 17005685 | 0.038167354 | 1.9124285 | down | butyrophilin,subfamily3,memberA3 |
| 16675323 | 0.03928604 | 1.9002744 | down | regulatorofG-proteinsignaling2,24kDa |
| 16738694 | 0.01663957 | 1.8969299 | down | macrophageexpressed1 |
| 17010929 | 0.009165965 | 1.89462 | down | proline-richnuclearreceptorcoactivator1 |
| 16812886 | 0.001660759 | 1.8942086 | down |  |
| 16709732 | 0.04939301 | 1.8798131 | down |  |
| 17049237 | 0.001891766 | 1.878012 | down | poliovirusreceptorrelatedimmunoglobulindomaincontaining\|stromalantigen3 |
| 17014064 | 0.001255595 | 1.873588 | down | zincfinger,DHHC-typecontaining14 |
| 17086418 | 0.010707738 | 1.8735092 | down | kinesinfamilymember27pseudogene |
| 16702881 | 0.037678104 | 1.8656774 | down | mannosereceptor,Ctype1 |
| 16702836 | 0.022318326 | 1.8577266 | down | mannosereceptor,Ctype1 |
| 16832608 | 0.048285004 | 1.8527361 | down |  |
| 16821330 | 0.008418362 | 1.8517406 | down | phospholipaseC,gamma2(phosphatidylinositol-specific) |
| 17052652 | 0.018058259 | 1.8337237 | down | protease,serine,1(trypsin1)\|protease,serine,3pseudogene2 |
| 16954217 | 0.045535766 | 1.8305188 | down | ubiquitin-likemodifieractivatingenzyme7 |
| 16977820 | 0.023127127 | 1.8287506 | down | hydroxysteroid(17-beta)dehydrogenase11 |
| 16767744 | 0.048944026 | 1.825411 | down |  |
| 16993655 | 0.03212433 | 1.8197485 | down | uncharacterizedLOC402483\|uncharacterizedLOC100132062\|uncharacterizedLOC100133161\|uncharacterizedLOC729737\|uncharacterizedLOC441124\|uncharacterizedLOC100506479\|uncharacterizedLOC100289306\|uncharacterizedLOC100287894\|uncharacterizedLOC399844\|uncharacterizedLOC100653346\|uncharacterizedLOC100288102\|uncharacterizedLOC731275\|uncharacterizedLOC100653241\|uncharacterizedLOC100652945\|uncharacterizedLOC100508632\|uncharacterizedLOC100132050\|putativeuncharacterizedproteinFLJ44672-like |
| 16797869 | 0.01474111 | 1.8176974 | down | hectdomainandRLD2pseudogene7 |
| 17057931 | 0.020304851 | 1.8166543 | down | uncharacterizedLOC100506479\|uncharacterizedLOC100288102\|uncharacterizedLOC731275\|putativeuncharacterizedproteinFLJ44672-like\|uncharacterizedLOC100131871\|putativeuncharacterizedproteinencodedbyNCRNA00174-like |
| 16845222 | 0.013650875 | 1.8146232 | down | enhancerofzestehomolog1(Drosophila) |
| 16863589 | 0.006800336 | 1.8116572 | down | complementcomponent5areceptor1 |
| 16873060 | 0.029147852 | 1.8010893 | down | plasminogenactivator,urokinasereceptor |
| 16986583 | 0.004745015 | 1.7903296 | down | junctionmediatingandregulatoryprotein,p53cofactor |
| 17104768 | 0.005771899 | 1.7883117 | down | O-linkedN-acetylglucosamine(GlcNAc)transferase |
| 16720049 | 0.030632513 | 1.7873834 | down | ATH1,acidtrehalase-like1(yeast) |
| 17022362 | 0.044831246 | 1.7873696 | down | sestrin1 |
| 17049312 | 0.013581105 | 1.7870365 | down | pairedimmunoglobin-liketype2receptoralpha |
| 17013024 | 0.029463835 | 1.7862558 | down | coiled-coildomaincontaining28A |
| 17013283 | 0.01973574 | 1.781799 | down | Utrophin |
| 17081373 | 0.02410187 | 1.7813017 | down | Src-like-adaptor |
| 17066921 | 0.019817874 | 1.7808855 | down | ADAMmetallopeptidasedomain28 |
| 16868187 | 0.022381179 | 1.7773018 | down | myosinIF |
| 17077135 | 0.013756134 | 1.7679905 | down | RB1-induciblecoiled-coil1 |
| 16824208 | 0.004534196 | 1.7626048 | down |  |
| 16766051 | 0.007458964 | 1.7609855 | down |  |
| 16859763 | 0.041768007 | 1.7523012 | down | interferon,gamma-inducibleprotein30 |
| 17002846 | 0.041251212 | 1.7492582 | down | dualspecificityphosphatase1 |
| 16698466 | 0.024693724 | 1.7357627 | down | NUAKfamily,SNF1-likekinase,2 |
| 17043588 | 0.00646095 | 1.732129 | down | glucocorticoidinducedtranscript1 |
| 17076009 | 0.030710181 | 1.730314 | down | transmembraneprotein66 |
| 17081347 | 0.017082533 | 1.7297815 | down | transmembraneprotein71 |
| 16840321 | 0.003009329 | 1.7272563 | down | NLRfamily,pyrindomaincontaining1 |
| 16795943 | 0.03712434 | 1.7229407 | down | tandemC2domains,nuclear |
| 17005157 | 0.01193291 | 1.7193017 | down | familywithsequencesimilarity8,memberA1 |
| 16760005 | 0.048623197 | 1.7188628 | down | CACNA1CantisenseRNA2(non-proteincoding) |
| 16889636 | 0.033310942 | 1.7175014 | down | bonemorphogeneticproteinreceptor,typeII(serine/threoninekinase) |
| 16765404 | 0.001522739 | 1.7156509 | down | calciumbindingandcoiled-coildomain1 |
| 16761311 | 0.022788152 | 1.7155949 | down |  |
| 16721506 | 0.049303684 | 1.7129104 | down |  |
| 16661687 | 0.010136418 | 1.7025292 | down | erythrocytemembraneproteinband4.1(elliptocytosis1,RH-linked) |
| 17004154 | 0.001965711 | 1.7007555 | down | dualspecificityphosphatase22\|dualspecificityproteinphosphatase22-like |
| 16968797 | 0.03867301 | 1.6960411 | down | HECTandRLDdomaincontainingE3ubiquitinproteinligase3 |
| 16673943 | 0.022576818 | 1.6935107 | down |  |
| 16806870 | 0.030682737 | 1.6741892 | down | solutecarrierfamily12(potassium/chloridetransporters),member6 |
| 16853631 | 0.04543657 | 1.67331 | down | erythrocytemembraneproteinband4.1-like3 |
| 16711822 | 0.01912662 | 1.6729892 | down | microRNA4481 |
| 16737669 | 0.004852508 | 1.6699992 | down | PHDfingerprotein21A |
| 16692383 | 0.045274016 | 1.66997 | down | neuroblastomabreakpointfamily,member24\|neuroblastomabreakpointfamily,member11\|neuroblastomabreakpointfamily,member15\|neuroblastomabreakpointfamily,member16\|neuroblastomabreakpointfamily,member8\|neuroblastomabreakpointfamily,member9\|neuroblastomabreakpointfamily,member10\|neuroblastomabreakpointfamily,member12\|neuroblastomabreakpointfamily,member14\|neuroblastomabreakpointfamilymember21-like\|neuroblastomabreakpointfamily,member7 |
| 17098319 | 0.016514266 | 1.6679735 | down |  |
| 16860795 | 0.01517533 | 1.6648505 | down | zincfingerprotein181 |
| 16992467 | 0.001665818 | 1.6628065 | down | CREB3regulatoryfactor |
| 17047258 | 0.034690913 | 1.658649 | down | GATSprotein-like1\|GATSprotein-like2 |
| 16980364 | 0.012219978 | 1.6511176 | down | zincfingerprotein827 |
| 16827546 | 0.011451697 | 1.650473 | down | dipeptidase2 |
| 17117999 | 0.010871942 | 1.6477172 | down |  |
| 17042314 | 0.034631763 | 1.6459243 | down | pre-B-cellleukemiahomeobox2\|pre-B-cellleukemiahomeobox2pseudogene1 |
| 16805495 | 0.005583512 | 1.6452426 | down | insulin-likegrowthfactor1receptor |
| 16929741 | 0.003110854 | 1.6428332 | down | cytohesin4 |
| 17039818 | 0.035512514 | 1.6412379 | down | pre-B-cellleukemiahomeobox2\|pre-B-cellleukemiahomeobox2pseudogene1 |
| 17027017 | 0.034992553 | 1.6393322 | down | pre-B-cellleukemiahomeobox2\|pre-B-cellleukemiahomeobox2pseudogene1 |
| 17094334 | 0.034943867 | 1.6382214 | down |  |
| 16919242 | 0.006854188 | 1.6382025 | down | v-mafmusculoaponeuroticfibrosarcomaoncogenehomologB(avian) |
| 16748477 | 0.012433659 | 1.6374253 | down | cyclin-dependentkinaseinhibitor1B(p27,Kip1) |
| 16662688 | 0.040292628 | 1.6358865 | down |  |
| 16706259 | 0.010460733 | 1.6350838 | down | K(lysine)acetyltransferase6B |
| 16727277 | 0.009847793 | 1.6340706 | down | phosphofurinacidicclustersortingprotein1 |
| 16782132 | 0.030062761 | 1.6318847 | down | abhydrolasedomaincontaining4 |
| 16810884 | 0.04161633 | 1.6313692 | down |  |
| 16840318 | 0.034802433 | 1.6277559 | down | NLRfamily,pyrindomaincontaining1\|uncharacterizedLOC728392 |
| 16804333 | 0.00947608 | 1.6263754 | down | Akinase(PRKA)anchorprotein13 |
| 17086540 | 0.021748468 | 1.6251647 | down | cathepsinL1 |
| 16669708 | 0.01776573 | 1.6229624 | down | notch2N-terminallike |
| 17098626 | 0.005647307 | 1.619606 | down | adenylatekinase1 |
| 16865308 | 0.012404331 | 1.6156063 | down | leukocytereceptorcluster(LRC)member8 |
| 17017167 | 0.047128774 | 1.6089545 | down |  |
| 17092767 | 0.0498352 | 1.6030992 | down | myeloid/lymphoidormixed-lineageleukemia(trithoraxhomolog,Drosophila) |
| 17074673 | 0.00507931 | 1.6008685 | down | cathepsinB |
| 17047338 | 0.024458619 | 1.5987263 | down |  |
| 16916623 | 0.031996135 | 1.5985208 | down | PC-esterasedomaincontaining1A |
| 16837230 | 1.95E-04 | 1.5952867 | down | bromodomainPHDfingertranscriptionfactor\|RhoGTPaseactivatingprotein27pseudogene |
| 16809511 | 0.0340111 | 1.5924116 | down | familywithsequencesimilarity214,memberA |
| 16968077 | 0.010052305 | 1.5917937 | down | cyclinG2 |
| 16769514 | 0.007934319 | 1.5913723 | down | adaptorprotein,phosphotyrosineinteraction,PHdomainandleucinezippercontaining2 |
| 17034609 | 0.036803864 | 1.5854701 | down | pre-B-cellleukemiahomeobox2\|pre-B-cellleukemiahomeobox2pseudogene1 |
| 17029618 | 0.03725722 | 1.584467 | down | pre-B-cellleukemiahomeobox2\|pre-B-cellleukemiahomeobox2pseudogene1 |
| 17027749 | 0.047305606 | 1.5824544 | down | HLAcomplexgroup26(non-proteincoding) |
| 16984968 | 0.031268597 | 1.5786996 | down |  |
| 17030575 | 0.04751077 | 1.5757391 | down | HLAcomplexgroup26(non-proteincoding) |
| 16683605 | 0.036776286 | 1.5739511 | down | NIPA-likedomaincontaining3 |
| 17064197 | 0.031842906 | 1.5727904 | down | zincfingerfamilymember767 |
| 17051409 | 0.012284714 | 1.5695657 | down | adenosylhomocysteinase-like2 |
| 16689969 | 0.008741178 | 1.5694401 | down | dihydropyrimidinedehydrogenase |
| 16912472 | 0.022027979 | 1.5691249 | down | hemopoieticcellkinase |
| 17035373 | 0.047860943 | 1.5672346 | down | HLAcomplexgroup26(non-proteincoding) |
| 17067459 | 0.022444595 | 1.5659583 | down | homeoboxcontaining1 |
| 16744442 | 0.005703942 | 1.5653539 | down |  |
| 16845537 | 0.012142779 | 1.5616925 | down | histonedeacetylase5 |
| 16657920 | 0.03269744 | 1.5615815 | down |  |
| 16836868 | 0.03239591 | 1.559044 | down | tetratricopeptiderepeat,ankyrinrepeatandcoiled-coilcontaining2 |
| 17096188 | 0.006745601 | 1.5566497 | down | solutecarrierfamily35,memberD2 |
| 17090155 | 0.047386374 | 1.555063 | down |  |
| 16884441 | 0.025126677 | 1.5543846 | down | zincfingerCCCH-typecontaining6 |
| 16807848 | 0.042900857 | 1.549058 | down | vacuolarproteinsorting39homolog(S.cerevisiae) |
| 16777963 | 0.027398523 | 1.5483023 | down |  |
| 17069550 | 0.009248251 | 1.5477651 | down | alcoholdehydrogenase,ironcontaining,1 |
| 16891990 | 0.03026107 | 1.546158 | down | integralmembraneprotein2C |
| 16926253 | 0.04433422 | 1.5461285 | down | cystatinB(stefinB) |
| 17064285 | 0.028025556 | 1.5410515 | down | transmembraneprotein176B |
| 16657450 | 0.046178948 | 1.5410312 | down | uncharacterizedLOC100132062\|uncharacterizedLOC729737\|uncharacterizedLOC441124\|uncharacterizedLOC100506479\|uncharacterizedLOC100289306\|uncharacterizedLOC100287894\|uncharacterizedLOC399844\|uncharacterizedLOC100288102\|uncharacterizedLOC731275\|uncharacterizedLOC100653346\|uncharacterizedLOC100653241\|uncharacterizedLOC100652945\|uncharacterizedLOC100508632\|uncharacterizedLOC100132050\|putativeuncharacterizedproteinFLJ44672-like |
| 16880414 | 0.04871353 | 1.5408053 | down | v-relreticuloendotheliosisviraloncogenehomolog(avian) |
| 16907623 | 0.012553936 | 1.5395733 | down | Kruppel-likefactor7(ubiquitous) |
| 17039923 | 0.027834827 | 1.5385131 | down | majorhistocompatibilitycomplex,classII,DQbeta1\|HLAclassIIhistocompatibilityantigen,DQbeta1chain-like |
| 17029721 | 0.027633 | 1.5382589 | down | majorhistocompatibilitycomplex,classII,DQbeta1\|HLAclassIIhistocompatibilityantigen,DQbeta1chain-like |
| 16834921 | 0.024226688 | 1.5372243 | down | hexamethylenebis-acetamideinducible1 |
| 16801932 | 0.04441778 | 1.5367299 | down |  |
| 16783944 | 0.014342079 | 1.5332165 | down | kelchdomaincontaining2 |
| 16807342 | 0.0275592 | 1.5302271 | down | phospholipaseC,beta2 |
| 16825484 | 0.04287469 | 1.5300325 | down | nuclearporecomplexinteractingprotein-like3\|nuclearporecomplexinteractingproteinpseudogene\|nuclearporecomplexinteractingproteinrelatedgene\|nuclearporecomplex-interactingprotein-like3-like\|uncharacterizedLOC100652992 |
| 16748202 | 0.011609554 | 1.5299712 | down |  |
| 17105755 | 0.037136044 | 1.5286162 | down | transcriptionelongationfactorA(SII)-like3 |
| 16745113 | 0.007120974 | 1.5280269 | down | B-cellCLL/lymphoma9-like |
| 16829158 | 0.04524231 | 1.5258251 | down | ringfingerprotein166 |
| 16755339 | 0.024946636 | 1.525806 | down |  |
| 16701149 | 0.004693141 | 1.5242865 | down |  |
| 16845850 | 4.49E-04 | 1.5202634 | down |  |
| 16720987 | 0.018868044 | 1.5196832 | down |  |
| 16889664 | 0.006596187 | 1.5185198 | down | amyotrophiclateralsclerosis2(juvenile)chromosomeregion,candidate8 |
| 16704542 | 0.025202274 | 1.5147829 | down | familywithsequencesimilarity21,memberB\|familywithsequencesimilarity21,memberA\|familywithsequencesimilarity21,memberC |
| 16793263 | 0.03028419 | 1.5146508 | down | autophagyrelated14 |
| 17029712 | 0.032773655 | 1.5145494 | down | majorhistocompatibilitycomplex,classII,DRbeta1\|majorhistocompatibilitycomplex,classII,DRbeta3\|majorhistocompatibilitycomplex,classII,DRbeta4\|HLAclassIIhistocompatibilityantigen,DRB1-7betachain-like |
| 17039914 | 0.033992402 | 1.5144061 | down | majorhistocompatibilitycomplex,classII,DRbeta1\|majorhistocompatibilitycomplex,classII,DRbeta3\|majorhistocompatibilitycomplex,classII,DRbeta4\|HLAclassIIhistocompatibilityantigen,DRB1-7betachain-like |
| 16819355 | 0.044608466 | 1.512643 | down | NLRfamily,CARDdomaincontaining5 |
| 16852433 | 0.005563216 | 1.5122991 | down | polymerase(DNAdirected)iota |
| 16667801 | 0.03687493 | 1.5104246 | down | RNA-bindingregion(RNP1,RRM)containing3 |
| 16974074 | 0.032107536 | 1.5100831 | down |  |
| 16919295 | 0.013517519 | 1.509338 | down | chromodomainhelicaseDNAbindingprotein6 |
| 17044773 | 2.30E-04 | 1.5022886 | down | zincandringfinger2 |
| 17025438 | 0.005754533 | 1.5020401 | down | AGPAT4intronictranscript1(non-proteincoding) |
| 16948572 | 0.041078564 | 1.5005122 | down | kelch-like24(Drosophila) |
